# Unraveling the impact of ZZZ3 on the mTOR/ribosome pathway in human embryonic stem cells homeostasis

**DOI:** 10.1016/j.stemcr.2024.04.002

**Published:** 2024-05-02

**Authors:** Michela Lo Conte, Valeria Lucchino, Stefania Scalise, Clara Zannino, Desirèe Valente, Giada Rossignoli, Maria Stella Murfuni, Chiara Cicconetti, Luana Scaramuzzino, Danilo Swann Matassa, Anna Procopio, Graziano Martello, Giovanni Cuda, Elvira Immacolata Parrotta

**Affiliations:** 1 Department of Experimental and Clinical Medicine, University Magna Graecia, 88100 Catanzaro, Italy; 2 Department of Medical and Surgical Sciences, University Magna Graecia, 88100 Catanzaro, Italy; 3 Department of Life Sciences and Systems Biology, University of Turin, Via Nizza 52, 10126 Torino, Italy; 4 Italian Institute for Genomic Medicine (IIGM), 10060 Candiolo Torino, Italy; 5 Department of Department of Molecular Medicine and Medical Biotechnology, University of Naples Federico II, 80131 Naples, Italy; 6 Department of Biology (DiBio), University of Padua, Padua, Italy

**Keywords:** human embryonic stem cells, hESCs, ZZZ3, ribosome biogenesis, proliferation, mTOR signaling, translation, quiescence

## Abstract

Embryonic stem cells (ESCs) are defined as stem cells with self-renewing and differentiation capabilities. These unique properties are tightly regulated and controlled by complex genetic and molecular mechanisms, whose understanding is essential for both basic and translational research. A large number of studies have mostly focused on understanding the molecular mechanisms governing pluripotency and differentiation of ESCs, while the regulation of proliferation has received comparably less attention. Here, we investigate the role of ZZZ3 (zinc finger ZZ-type containing 3) in human ESCs homeostasis. We found that knockdown of *ZZZ3* negatively impacts ribosome biogenesis, translation, and mTOR signaling, leading to a significant reduction in cell proliferation. This process occurs without affecting pluripotency, suggesting that ZZZ3-depleted ESCs enter a “dormant-like” state and that proliferation and pluripotency can be uncoupled also in human ESCs.

## Highlights

• ZZZ3 plays a key role in hESC homeostasis
• ZZZ3 knockdown adversely affects ribosome biogenesis, translation, and mTOR signaling
• ZZZ3 depletion does not impact pluripotency but reduces cell proliferation
• ZZZ3-depleted ESCs enter a “dormant-like” state

## Teaser

In this study, Parrotta, Cuda, and colleagues elucidated the impact of silencing the zinc finger ZZ-type containing 3 (ZZZ3) gene on the proliferation of ESCs, highlighting its modulation of the mTOR and ribosome biogenesis pathways. Notably, despite the deceleration of the cell cycle induced by ZZZ3 silencing, pluripotency is preserved, indicating that ESCs have the capacity to enter a quiescent state characterized by attenuated biosynthetic processes.

## Introduction

Embryonic stem cells (ESCs) are characterized by their indefinite self-renewal and pluripotency, allowing differentiation into the three germ layers (Martin, 1981; Sheridan et al., 2012). This dynamic nature stems from large fluctuations in gene expression, shaping a diverse protein landscape (Gabut et al., 2020). While extensive research has focused on transcriptional networks governing ESC pluripotency (Chambers et al., 2007; Masui et al., 2007; Niwa et al., 2000; Tee and Reinberg, 2014), limited understanding exists regarding proliferation regulation. In mouse ESCs, pluripotency and proliferation can be independent processes, meaning that it is possible for mouse ESCs to maintain their pluripotent state without actively proliferating. This separation of pluripotency maintenance and cell division is facilitated by the mammalian target of rapamycin (mTOR) signaling pathway, which plays a pivotal role in orchestrating developmental pausing. Paused blastocysts display diminished mTOR activity, gene expression, and cellular proliferation while maintaining pluripotency (Bulut-Karslioglu et al., 2016). Additionally, genetic inactivation of mTORC1-specific protein raptor reduces proliferation without impacting pluripotency (Xu et al., 2022). Embryonic development arrest induces diapause, a reversible biosynthetic dormant state involving the Wnt/β-catenin signaling and Esrrb pathway (Carbognin et al., 2023; Fan et al., 2020). Cell growth and proliferation entail complex biosynthetic processes, including ribosome biogenesis and translation. The zinc finger ZZ-type containing 3, also known as ZZZ3, acts as a histone H3 reader within the ATAC (Ada2a-containing) complex. Previous studies have demonstrated its role in regulating ribosomal genes in both mouse embryonic stem cells (mESCs) and human adenocarcinoma cells (Fischer et al., 2021; Mi et al., 2018). Our study investigates the role of ZZZ3 in human embryonic stem cells (hESCs), independent of its association with the ATAC complex. Utilizing a combination of interactome, transcriptome, and cellular analyses, we demonstrated that ZZZ3 regulates hESCs proliferation by modulating the mTOR/ribosome biogenesis pathway. Intriguingly, *ZZZ3* knockdown (KD) led to a notable decrease in hESCs proliferation, while maintaining pluripotency and differentiation potential. This observation suggests the potential existence of a pluripotent state marked by biosynthetic quiescence occurring *in vitro* within hESCs.

## Results

### ZZZ3 interacts with ribosomal proteins and co-localizes in the nucleolus with fibrillarin

**Figure 1:**
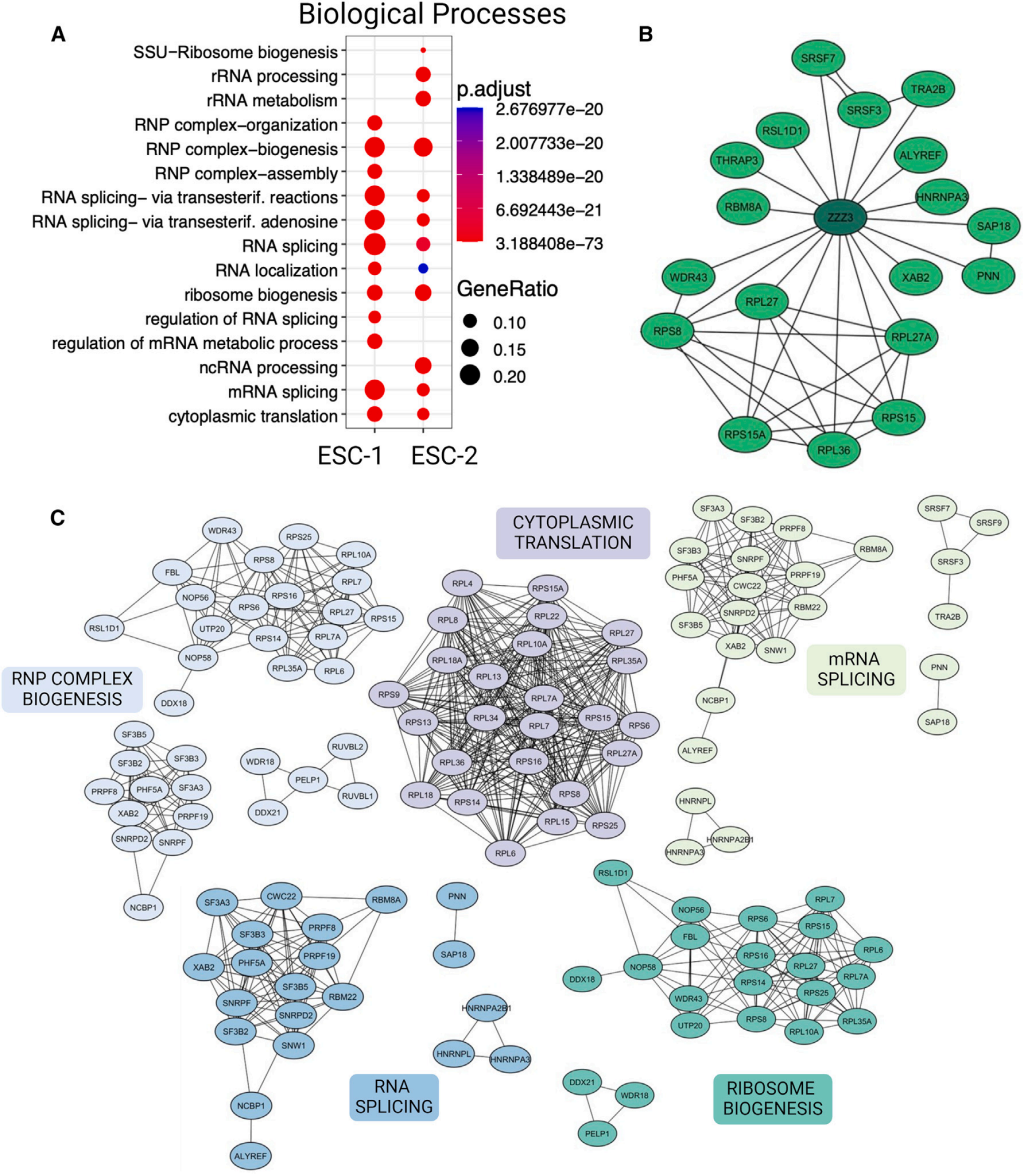
ZZZ3 interacts with proteins involved in cytoplasmic translation, ribosome biogenesis, RNP complex biogenesis, and RNA processing (A) Gene ontology (GO) enrichment analysis was performed on genes selected based on their significance (*p* value <0.05, corrected using the Benjamini-Hochberg procedure) and fold change (FC ≥ 2.5). The analysis was conducted in R, utilizing the Bioconductor package. The enriched biological processes (BPs) associated with the selected genes are presented. (B) The protein-protein interaction (PPI) network illustrates the relationships among the top differentially expressed genes (DEGs) with FC ≥ 10. This network highlights DEGs enriching the BP with the highest gene ratio in ESC-1 and ESC-2. Edges in the network represent known interactions between DEGs and ZZZ3. (C) PPI networks were constructed for all members of the top five BPs with the highest gene ratio in ESC-1 and ESC-2. The networks were generated using the Cytoscape software (version 3.10.0).

To characterize the ZZZ3 biological network in hESCs, we conducted a co-immunoprecipitation (IP) assay using an anti-ZZZ3 antibody. The co-precipitated proteins were subsequently identified through nanoscale liquid chromatography coupled to tandem mass spectrometry (nanoLC-MS/MS). Experiments were conducted using two lines of hESCs, WA17 and RUES, denoted herein as ESC-1 and ESC-2, respectively. Through the application of the Perseus software for statistical analysis, we identified 474 proteins that co-purified with ZZZ3 in ESC-1 and 744 proteins co-purified with ZZZ3 in ESC-2. Candidate ZZZ3 interactors were chosen based on statistically significant variances (*p* value <0.05 corrected using the Benjamini-Hochberg procedure) and fold change (FC) values of at least 2.5 compared to samples immunoprecipitated with control IgG. Gene Ontology (GO) enrichment analysis highlighted several significantly overrepresented annotations for biological processes linked to ribosome biogenesis, rRNA processing, rRNA metabolism, RNP complex biogenesis, RNP complex assembly, and cytoplasmic translation (Figure 1A). In the Cellular Component GO hierarchy, the small subunit processome, spliceosome complex, and ribosome emerged as the most prominent terms. Meanwhile, in the molecular function category, enrichment was observed in terms including structural constituent of ribosome, rRNA binding, snRNA binding, and helicase activity (Figures S1A and S1B; Table S_GO). Furthermore, analysis of protein-protein interaction (PPI) networks among proteins associated with ZZZ3 (FC >10), alongside the enrichment analysis of biological processes characterized by elevated scores, enabled the identification of numerous ribosomal protein components. Specifically, these included constituents of the 40S subunit such as RPS8, RPS15, and RPS15A, as well as components of the 60S subunit such as RPL27, RPL27A, and RPL36 (Figure 1B). The principal biological processes enriched by ZZZ3-interacting proteins are depicted in the PPI network (Figure 1C). Given that ribosome biogenesis primarily occurs in the cell’s nucleolus, we employed immunostaining to identify the co-expression of ZZZ3 and fibrillarin (FBL) in wild-type ESC-1 and -2. FBL is a nucleolar protein actively engaged in pre-rRNA processing, pre-rRNA methylation, and ribosome assembly (Rodriguez-Corona et al., 2015; Tollervey et al., 1993). The intensity-based correlation analysis, performed utilizing the Pearson’s correlation coefficient, revealed strong co-localization of ZZZ3 and FBL within the nucleolus of hESCs (Figure S1C). Consistent with prior research associating ZZZ3 with the regulation of ribosomal gene expression (Fischer et al., 2021; Mi et al., 2018), we found that many ZZZ3 interactors are indeed ribosomal proteins and that ZZZ3 co-localized with FBL in the nucleolus. Validation of the interaction between ZZZ3 and co-precipitated proteins was accomplished via IP followed by western blot analysis, as illustrated in Figure S1D.

### Pluripotency and differentiation remain uncompromised upon ZZZ3 deficiency

**Figure 2:**
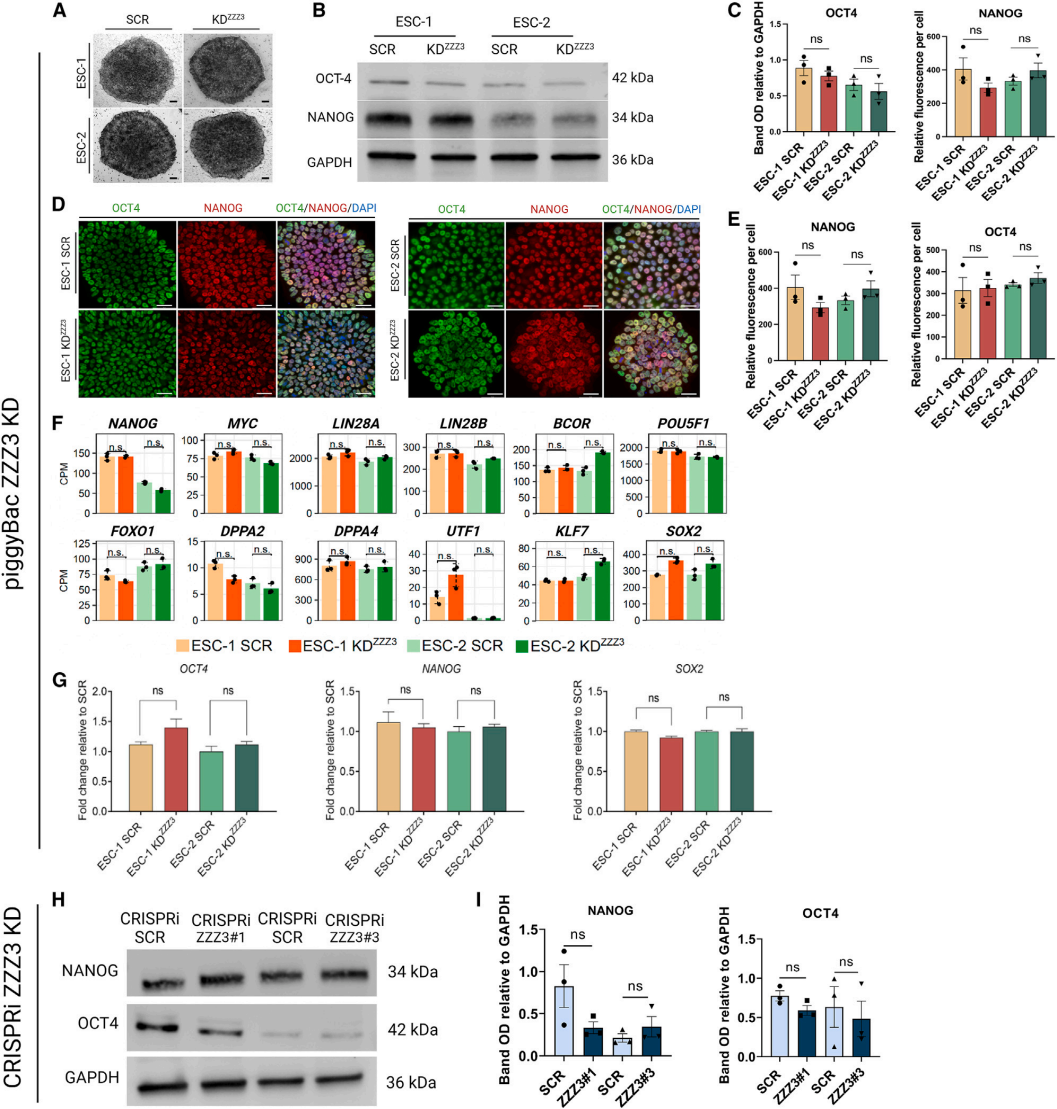
Pluripotency and differentiation are kept intact upon ZZZ3 KD (A) Alkaline phosphatase staining of SCR control and ZZZ3 KD ESCs. Scale bar 200 μm. (B) Representative western blot images of the OCT4 and NANOG expression and relative quantification (C). (D) Representative fluorescence microscopy images of SCR control and ZZZ3 KD hESCs for OCT4 and NANOG. Nuclei were counterstained using DAPI. Scale bar, 50 μm. (E) Quantification of fluorescence intensity using ImageJ software. (F) Barplots show the gene expression levels of a panel of pluripotency regulators in SCR and ZZZ3 KD hESCs as determined by RNA-seq analysis. Mean expression levels ± SEM from three independent experiments is represented by bars, with individual data points overlaid as dots. Statistical analysis was performed using ANOVA, *p* > 0.05. (G) qRT-PCR analysis of the core pluripotency factors OCT4, NANOG, and SOX2. (H) Western blot image showing the expression of NANOG and OCT4 in SCR control ZZZ3 and KD hESCs generated via CRISPRi system and relative quantification (I). Data are presented as mean ± SEM from 3 independent experiments. Statistical significance was determined by t test, ns = *p* > 0.05. GAPDH was used as loading control. Full-length blots are shown in File S1.

Pluripotent ESCs exhibit high proliferative activity, necessitating rapid ribosome biogenesis to sustain their growth and proliferation. As ZZZ3 interacts with and potentially regulates genes encoding ribosomal proteins, we aimed to probe the effect of ZZZ3 depletion on hESCs’ pluripotency. To address this, we employed stable KD of ZZZ3 expression in ESC-1 and ESC-2 lines using piggyBac (PB) vectors (Figure S2A). Three distinct PB short hairpin RNAs (PB-shRNAs) were employed independently to achieve silencing of *ZZZ3*; shRNA#2 was selected for subsequent experiments due to its high KD efficiency in both ESC lines (Figure S2B). Furthermore, we generated ZZZ3 KD hESCs using a CRISPR-interference (CRISPRi) PB vector as an alternative method for *ZZZ3* silencing, thus providing an orthogonal system (Figures S2C and S2D). Initially, we evaluated the expression of alkaline phosphatase (AP) to assess the proportion of undifferentiated cells between ZZZ3 KD and SCR control ESCs. We observed no discernible differences in either AP activity or cell morphology between ZZZ3 KD ESCs and control cells (Figure 2A). Pluripotency is stabilized by a triad of interconnected pluripotency transcription factors, namely OCT4, SOX2, and NANOG, that cooperatively regulate gene expression (Li and Izpisua Belmonte, 2018; Ng and Surani, 2011). Quantitative immunoblotting and immunostaining analyses confirmed the sustained expression of OCT4, NANOG, and SOX2 proteins in ZZZ3 KD ESCs (Figures 2B–2E). Gene expression analysis via RNA sequencing (RNA-seq) of a panel of pluripotency regulator genes, including *OCT4*, *SOX2*, *NANOG*, *MYC*, *LIN28A*, *LIN28B*, *BCOR*, *FOXO1*, *DPPA2*, *DPPA4*, *UTF1*, and *KLF7*, revealed a comparable expression pattern between modified and control ESCs (Figure 2F). Furthermore, the expression levels of *OCT4*, *NANOG*, and *SOX2* mRNA were assessed via quantitative reverse-transcription polymerase chain reaction in ZZZ3 KD ESCs. Once more, comparative analysis revealed no discernible differences in mRNA expression between the control (SCR) and ZZZ3 KD ESCs (Figure 2G). Immunoblot analysis was conducted to examine the expression levels of OCT4, NANOG, and SOX2 in ESCs transfected with CRISPRi-ZZZ3. Remarkably, no significant differences were detected in their relative expression compared to control cells (Figures 2H and 2I). To investigate further in this direction, as additional means for pluripotency, we analyzed the capacity of ZZZ3 KD hESCs to form embryoid bodies (EBs) and differentiate properly. We focused on early steps of differentiation such as the emergence of the three germ layers using a commercial kit (R&D Systems). Both SCR and ZZZ3 KD ESCs resulted comparably positive for SOX17, OTX2, and BRACHYURY as shown by quantitative immunofluorescence analysis (Figure S3A). Additionally, gene expression analysis by RNA-seq of a panel of three germ layer genes unveiled a consistent expression pattern in both SCR and ZZZ3 KD ESCs (Figure S3B). Finally, we extended the analysis assessing the expression changes for specific target genes (*PDGFRA, SOX9*, and *S100B* for ectoderm, *PECAM*, *ACTA*, and *HAND1* for mesoderm, and *DLX5*, *HTATSF1*, and *GATA6* for endoderm) via qPCR on EBs at day 10 of differentiation (Figure S3C). Additionally, we assessed the differentiation capabilities of hESCs following ZZZ3 CRISPRi-mediated silencing, employing it as an orthogonal system. Specifically, we examined the emergence of the three germ layers by immunostaining the cells with antibodies targeting SOX17, OTX2, and BRACHYURY in both SCR and CRISPR-ZZZ ESCs (Figure S3D). Once more, our observations revealed no significant alterations in the expression patterns of the analyzed markers. These assays allowed us to conclude that hESCs with ZZZ3 KD can undergo differentiation in a manner similar to that of control cells providing additional confirmation that depleting ZZZ3 does not impair pluripotency.

### KD of ZZZ3 induces a “dormant-like” pluripotent status

**Figure 3:**
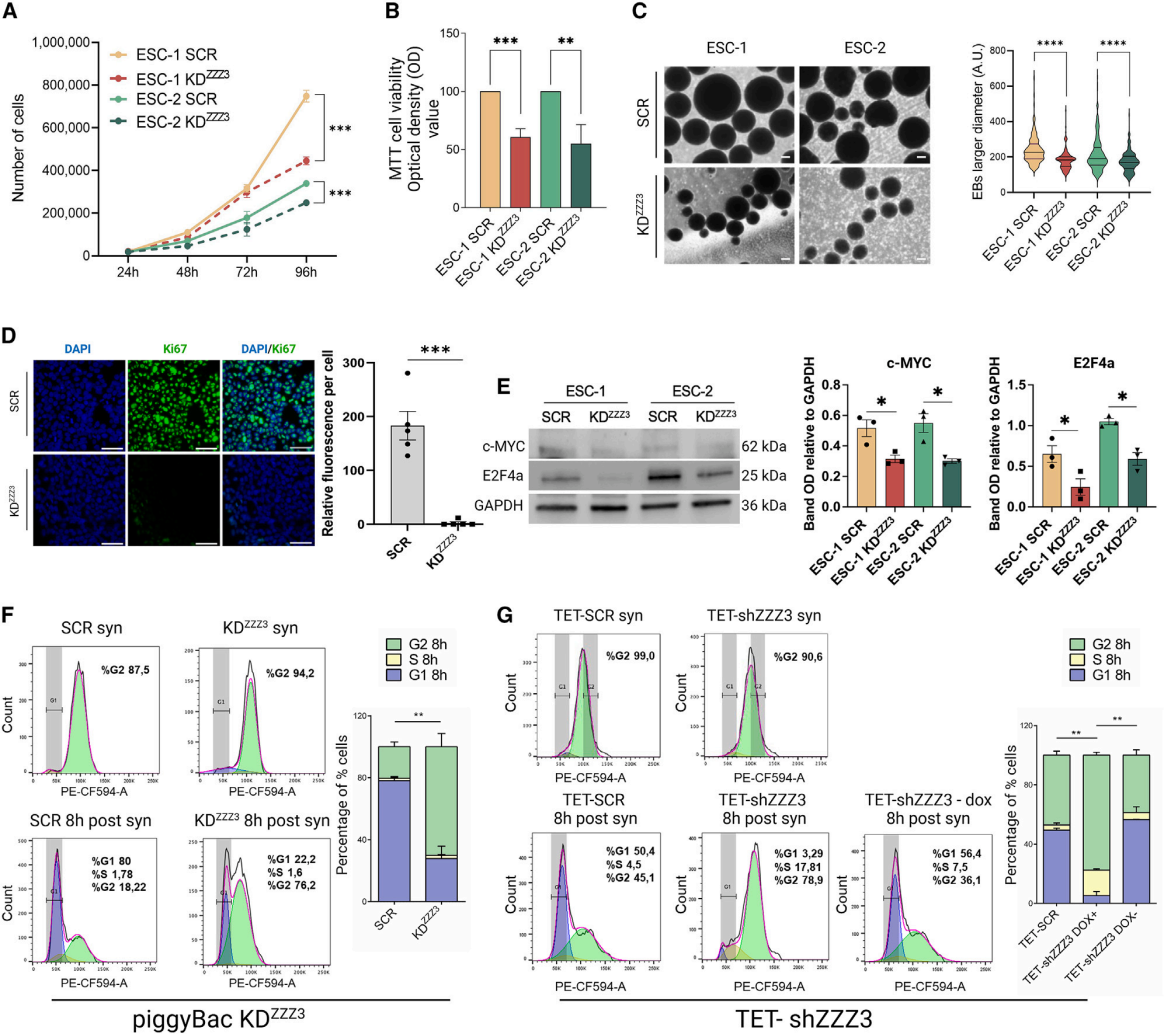
ZZZ3 knockdown in ESCs results in reduced proliferation (A) Cell proliferation was monitored over a 96-h time course from 3 independent experiments. The initial cell density was 3 × 10ˆ4 cells/well. (B) OD at 570 nm was measured to determine MTT reduction in ZZZ3 KD ESCs. (C) Phase-contrast images depict EBs generated from SCR and ZZZ3 KD ESC-1 and ESC-2. Measurement of EB size reveals a reduced diameter of EBs from ZZZ3 KD ESCs. Scale bar 200 μm. 150 EBs per line were measured as shown in violin plots. (D) Representative images depict immunostaining of the proliferation marker Ki67, showcasing a significant reduction in ZZZ3 KD cells. Nuclear DNA was counterstained with DAPI (blue). Scale bar, 50 μm. Relative fluorescence per cell measured for Ki67 immunostaining in ZZZ3 KD and control cells is shown to the right. At least 200 nuclei were analyzed per cell line. (E) Western blot images illustrate the expression of c-MYC and E2F4a in SCR control and ZZZ3 KD hESCs and relative OD quantification. GAPDH was used as a loading control. Full-length blots are shown in File S1. (F) Cell cycle profiles were measured by flow cytometry using propidium iodide. The analysis was conducted on SCR and ZZZ3 KD hESCs synchronized in the G2/M phase using nocodazole (upper panels) and at 8 h post-nocodazole withdrawal (lower panels). Histograms show the distribution of cells across the G0/G1, S, and G2/M phases. Quantitative analysis highlights a significant increase in G2/M phase of ZZZ3 KD cells. (G) Cell cycle profile of Tet-SCR and TET-shZZZ3 hESCs (Doxycycline [dox]-induced depletion of ZZZ3). Cells were synchronized in G2/M phase with nocodazole for 12 h. Subsequently, the cell cycle analysis was performed immediately after synchronization (syn) and after 8h of nocodazole withdrawal (8 h post-syn). Quantitative analysis reveals a significant increase in G2/M phase cells in TET-shZZZ3 cells in the presence of DOX (+DOX). Conversely, TET-shZZZ3 hESCs culture in the absence of doxycycline (-DOX) reverts to a state resembling SCR cell. Data are presented as means ± SEM or SD of 2 or 3 independent experiments and significance was calculated relative to the SCR control using t test, ^∗^*p* ≤ 0.05, ^∗∗^*p* ≤ 0.01, ^∗∗∗^*p* ≤ 0.001, ^∗∗∗∗^*p* ≤ 0.0001.

Pluripotency and proliferation are two processes in mESCs that can be uncoupled. Indeed, during diapause, pluripotency is kept intact although proliferation is strongly reduced. Because ZZZ3 interacts with ribosomal proteins and might be involved in the regulation of ribosome biogenesis, which occurs synergistically with cell growth and proliferation, we further wondered what was the effect of ZZZ3 KD on ESCs proliferation. Cell proliferation analysis, performed by cell counting at specific time points (24, 48, 72, and 96 h) and MTT assay, revealed that ZZZ3-depleted cells were significantly less proliferative compared with control ESCs (Figures 3A and 3B). The size of EBs correlates with proliferative capacity of ESCs, and cells with faster cell cycle progression are more likely to contribute to larger EBs (Hwang et al., 2009). We measured the diameter of EBs generated from ZZZ3 KD and SCR control hESCs and observed that modified cells generated smaller EBs (Figure 3C). Next, we performed immunostaining for Ki67, a protein involved in the cell cycle regulation, thus serving as a cellular marker for cell proliferation (Bullwinkel et al., 2006; Sun et al., 2017). We found that the expression of Ki67 is dramatically reduced in ZZZ3 KD hESCs (Figure 3D). Additionally, the expression of c-MYC and E2F4a, markers of cell proliferation tested via immunoblot, resulted in a significant decrease in silenced cells (Figure 3E). To investigate deeper in this direction, following synchronization of the cell cycle in the G2/M phase with nocodazole, we performed flow cytometry to assess the distribution of cells across the G1, S, and G2 phases in ZZZ3 KD hESCs compared to SCR controls. Cells with ZZZ3 KD showed a delay in cell cycle progression, particularly at the G2/M phase (Figure 3F). In the attempt to rescue the proliferation defect, we conducted cell cycle analysis using ZZZ3 KD hESCs generated with a PB vector encoding for doxycycline-inducible ZZZ3 KD (TET-shZZZ3) (Figures S2E and S2F). Ninety-six hours (96 h) after doxycycline withdrawal and ZZZ3 re-upregulation, cells exhibit enhanced progression through the cell cycle, transitioning more readily from the G2/M phase at T0 (synchronized) to the S and G1 phases (Figure 3G), suggesting that the “dormant status” observed in ZZZ3 KD hESCs can be rescued through the re-upregulation of ZZZ3.

### ZZZ3 is associated with ribosome biogenesis and translation

**Figure 4:**
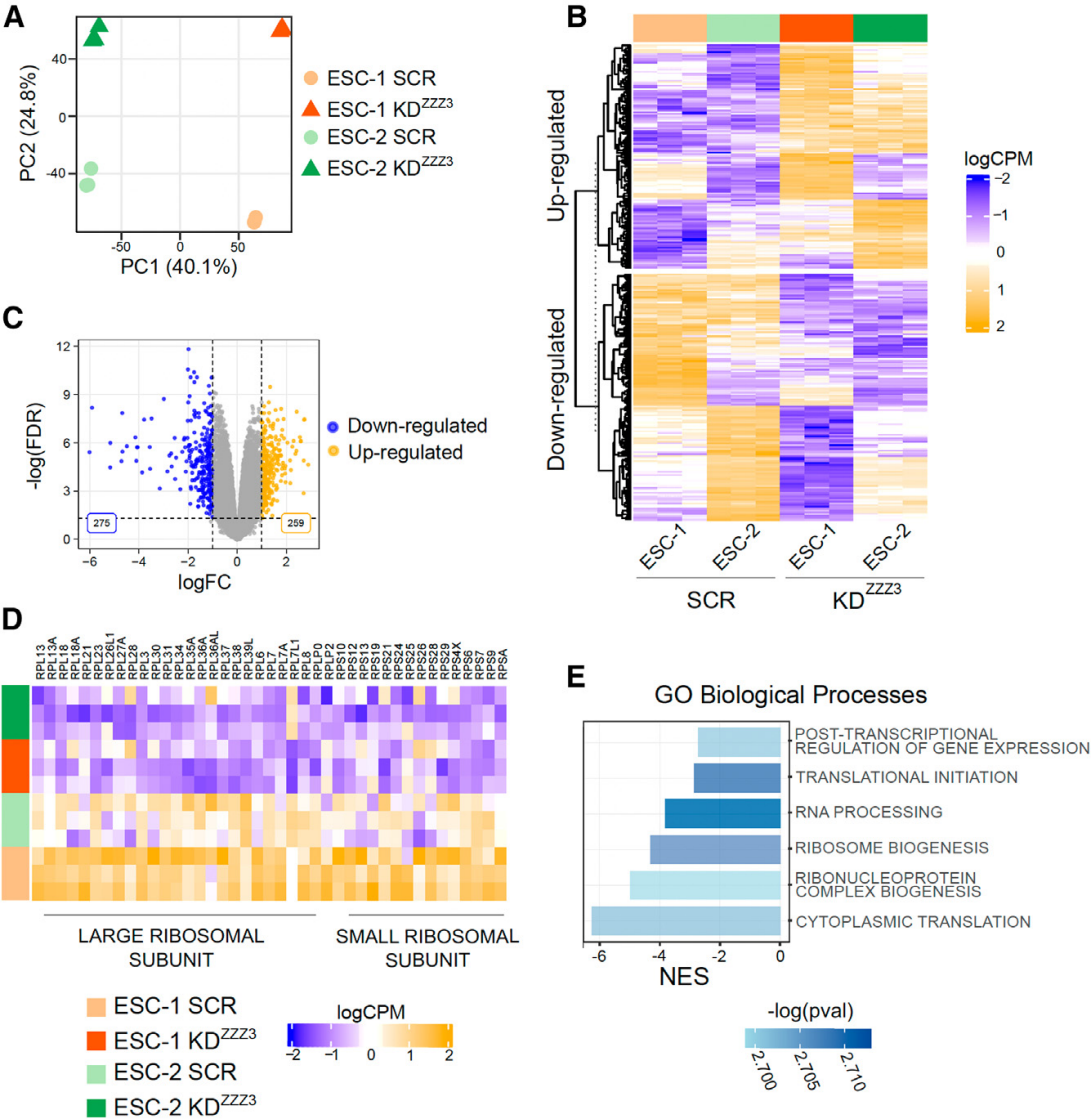
ZZZ3 KD in hESCs alters transcription of genes associated with ribosome biogenesis and translation (A) PCA of the logCPM gene expression data. (B) Heatmap of the DEGs between ZZZ3 KD ESCs and SCR condition (*Z* score scaled logCPM) identified with thresholds by applying the following thresholds: |logFC| > 1 and adjusted *p* value <0.05. DEGs are clustered according to a hierarchical clustering procedure based on Euclidean distance. (C) Volcano plot of DEGs in ZZZ3 KD ESCs vs. SCR control. Blue dots represent downregulated genes (275) while yellow dots represent upregulated genes (259). The axes represent the logFC and the adjusted *p* value related to each gene. (D) Heatmap of the Z-scaled logCPM expression values of genes encoding small and large ribosomal proteins. (E) GO BPs gene sets significantly associated with the ZZZ3 KD condition as resulted from the GSEA performed on the logFC^∗^-log(*p*-value) ranked genes. The normalized enriched score (NES) is reported for each gene set having different color shades according to the -log(*p* value).

To gain insight into the functional and regulatory roles of ZZZ3 in hESCs, we employed RNA-seq analysis on ZZZ3 KD ESCs. As a first step, we performed a dimensionality reduction procedure that evidenced how a good fraction of the transcriptomic variability is connected with the ESCs identity (Figure 4A). Nevertheless, the second principal component separated the samples according to the ZZZ3 state, suggesting a common effect of the KD on the ESCs. We took into account this ESCs intrinsic variability by performing a differential expression analysis with a paired design (for details see experimental procedures section), thus identifying the genes whose expression was affected by the ZZZ3 KD in both lines of hESCs. Analysis of RNA-seq data comparing SCR hESCs to ZZZ3 KD hESCs revealed significant alterations in gene expression. Specifically, ZZZ3 deficiency was associated with an increase in the expression of 259 genes and a decrease in the expression of 275 genes (FC ≥2 a false discovery rate < 0.05) (Figures 4B and 4C; Table S2). Notably, many of the genes downregulated in ZZZ3 KD hESCs encode for small and large ribosomal proteins (Figure 4D). Gene set enrichment analysis (GSEA) revealed that many of the downregulated genes were linked to biological processes including post-transcriptional regulation of gene expression, translational initiation, RNA processing, ribosome biogenesis, ribonucleoprotein complex biogenesis, and cytoplasmic translation (Figure 4E). Taken together, the results of both interactome and transcriptome analyses suggest that ZZZ3 not only regulates the transcription of ribosomal genes but also interacts with ribosomal proteins, indicating a crucial role for ZZZ3 in the ribosome biogenesis process.

### ZZZ3 KD impairs ribosome biogenesis and translation and triggers p53 activation

**Figure 5:**
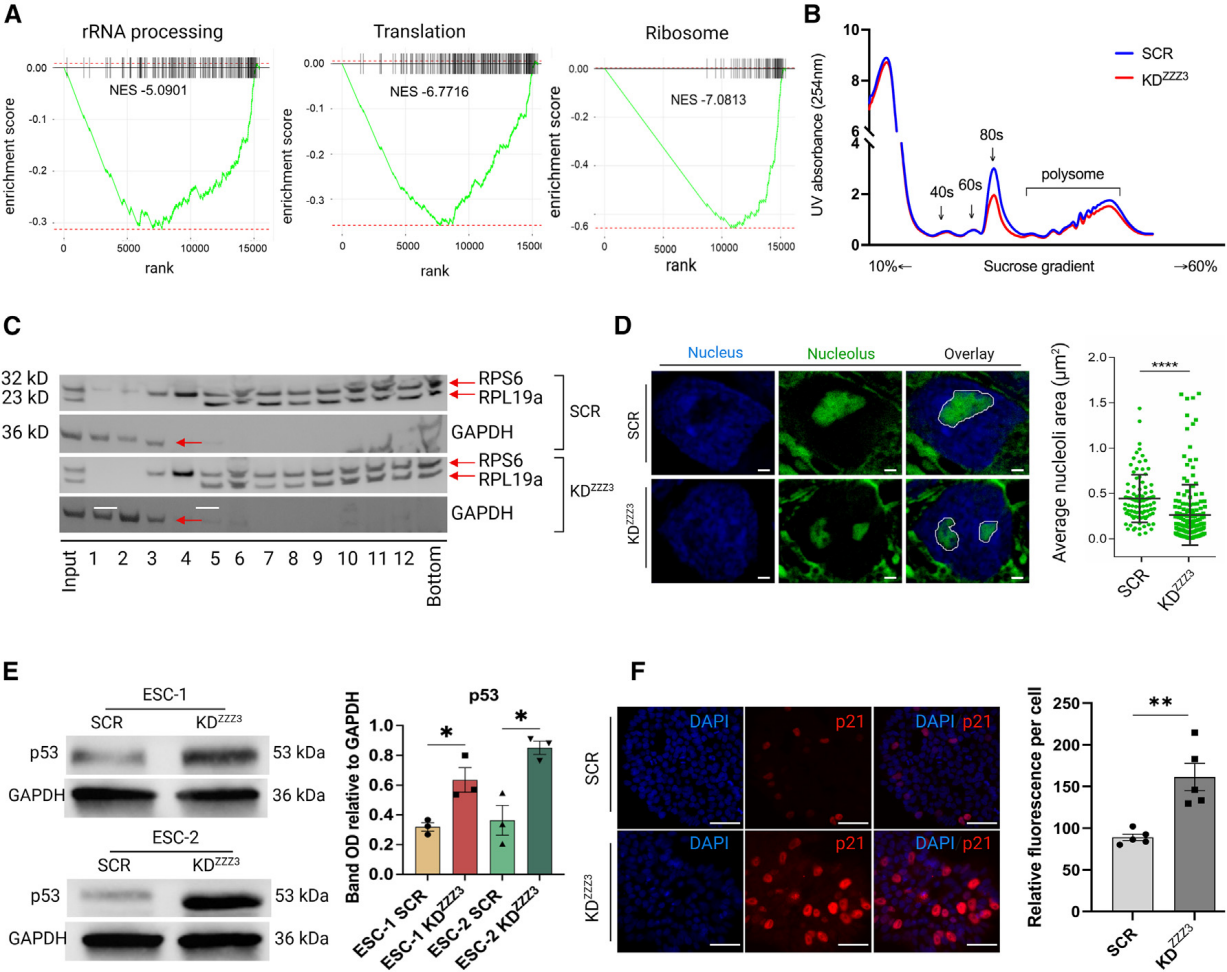
ZZZ3 KD impairs ribosome biogenesis and translation, triggering p53 activation (A) GSEA enrichment plots show the relative enrichment of gene sets related to rRNA processing (NES = −5.0901), translation (NES = −6.7716), and ribosome (NES = −7.0813) in ZZZ3 KD. The adjusted *p* value (padj) for both gene sets is equal to 0.019. (B) Polysome profiling absorbance, measured at 254 nm, was conducted on ZZZ3 KD cells and SCR control cell extracts fractionated on a 10%–60% sucrose gradient. Peaks corresponding to free 40S and 60S subunits, 80S monosomes, and polysomes are indicated on the gradient. (C) Immunoblot analysis on equal volumes of whole-cell polysome fractions. RPS6 and RPL19a were used as references for small and large ribosomal subunits, respectively. GAPDH served as a marker for proteins not associated with ribosomes. The numbers below the image refer to different fractions as follows: free ribosome (1–3), 40S subunit (4), 60S subunit (5), 80S monosomes (6), and polysomes (7–12). Full-length blots are provided in File S1. (D) Representative image for nucleolar staining (green) showing a reduced size of nucleoli in ZZZ3 KD cells compared to control. Nuclear DNA was counterstained with DAPI (blue). Scale bar, 1 μm. The measurement of the area of all nucleoli counted within a total of 80 nuclei from 3 independent experiments using ImageJ software. Scatterplots on the right indicate the value of individual cellular measurements, with the horizontal line indicating the mean. Statistical significance was computed as unpaired two-tailed t test of means, ^∗∗∗∗^*p* < 0.0001. (E) Quantitative immunoblot analysis of p53 demonstrates increased expression in ZZZ3 KD hESCs. GAPDH was utilized as a loading control. Full-length blots are available in File S1. (F) Representative immunofluorescence staining for p21 reveals increased expression in ZZZ3 KD ESCs. DNA was counterstained with DAPI (blue). Scale bar, 50 μm. Quantification of p21 (200 nuclei analyzed) immunofluorescence signals was performed using ImageJ software. Data are presented as mean ± SEM and significance was calculated using t test, ^∗^*p* ≤ 0.05, ^∗∗^*p* ≤ 0.01.

Ribosome biogenesis is a well-orchestrated process involving the coordinated actions of three types of RNA polymerase (RNAP I, RNAP II, and RNAP III) that make up the active ribosomes, machinery of translation (Jiao et al., 2023). GSEA analysis of transcriptomic data revealed that rRNA processing (normalized enriched score [NES] = −5.09), translation (NES = −6.7), and ribosome (NES = −7.08) are some of the most downregulated biological processes in ZZZ3 KD cells (Figure 5A). Polysome profiling analysis was employed to measure the translation dynamics in ZZZ3 KD ESCs. This assay revealed a clear reduction in the 80S and polysomes fractions in cells with depletion of ZZZ3 compared to SCR ESCs (Figure 5B). Immunoblot analysis for RPL19a and RPS6 was used to validate protein distribution in polysome profiling subfractions (1–12) (Figure 5C). rRNA synthesis and ribosome biogenesis primarily occur within the nucleolus and an impairment of these processes can lead to changes in the size of this subcellular structure. Notably, our research revealed that ZZZ3 KD hESCs exhibit a modified nucleolar structure, characterized by reduced size (Figure 5D). This alteration potentially reflects disturbances in ribosome biogenesis. Moreover, an impaired ribosome biogenesis can trigger nucleolar stress via a p53-dependent mechanism (Gilkes et al., 2006). We measured the expression level of p53 and its direct target p21 using immunoblot and immunoassay, respectively. Both were dramatically increased in ZZZ3 KD ESCs (Figures 5E and 5F) indicative of a stress condition taking place. Interestingly, the increase in p53 and p21 levels in ZZZ3 KD hESCs, along with the delay in cell cycle progression, prompts us to investigate whether apoptosis was active. Analysis via western blot of critical apoptotic markers, namely CASPASE-3 and cleaved CASPASE-9, along with qPCR evaluation of *BIM*, *BAX*, and *FAS* genes, revealed no notable variances between ZZZ3 KD hESCs and the control cells (Figures S4A and S4B, respectively). Moreover, TUNEL assay analysis did not reveal any noticeable differences between scramble and ZZZ3 KD cells (Figure S4C). Collectively, these outcomes, in conjunction with data from Ki67 immunostaining, cell proliferation, and cell cycle analysis, indicate that the decreased cell proliferation observed in ZZZ3 KD cells is unlikely to be attributed to cell death.

### ZZZ3 regulates cell proliferation and translation through the PI3K-AKT-mTOR axis

**Figure 6:**
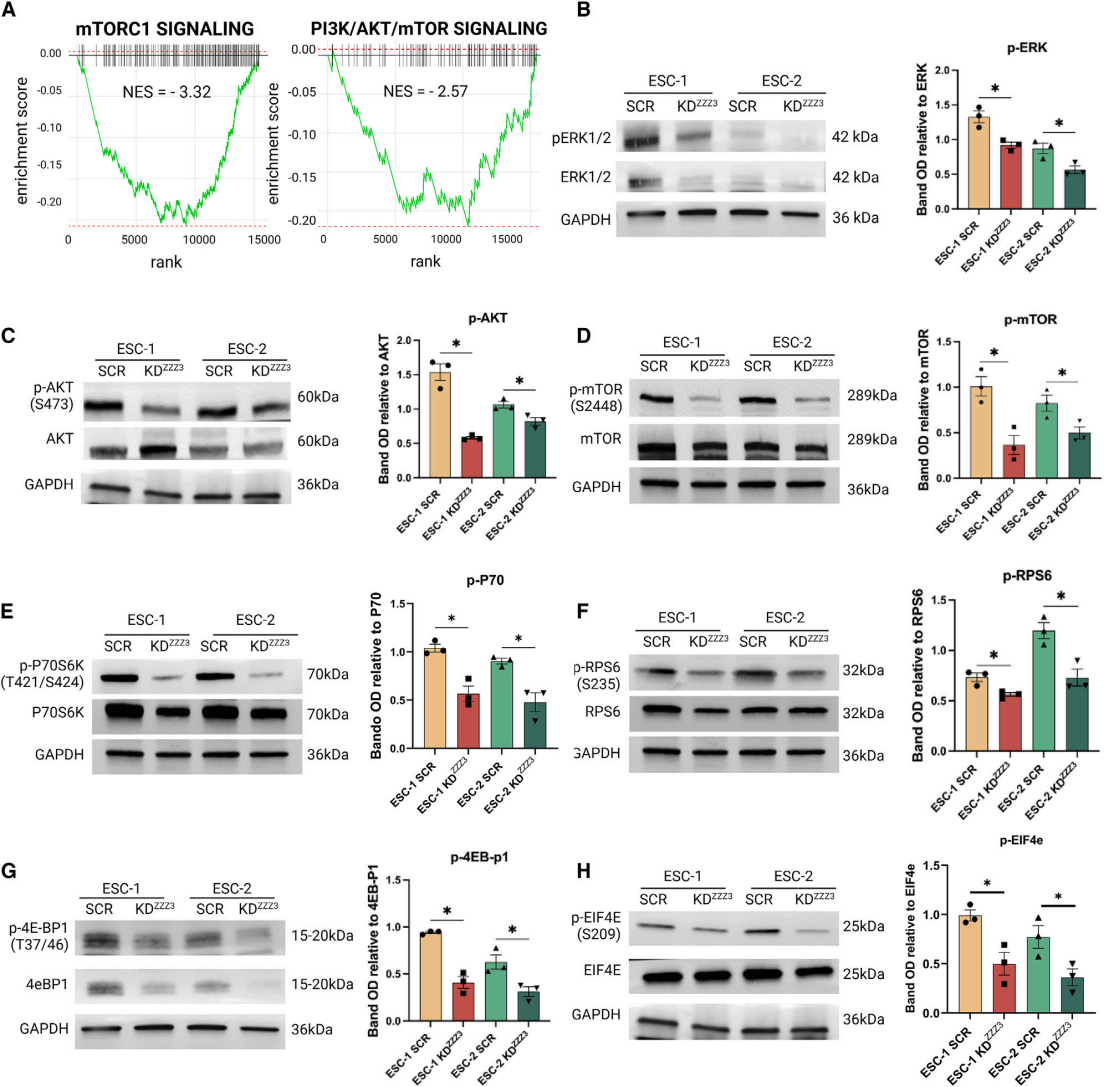
KD of ZZZ3 reduces the PI3K-AKT-mTOR axis (A) GSEA plots show the relative enrichment of gene sets associated with mTORC1 (NES = −3.32) and PI3K/AKT/mTOR (NES = −2.57) pathways in ZZZ3 KD hESCs. The adjusted *p* value (padj) for both gene sets is equal to 0.0057. (B–H) Quantitative immunoblot analysis was performed for p-ERK 1/2 (Thr202/Tyr204) and total ERK 1/2 (B); pAKT (Ser473) and total AKT (C); pmTOR (Ser2448) and total mTOR (D); pP70S6 Kinase (Thr421/Ser424) and total P70S6 kinase (E); pRPS6 (Ser235) and total RPS6 (F); p4E-BP1 (Thr37/46) and total 4E-BP1 (G); peIF4E (Ser209) and total eIF4E (H). GAPDH was used as loading control. Full-length blots are available in File S1. Quantification of protein expression was performed using OD measurement. Data are presented as mean ± SEM from *n* = 3 independent experiments. Significance was calculated vs. relative SCR ESCs using t test, ^∗^*p* ≤ 0.05.

In the attempt to molecularly connect the reduced cell proliferation and impaired ribosome biogenesis observed in ZZZ3 KD ESCs, we focused on the ERK (extracellular signal-regulated kinase) and mTOR, the latter crucially involved in the regulation of translation initiation in mammalian cells. The PI3K-AKT-mTORC1 signaling pathways were significantly enriched by genes downregulated upon ZZZ3 depletion (Figure 6A). The convergence of signals from the mTOR and PI3K pathways provides an integrated mechanism for cells controlling the rate of protein synthesis and cell growth (Roux et al., 2007). ERK is a member of the mitogen-activated protein kinase family, which plays a central role in cell proliferation. The phosphorylated ERK (p-ERK) serves as an indicator of the activation status of the ERK pathway and positively correlates with the rate of cell proliferation (Morrison, 2012). Immunoblot analysis for total ERK1/2 and p-ERK (Thr202/Tyr204) revealed that their expression is significantly reduced in ESCs with ZZZ3 KD (Figure 6B). The mTOR pathway plays an important role in regulating various cellular processes, including ribosome biogenesis, by modulating processing of rRNA and the translation of ribosomal proteins, as well as activating the RNA polymerase I transcription machinery (Mayer and Grummt, 2006). mTOR activation stimulates protein synthesis primarily through the phosphorylation and regulation of downstream targets, including components of the translation machinery, such as ribosomal protein S6 kinase (S6K1 or p70S6 kinase) (Holz et al., 2005), leading to the phosphorylation of its downstream targets, such as the ribosomal protein S6 (RPS6), a critical step in the initiation of protein translation (Bohlen et al., 2021; Jastrzebski et al., 2007). Immunoblot analysis of the active phosphorylated AKT(Ser473), which activates mTOR by phosphorylation on its Ser2448, confirmed a reduced expression of both in ZZZ3 KD ESCs (Figures 6C and 6D). The main downstream targets of mTOR signaling, phosphorylated P70S6K (T421/424) and phosphorylated RPS6 (Ser235), were also significantly reduced in ESCs with ZZZ3 depletion (Figures 6E and 6F). The inactivation by phosphorylation of 4E-BP1 (eukaryotic initiation factor 4E-binding protein 1) on T37/42 is another key mechanism by which mTOR promotes protein synthesis (Böhm et al., 2021). We found that the inactive form of 4E-BP1 is less expressed in ZZZ3 KD cells (Figure 6G) as well as its downstream target eukaryotic initiation factor 4E (Figure 6H). S6K phosphorylates several targets involved in translation initiation, leading to increased ribosome biogenesis and translation initiation complex assembly. In an effort to restore these dysregulated signaling pathways to their baseline levels observed in SCR control cells, we treated ZZZ3-silenced cells with insulin-like growth factor-1 (IGF-1), known to activate the PI3K/Akt pathway (Liu et al., 2017), upstream of the mTOR signaling pathway. Western blot analysis of ZZZ3 KD hESCs treated with IGF-1 demonstrated a rescue of the PI3K-AKT-mTOR axis (Figures S5A–S5F). These results collectively suggest that the PI3K, ERK, AKT, and mTOR signaling pathways exhibit reduced activity in ESCs with ZZZ3 KD. Importantly, this diminished activation is reversible, as evidenced by its restoration upon stimulation with IGF-1.

## Discussion

Human pluripotent stem cells (PSCs), comprising ESCs and induced PSCs, possess the unique capability to self-renew and differentiate into any of the three germ layers (Evans and Kaufman, 1981; Parrotta et al., 2019a; Thomson et al., 1998). These features make them valuable for diverse biomedical applications including enhancing our understanding of early human development (Zhu and Huangfu, 2013), disease modeling (Lucchino et al., 2022; Orban et al., 2015; Scalise et al., 2022), drug discovery (Chen et al., 2018), regenerative medicine (Parrotta et al., 2019b), and personalized medicine (Shi et al., 2017). To harness their potential, understanding the molecular mechanisms governing pluripotency is crucial. While much research has focused on transcriptional regulation (Chambers et al., 2007; Masui et al., 2007; Niwa et al., 2000) and mechanisms governing differentiation and cellular fate (Chen and Hu, 2017), the relationship between proliferation and pluripotency remains a key question. Studies on diapaused mouse blastocysts and mESCs suggest that pluripotency and proliferation can be independent, challenging the notion of their inseparable connection (Bulut-Karslioglu et al., 2016; Carbognin et al., 2023; Fan et al., 2020; Scognamiglio et al., 2016). In this context, the mTOR pathway, associated with high translation output, emerges as a significant player. Notably, reduced protein synthesis is observed in diapause blastocysts, indicating a link between translation regulation and stem cell dormancy (Fu et al., 2014; Scognamiglio et al., 2016; Zoncu et al., 2011). Recent investigations reported that inhibition of mTOR can delay the progression of human blastoids and PSCs (Iyer et al., 2023). Here, we focused on ZZZ3, a protein shown to be implicated in regulating genes encoding ribosomal proteins in mESCs and human adenocarcinoma cells (Fischer et al., 2021; Mi et al., 2018). To elucidate the specific role of ZZZ3 in hESCs, we employed a targeted approach by downregulating its expression. Subsequently, we conducted a comparative analysis, contrasting the features of cells exhibiting diminished ZZZ3 expression with those maintaining normal levels of ZZZ3. ZZZ3 KD in hESCs did not affect pluripotency markers or differentiation capabilities. However, a substantial decrease in proliferation, as evidenced by reduced Ki67 and cell cycle analysis, was observed. The reduction in proliferative capacity was associated with downregulation in the ERK/PI3K/AKT/mTOR signaling pathway, crucial for cell cycle progression and growth. Further analysis revealed that ZZZ3 depletion led to impaired ribosome biogenesis, nucleolar shape remodeling, and p53 activation. The observed elevation in p53 levels and its downstream target, p21, in ZZZ3-depleted cells hints at a potential protective mechanism against impaired ribosome biogenesis. This phenomenon could serve as a promising avenue for future investigation. In summary, this research uncovers three main findings: first, proliferation can be uncoupled from pluripotency in hESCs *in vitro*; second, ZZZ3 is crucial for maintaining a high proliferative state by ensuring proper ribosome biogenesis and translation through modulation of the PI3K/AKT/mTOR pathway; and third, ZZZ3 depletion leads to impaired ribosome biogenesis and p53 activation, preventing the propagation of cells with compromised protein synthesis.

## Experimental procedures

### Resource availability

#### Lead contact

Further information and requests for resources and reagents should be directed to and will be fulfilled by the lead contact, Giovanni Cuda (cuda@unicz.it).

#### Materials availability

Reagents generated in this study are available form the lead contact without restriction.

#### Data and code availability

Raw data generated by next-generation sequencing platforms, with per-base quality scores (e.g., BAM, SFF, and HDF5 files or paired FASTQ/QUAL files), were deposited in the Gene Expression Omnibus (GEO) dataset under the accession number GEO: GSE242489. LC-MS/MS proteomic data were completely submitted to the PRIDE ProteomeXchange database under the accession number PRIDE: PXD047165.

### Generation of ZZZ3 KD ESCs

hESCs with stable KD of ZZZ3 gene were generated by transfecting cells with PB transposon plasmids system with PB transposase expression vector pBase. Three PB U6-promoter-driven shRNAs against ZZZ3 mRNA or scramble control were purchased from Vector Builder. For DNA transfection, hESCs were dissociated as single cells using TrypLE Select Enzyme (1X) (Thermo Fisher Scientific), and 250,000 cells were co-transfected with PB constructs (550 ng) and pBase plasmid (550 ng) using FuGENE HD transfection reagent (3.9 μL) (Promega), following the manufacturer’s instruction. Cells were cultured onto Matrigel-coated 12-well plate in mTeSR1 Plus medium (STEMCELL Technologies, Vancouver, Canada) with 10 μM Y27632 (ROCKi, Rho-associated kinase inhibitor, Selleckchem). After 48 h, transfected cells were selected with hygromycin B (200 μg/mL, Thermo Fisher Scientific) diluted in mTeSR1 Plus for 2 weeks before performing experiments. The sequences of PB ZZZ3 shRNA are provided in the Table S3.

### Plasmids for CRISPRi and cloning strategy

The CRISPRi system, comprising the pPB-Ins-TRE3Gp-KRAB-dCas9-ecDHFR-IRES-GFP-EF1Ap-Puro (Addgene #183410; https://www.addgene.org/183410/) and pPB-Ins-U6p-sgRNAentry-EF1Ap-TetOn3G-IRES-Neo plasmids (Addgene #183411; https://www.addgene.org/183411/), employs a DOX-inducible approach with KRAB domain fused to a catalytically inactive Cas9 (KRAB-dCas9), alongside a DHFR protein degron, stabilized by trimethoprim (TMP) treatment. An IRES-EGFP fragment downstream of the KRAB-dCas9 allows for tracking of CRISPRi induction. The PB gRNA delivery and transactivation plasmid contains a sgRNA driven by a U6 promoter and a TET-on 3G-IRES-Neomycin resistance cassette under the control of a constitutive EF1α promoter (Tang et al., 2022). Three sgRNAs targeting the ZZZ3 coding sequence promoter, along with a non-targeting sgRNA control, were designed using the CRISPR Design Tool (http://crispr.mit.edu/) and synthetized by the Eurofins genomics (https://eurofinsgenomics.eu/). The sgRNA sequences are available in Table S3. The cloning process started with the digestion of 10μg of the sgRNA delivery and transactivation plasmid using the Esp3I restriction enzyme. After digestion, the plasmid underwent dephosphorylation to remove phosphate groups, followed by purification. Each pair of sgRNA oligos was phosphorylated, annealed, and ligated into the digested plasmid using DNA ligase. The ligated DNA was transformed into DH5ɑ bacteria cells, and individual colonies were selected. Plasmid DNA was isolated from each colony and sequenced to confirm the presence of desired sequences and the integrity of the construct before using it for ESCs Neon transfection.

### Neon transfection

hESCs were dissociated into single cells using Accutase and resuspension in Neon Resuspension Buffer R. Each electroporation involved combining cells with 2 μg of plasmid DNA and pulsing twice with specific parameters (voltage of 1,100 and a width of 30 ms). Post-electroporation, cells were plated onto Matrigel-coated plates with mTeSR1 medium. Selection with puromycin (0.5 μg/mL), hygromycin (200 μg/mL), or neomycin (200 μg/mL) was applied 48 h after electroporation.

### Generation of CRISPRi ESCs

ESCs were initially co-transfected with the hyperactive PB transposase (helper PBase) plasmid and the PB-based CRISPRi to generate stable ESC lines carrying integrated CRISPRi via puromycin selection. Subsequently, CRISPRi ESCs were co-transfected with the hyperactive PB transposase plasmid and sgRNA delivery and transactivation. Stable ESC lines carrying integrated CRISPRi and sgRNA transgenes were established through puromycin (0.5 μg/mL) and neomycin (200 μg/mL) selection. CRISPRi induction was achieved with Dox addition and monitored by EGFP fluorescence. ZZZ3 interference efficiency was evaluated using immunoblot analysis (Figure S2D, with TET-inducible ZZZ3 silencing sequences provided in Table S3. CRISPRi induction and interference efficiency at 72 and 96 h were comparable in presence or absence of TMP, thus TMP was withdrawn.

### Transfection of ESCs with PB inducible ZZZ3 expression vector (Tet-On)

The ESCs were co-transfected with the hyperactive PB transposase (helper PBase) plasmid and the following PiggyBac Inducible ZZZ3 Expression Vectors (Tet-On) purchased from Vector Builder (https://en.vectorbuilder.com/): VB231123-1578zqe (Scramble); VB231126-1293tdh (ZZZ3 shRNA#1); VB231123-1572zpv (ZZZ3 shRNA#2). These vectors contain the miR30 sequence facilitating the formation of mature shRNA for knockdown. Transfection was performed using the Neon electroporation system using the procedure and plasmid DNA concentration described earlier. Stable ESC lines carrying the shRNA transgenes were established through hygromycin selection (200 μg/mL). Silencing of ZZZ3 was achieved in the presence of doxycycline for 96 h.

### EBs formation assay

EBs differentiation was performed as previously described in the study by Scaramuzzino et al. (2021). Bright-field images of EBs were captured using an imaging system (DMi8, Leica Microsystems). SCR and ZZZ3 KD ESCs were further differentiated into ectoderm, mesoderm, and endoderm using media supplements provided by a commercial kit (R&D Systems) according to the manufacturer’s instructions. Differentiated cells were stained with anti-human SOX17 antibody for endoderm, anti-human BRACHYURY antibody for mesoderm, and anti-human OTX2 antibody for ectoderm. Subsequently, cells were stained with 557-conjugated Donkey anti-IgG secondary antibody (red), and the nuclei were counterstained with DAPI.

### RNA-seq library preparation and analysis

Total RNA was extracted using TRIzol reagents (Invitrogen, 15596026) according to the manufacturer’s protocol. For library preparation, the quantity and quality of the starting RNA were checked by Qubit and Bioanalyzer (Agilent). 1 μg of total RNA was subjected to poly(A) enrichment and library preparation using the TruSeq Stranded mRNA Library Prep Kit (Illumina) following the manufacturer’s instructions. Libraries were sequenced on Illumina NextSeq 1000 System (paired-end 60 + 60 bp reads). After quality controls with FastQC (https://www.bioinformatics.babraham.ac.uk/projects/fastqc/), raw reads were aligned to the human reference genome (hg38/GRCh38) using STAR 2.7.1a (Dobin et al., 2013) (with parameters–outFilterMismatchNmax 999 –outFilterMismatchNoverLmax 0.04). Gene expression levels were quantified with featureCounts v1.6.3 (Liao et al., 2014) (options: -t exon -g gene_name) using GENCODE 32 annotation. ZZZ3 is annotated in GENCODE as AC118549.1. Multi-mapped reads were excluded from quantification. Gene expression counts were next analyzed using the edge*R* package (Robinson et al., 2010). After low expressed genes filtration (1 count per million (CPM) in less than 3 samples), normalization factors were calculated using the trimmed-mean of M-values method implemented in the calcNormFactors function, and CPM was obtained using normalized library sizes. Differential expression analysis was carried out by considering a paired design thus fitting a GLM with the formula “∼ 0 + condition + cell_type”. A QLF test was performed to compare the “ZZZ3 KD - SCR” groups. Genes were marked as significantly differentially expressed when having | logFC | > 1 and adjusted *p* value <0.05 (Benjamini-Hochberg *p* value correction). Principal component analysis of the expression dataset was performed using the Prcomp function implemented in the R stats package. Gene expression heatmaps with hierarchical clustering based on euclidean distance were generated using the ComplexHeatmap R package scaling logCPM values as *Z* scores across samples (Gu et al., 2016). GSEA was conducted by using GSEA software (Subramanian et al., 2005) on logFC^∗^-log(*p* value) ranked genes.

### IP

Subconfluent cells were harvested and cross-linked using 0.05 mM DSP (Thermo Fisher Scientific) for 2 h on ice. The reaction was quenched using 20 mM Tris-HCl, pH 7.4, for 15 min on ice, followed by two washes with PBS. Cells were centrifuged at 5,000 rpm for 5 min at 4°C and resuspended in IP buffer (50 mM sodium phosphate pH 7.2, 250 mM NaCl, 0.1% Triton X-100, 0.1 nM ZnCl_2_) supplemented with protease and phosphatase inhibitor cocktails (Thermo Fisher Scientific). The cell suspension was then sonicated with a Diagenode Bioruptor (Settings: 10 s ON, 10 s OFF, high power) and centrifuged at 13,000 rpm for 20 min at 4°C. Protein concentration was determined using the Bradford assay. 1 mg of cell lysate was immunoprecipitated overnight at 4°C under rotation with Dynabeads Protein A (1.5 mg) (Thermo Fisher Scientific) and 10 μg anti-ZZZ3 antibody (Abcam) or 10 μg anti-IgG antibody (Diagenode). The next day, the immunoprecipitated complexes were washed three times with an IP buffer. Precipitated proteins were either trypsin-digested for mass spectrometry analysis or denatured in a Laemmli sample buffer at 95°C for 5 min and analyzed by western Blot. For ZZZ3 interactome by nanoLC-MS/MS, IP using anti-ZZZ3 antibody was performed in biological quadruplicate.

### Mass spectrometry

ZZZ3 and its interactors were released from the magnetic beads by pre-digestion in 100 μL of digestion buffer (100 mM Tris-HCl pH 8.5, 0.1% Triton X-100, 400 ng trypsin Proteomics Grade [Sigma-Aldrich]) for 15 min at 37°C with shaking (500 rpm). After on-bead pre-digestion by trypsin, the supernatant was collected and subjected to tryptic digestion. Proteins were reduced by 10 mM dithiothreitol (DTT) for 1 h at 37°C under shaking (500 rpm). Cysteines were alkylated by treatment with 24 mM iodoacetamide for 1 h at 37°C with agitation (500 rpm) in the dark. The alkylation reaction was quenched by adding 2 mM DTT (final concentration) for 30 min at 37°C. Finally, proteins were incubated with 200 ng trypsin overnight at 37°C with agitation to complete digestion (Bernaudo et al., 2015). The resulting tryptic peptides (20 μL corresponding to approximately 20 μg of peptides) were purified by strong cation exchange StageTips method (Rappsilber et al., 2007) to remove eventual detergent residues. Before loading on the StageTips, the digested samples were acidified by adding 80% acetonitrile-0.5% formic acid (wash solution 2). The StageTip was conditioned by adding 20% acetonitrile-0.5% formic acid (wash solution 1, 50 μL) and 50 μL of W2. Peptides were loaded by slowly letting the fluid pass through one plug using a benchtop centrifuge. After 2 washes with 50 μL of Solution W2 and 50 μL of Solution W1, respectively, peptides were eluted by adding 7 μL of 500 mM ammonium acetate-20% acetonitrile, diluted with 33 μL of formic acid 0.1% and analyzed by nanoLC-MS/MS.

### LC-MS/MS analysis

Peptides were separated by an Easy nLC-1000 chromatographic instrument coupled to a Q-Exactive “classic” mass spectrometer (both from Thermo Fisher Scientific). All the liquid chromatography-tandem mass spectrometry (LC-MS/MS) analyses were carried using a linear gradient of 75 min at a flow rate of 230 nL/min on a 15 cm, 75 μm i.d., in-house-made column packed with 3 μm C18 silica particles (Dr. Maisch). The binary gradient was performed using mobile phase A (0.1% FA, 2% ACN) and mobile phase B (0.1% FA and 80% ACN). Peptide separation was obtained at a flow rate of 230 nL/min and ramped using from 3% B to 40% B in 60 min, from 40% to 100% in 13 min; the column was cleaned for 5 min with 100% of B. LC-MS/MS analysis were performed in data-dependent acquisition (DDA) using a top-12 method. Full-scan m/z range was 350–1800, followed by MS/MS scans on the 12 most intense precursor ions. DDA analysis was performed with resolution for full MS scan of 70,000 and of 35,000 for MS/MS scan; the isolation window was 1.6 m/z. AGC target for full MS was 1e6 and 1e5 for MS/MS scan. The maximum injection time was set to 50 ms for full MS scans and 120 ms for MS/MS scans. Fragmentation was performed by higher-energy collisional dissociation (HCD) using 25% normalized collision energy and a dynamic exclusion of 20s. The mass spectrometry proteomics data have been deposited to the ProteomeXchange Consortium via the PRIDE (Perez-Riverol et al., 2022) partner repository.

### Statistical analysis

Experimental data are presented as means ± standard deviation of the mean unless stated otherwise. Statistical significance was calculated unless stated otherwise by two-tailed unpaired t test on two experimental conditions with *p* ≤ 0.05 considered statistically significant. Statistical significance levels are denoted as follows: ^∗^*p* ≤ 0.05; ^∗∗^*p* ≤ 0.01; ^∗∗∗^*p* ≤ 0.001; ^∗∗∗∗^*p* ≤ 0.0001. No statistical methods were used to predetermine sample size. Super exact test was performed to test the significance of the Venn diagram by the *R* package Exact.

## Supplemental information

Document S1. Supplemental experimental procedures, Figures S1–S5, and Tables S3–S5

File S1. Uncropped full-length western blots

Table S1. Interactome data and Gene Ontology

Table S2. Differentially expressed genes (DEGs)_RNA-Seq ZZZ3 KD ESCs vs. SCR control ESCs

Document S2. Article plus supplemental information
