# Supplementary material for: Unraveling the impact of ZZZ3 on the mTOR/ribosome pathway in human embryonic stem cells homeostasis
Source: Stem Cell Reports. 2024 May 2;19(5):729–43. doi: 10.1016/j.stemcr.2024.04.002 (PMC11103890; doi:10.1016/j.stemcr.2024.04.002)
Supplement: Document S1. Supplemental experimental procedures, Figures S1–S5, and Tables S3–S5 [file mmc1.pdf]

Stem Cell Reports, Volume *19*

Supplemental Information

Unraveling the impact of ZZZ3 on the mTOR/ribosome pathway in human embryonic stem cells homeostasis

Michela Lo Conte, Valeria Lucchino, Stefania Scalise, Clara Zannino, Desirèe Valente, Giada Rossignoli, Maria Stella Murfuni, Chiara Cicconetti, Luana Scaramuzzino, Danilo Swann Matassa, Anna Procopio, Graziano Martello, Giovanni Cuda, and Elvira Immacolata Parrotta

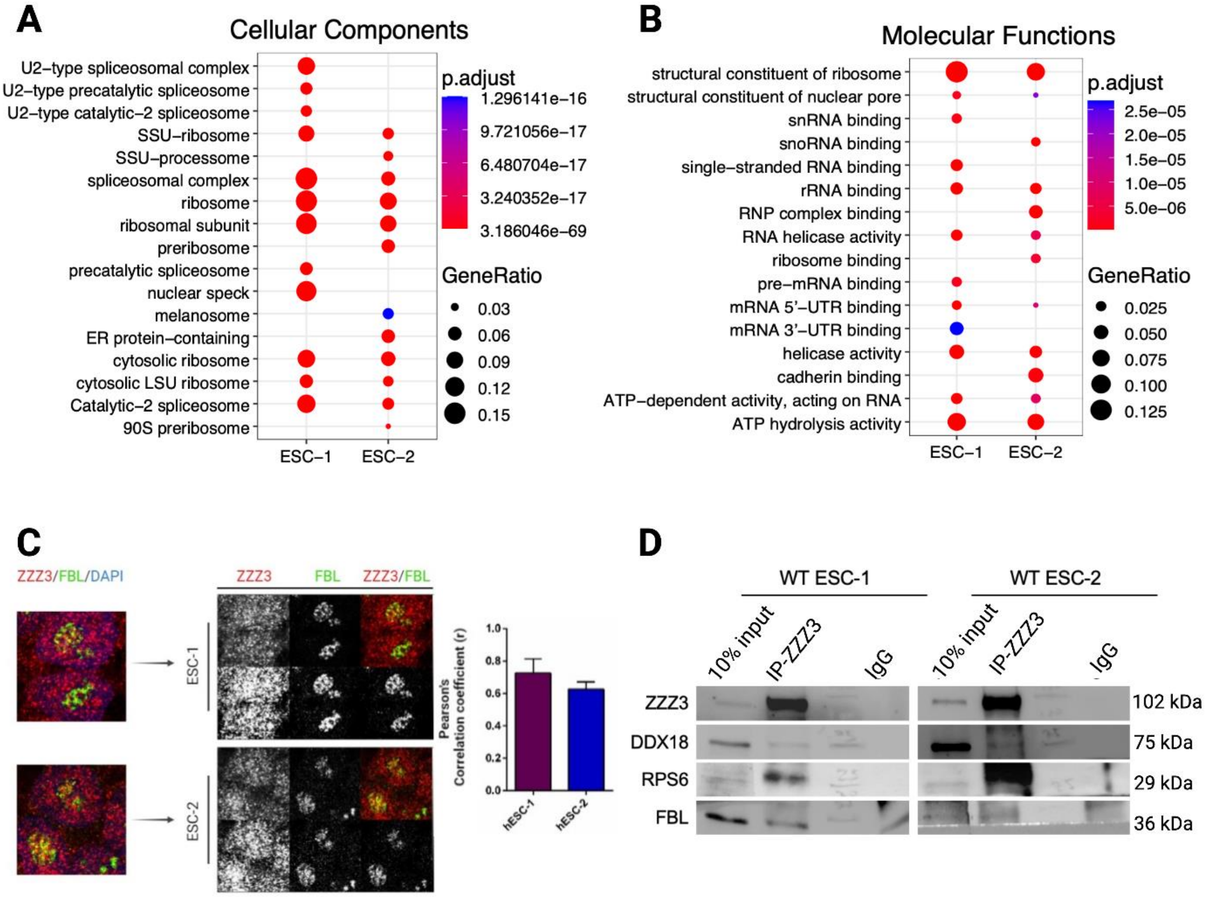
A
Cellular Components
U2−type spliceosomal complex
U2−type precatalytic spliceosome
U2−type catalytic−2 spliceosome
SSU−ribosome
SSU−processome
spliceosomal complex
ribosome
ribosomal subunit
preribosome
precatalytic spliceosome
nuclear speck
melanosome
ER protein−containing
cytosolic ribosome
cytosolic LSU ribosome
Catalytic−2 spliceosome
90S preribosome
ESC−1
ESC−2
p.adjust
1.296141e−16
9.721056e−17
6.480704e−17
3.240352e−17
3.186046e−69
GeneRatio
0.03
0.06
0.09
0.12
0.15
B
Molecular Functions
structural constituent of ribosome
structural constituent of nuclear pore
snRNA binding
snoRNA binding
single−stranded RNA binding
rRNA binding
RNP complex binding
RNA helicase activity
ribosome binding
pre−mRNA binding
mRNA 5'−UTR binding
mRNA 3'−UTR binding
helicase activity
cadherin binding
ATP−dependent activity, acting on RNA
ATP hydrolysis activity
ESC−1
ESC−2
p.adjust
2.5e−05
2.0e−05
1.5e−05
1.0e−05
5.0e−06
GeneRatio
0.025
0.050
0.075
0.100
0.125
C
ZZZ3/FBL/DAPI
ZZZ3
FBL
ZZZ3/FBL
ESC-1
ESC-2
Pearson's Correlation coefficient (r)
1.0
0.8
0.6
0.4
0.2
0.0
hESC-1
hESC-2
D
WT ESC-1
WT ESC-2
10% input
IP-ZZZ3
IgG
10% input
IP-ZZZ3
IgG
ZZZ3
DDX18
RPS6
FBL
102 kDa
75 kDa
29 kDa
36 kDa

Figure S1. ZZZ3 is co-expressed with fibrillarin in the nucleolus and interacts with proteins involved in post-transcriptional processes and ribosome biogenesis. (Related to Fig. 1). A. GO for Cellular Components (**A**) and Molecular Functions (**B**) of differentially expressed genes selected on the basis of the *p*-values (*p*-value < 0.05 corrected by using Benjamini-Hochberg procedure), and fold-change (FC ≥ 2.5). GO analysis was performed in *R* using the Bioconductor package. **C**. Representative immunofluorescence images showing the colocalization of ZZZ3 (red) and Fibrillarin (FBL, green) were captured in wild-type ESC-1 and -2. Utilizing the JACoP BIOP colocalization plugin within the ImageJ software, the degree of colocalization was assessed by calculating the Pearson's correlation coefficient (r) across approximately 40 regions of interest (ROIs), representing the cell areas positive for FBL signal. These ROIs were randomly selected from three distinct stainings of both ESC lines. The intensity-based correlation analysis revealed positive values of the correlation coefficient, indicating the colocalization of ZZZ3 and FBL within the nucleolus of wild-type ESCs. **D**. Immunoprecipitation (IP) with an anti-ZZZ3 antibody followed by Western blot analysis was employed to validate interactome data. Specifically, interactions of ZZZ3 with DDX18, RPS6, and FBL are shown. Full-length uncropped blots are available in Supplementary File S1.

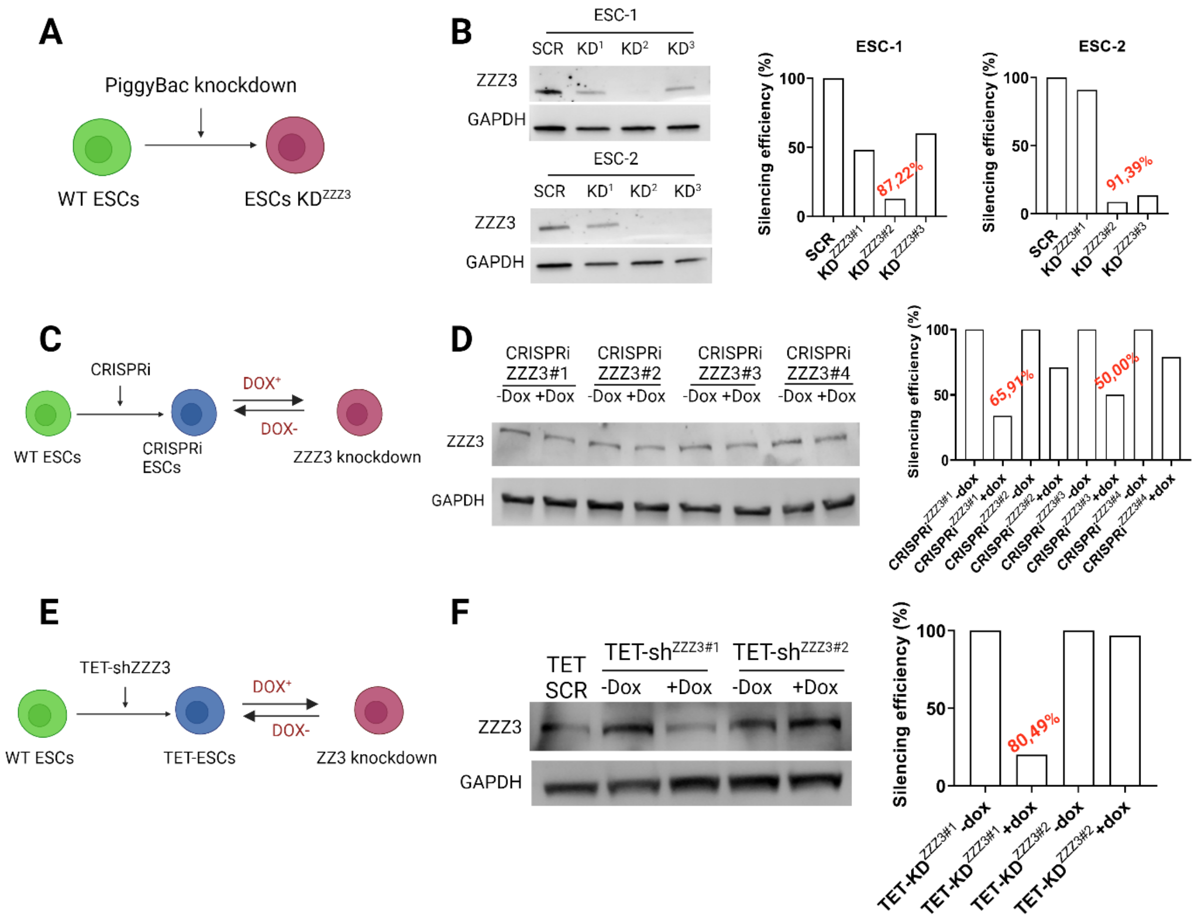
A
PiggyBac knockdown
WT ESCs
ESCs KD ZZZ3
B
ESC-1
SCR KD1 KD2 KD3
ZZZ3
GAPDH
ESC-2
SCR KD1 KD2 KD3
ZZZ3
GAPDH
ESC-1
Silencing efficiency (%)
87,22%
SCR
KD ZZZ3#1
KD ZZZ3#2
KD ZZZ3#3
ESC-2
Silencing efficiency (%)
91,39%
SCR
KD ZZZ3#1
KD ZZZ3#2
KD ZZZ3#3
C
CRISPRi
DOX+
DOX-
WT ESCs
CRISPRi ESCs
ZZZ3 knockdown
D
CRISPRi ZZZ3#1
CRISPRi ZZZ3#2
CRISPRi ZZZ3#3
CRISPRi ZZZ3#4
-Dox +Dox -Dox +Dox -Dox +Dox -Dox +Dox
ZZZ3
GAPDH
Silencing efficiency (%)
65,91%
50,00%
CRISPRi ZZZ3#1 -dox
CRISPRi ZZZ3#1 +dox
CRISPRi ZZZ3#2 -dox
CRISPRi ZZZ3#2 +dox
CRISPRi ZZZ3#3 -dox
CRISPRi ZZZ3#3 +dox
CRISPRi ZZZ3#4 -dox
CRISPRi ZZZ3#4 +dox
E
TET-shZZZ3
DOX+
DOX-
WT ESCs
TET-ESCs
ZZ3 knockdown
F
TET SCR
TET-sh ZZZ3#1
TET-sh ZZZ3#2
-Dox +Dox -Dox +Dox
ZZZ3
GAPDH
Silencing efficiency (%)
80,49%
TET-KD ZZZ3#1 -dox
TET-KD ZZZ3#1 +dox
TET-KD ZZZ3#2 -dox
TET-KD ZZZ3#2 +dox

Figure S2. Knockdown strategies utilized to silence the ZZZ3 gene in two lines of human ESCs. A. Schematic representation of the PiggyBac vector expressing shRNA against the human ZZZ3 gene under the control of the U6 promoter, utilized to establish stable ZZZ3 knockdown in hESCs. **B**. Immunoblot analysis was performed to evaluate the efficiency of ZZZ3 knockdown using three distinct shRNAs, labelled as KD^1, KD^2, and KD^3. Notable, high efficient ZZZ3 knockdown was observed in cells transfected with shZZZ#2 (KD^2), which were subsequently utilized in this study. **C** and **D**. CRISPR interference (CRISPRi) targeting ZZZ3 mRNA and its validation via relative western blot analysis. CRISPRi-ZZZ3#1 and #3 exhibited effective ZZZ3 knockdown. **E** and **F**. TET-inducible shZZZ3 strategy and its validation by immunoblot analysis. The sequences of shRNA and guide RNA (gRNA) are provided in Supplementary Table S3.

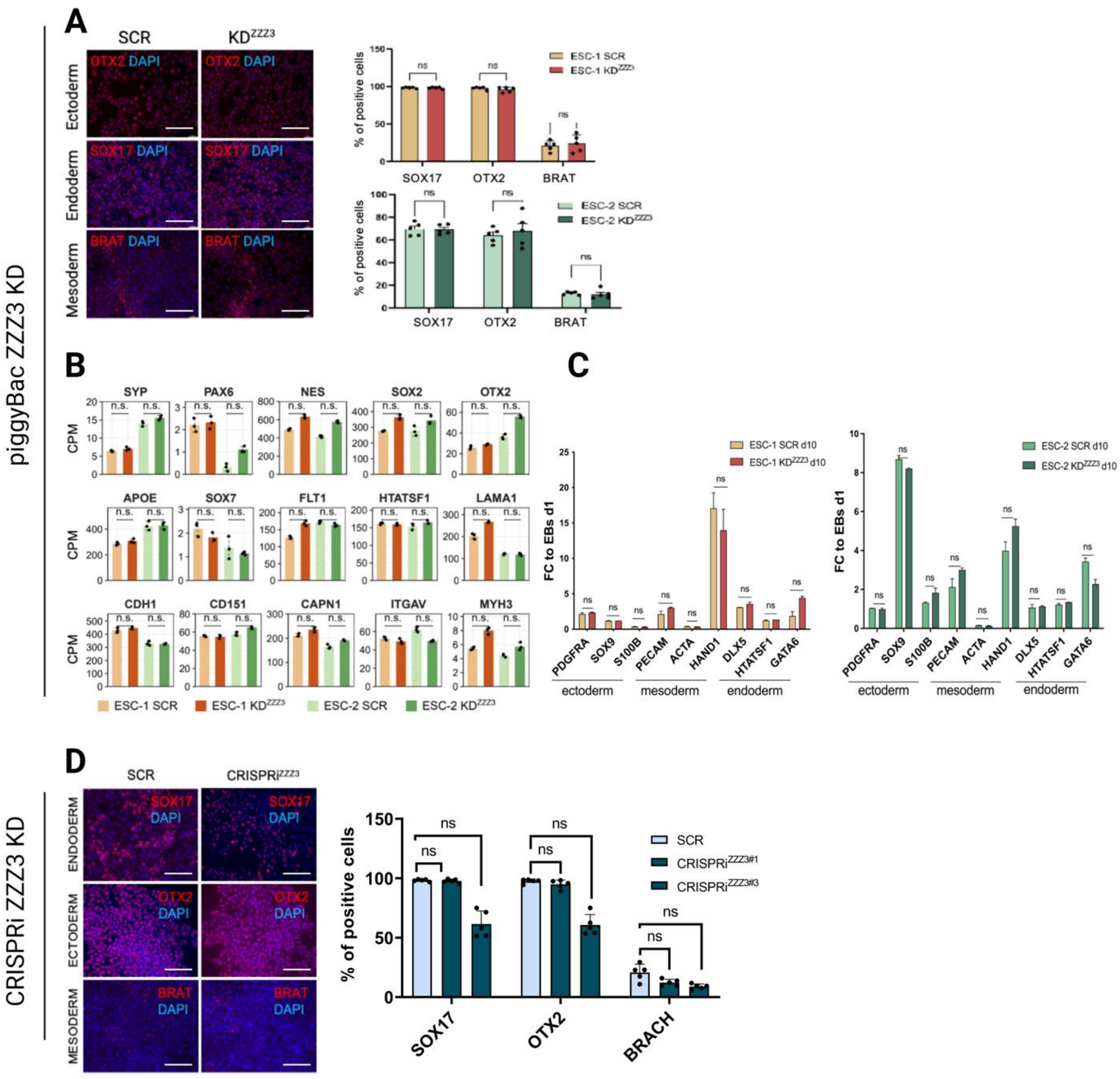
A
SCR
KDZZZ3
Ectoderm
Endoderm
Mesoderm
OTX2 DAPI
SOX17 DAPI
BRAT DAPI
% of positive cells
ESC-1 SCR
ESC-1 KDZZZ3
ESC-2 SCR
ESC-2 KDZZZ3
SOX17
OTX2
BRAT
ns
piggyBac ZZZ3 KD
B
SYP
PAX6
NES
SOX2
OTX2
APOE
SOX7
FLT1
HTATSF1
LAMA1
CDH1
CD151
CAPN1
ITGAV
MYH3
CPM
n.s.
ESC-1 SCR
ESC-1 KDZZZ3
ESC-2 SCR
ESC-2 KDZZZ3
C
ESC-1 SCR d10
ESC-1 KDZZZ3 d10
ESC-2 SCR d10
ESC-2 KDZZZ3 d10
FC to EBs d1
PDGFRA
SOX9
S100B
PECAM
ACTA
HAND1
DLX5
HTATSF1
GATA6
ectoderm
mesoderm
endoderm
D
SCR
CRISPRiZZZ3
CRISPRi ZZZ3 KD
ENDODERM
ECTODERM
MESODERM
SOX17 DAPI
OTX2 DAPI
BRAT DAPI
% of positive cells
SCR
CRISPRiZZZ3#1
CRISPRiZZZ3#3
SOX17
OTX2
BRACH
ns

Figure S3. The expression of the three germ layers is not impaired upon ZZZ3 knockdown. (Related to Fig. 2). A. Immunofluorescence staining was performed to visualize the expression of specific markers indicative of ectoderm, endoderm, and mesoderm differentiation in the SCR control and ZZZ3 KD hESCs. Antibodies targeting key markers - such as OTX2 (ectoderm), BraT (mesoderm), and SOX17 (endoderm) - were used. Nuclei were counterstained with DAPI. Scale bar = 50 μm. Quantification of immunofluorescence images based on the percentage of positive cells was performed using ImageJ software. Data are presented as mean ± standard error of the mean (SEM) from n = 3 independent experiments. The difference observed between the SCR control and ZZZ3 KD hESCs was not statistically significant (ns) (graph on the right). **B**. Barplots show the gene expression levels of a panel of three germ layer regulators in SCR and ZZZ3 KD hESCs as determined by RNA-seq analysis. Mean expression levels ± SEM from three independent experiments is represented by bars, with individual data points overlaid as dots. Statistical analysis was performed using ANOVA, indicating non-significant differences (ns) between the experimental groups. **C**. Quantitative polymerase chain reaction (qPCR) was employed to measure the mRNA expression levels of the three germ layer markers on SCR and ZZZ3 KD EBs at day 10 of differentiation. Non-significant differences (ns) were detected between the experimental groups. **D.** Immunofluorescence staining of specific markers indicative of ectoderm (OTX2), endoderm (SOX17), and mesoderm (BraT) differentiation in the SCR control and ZZZ3 KD hESCs generated via CRISPRi system. Nuclei were counterstained with DAPI. Scale bar = 50 μm. Quantification of immunofluorescence images based on the percentage of positive cells was performed using ImageJ software. Data are presented as mean ± standard error of the mean (SEM) from n = 3 independent experiments. The difference observed between the SCR control and CRISPRi-ZZZ3sh hESCs was not statistically significant (ns) (graph on the right).

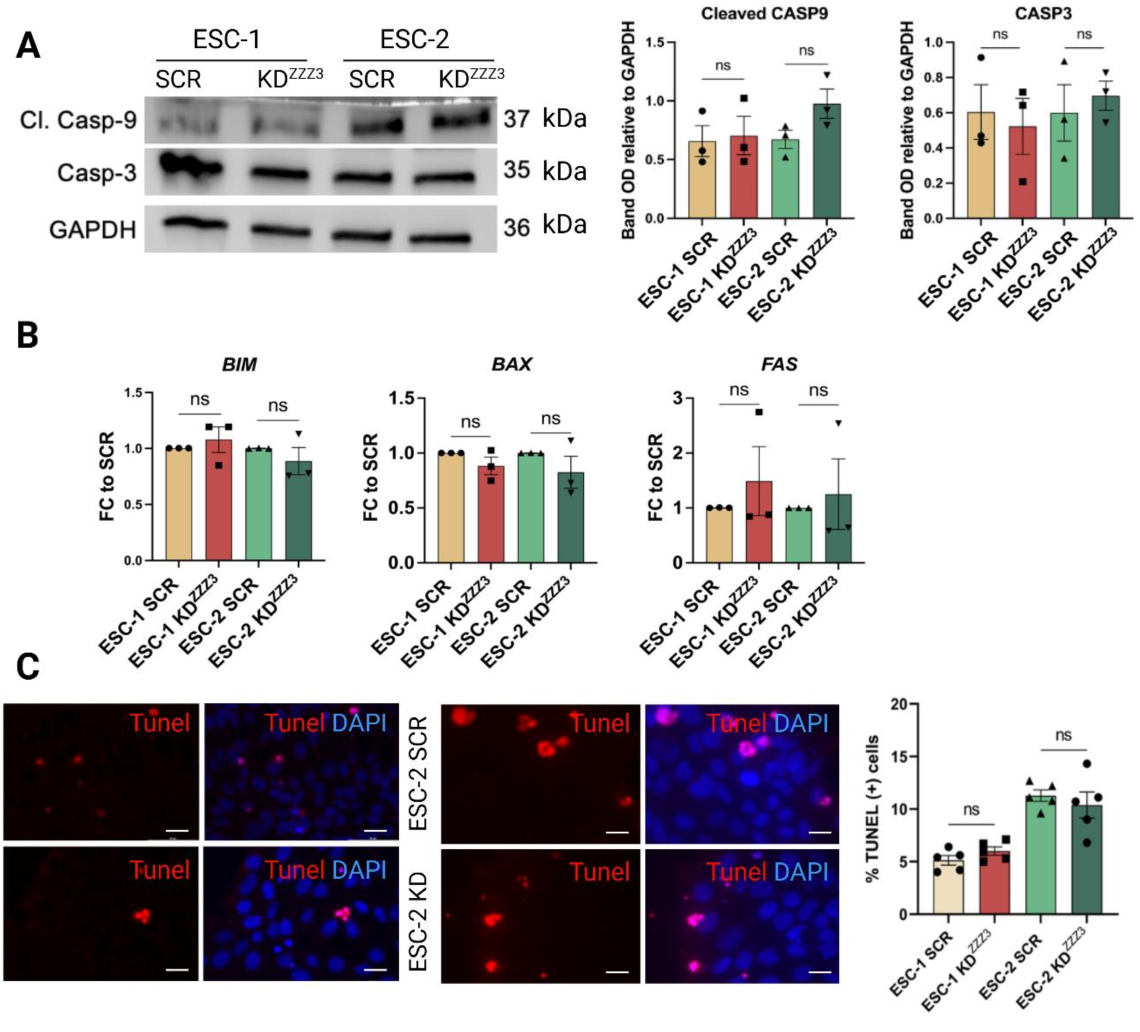
A
ESC-1
ESC-2
SCR
KD^{ZZZ3}
Cl. Casp-9
Casp-3
GAPDH
37 kDa
35 kDa
36 kDa
Cleaved CASP9
CASP3
Band OD relative to GAPDH
ns
ESC-1 SCR
ESC-1 KD^{ZZZ3}
ESC-2 SCR
ESC-2 KD^{ZZZ3}
B
BIM
BAX
FAS
FC to SCR
C
ESC-1 SCR
ESC-1 KD
ESC-2 SCR
ESC-2 KD
Tunel
Tunel DAPI
% TUNEL (+) cells

Figure S4. The decrease in cell proliferation is not attributed to apoptosis. (Related to Figures 3 and 5). A. Western blot analysis was conducted to assess the protein levels of cleaved caspase 9 and caspase 3 in SCR control and ZZZ3 KD hESCs (left); quantification of protein expression levels was performed using optical density (OD) measurement for each immunoblot shown. Data are presented as mean ± standard error of the mean (SEM) from n = 3 independent experiments. Significance was calculated vs. relative SCR ESCs using *t*-test. The difference observed between the groups was not statistically significant (ns) (right). **B**. Quantitative polymerase chain reaction (qPCR) was employed to measure the mRNA expression levels of *BIM*, *BAX*, and *FAS* genes in ZZZ3 KD vs. SCR control. Data are shown as mean ± SEM of three independent experiments and *t*-test was calculated *vs.* SCR cells (ns = not significant). **C**. Immunofluorescence staining for TUNEL was performed to detect DNA fragmentation indicative of apoptosis. Relative quantification was carried out to assess the extent of TUNEL-positive cells or fluorescence intensity. Scale bar: 50 µm. Quantification of immunofluorescence signals was performed using ImageJ software. Data are presented as mean ± standard error of the mean (SEM) from n = 3 independent experiments (at least 200 nuclei were analysed). Significance was calculated vs. relative SCR ESCs using *t*-test, ns = not significant.

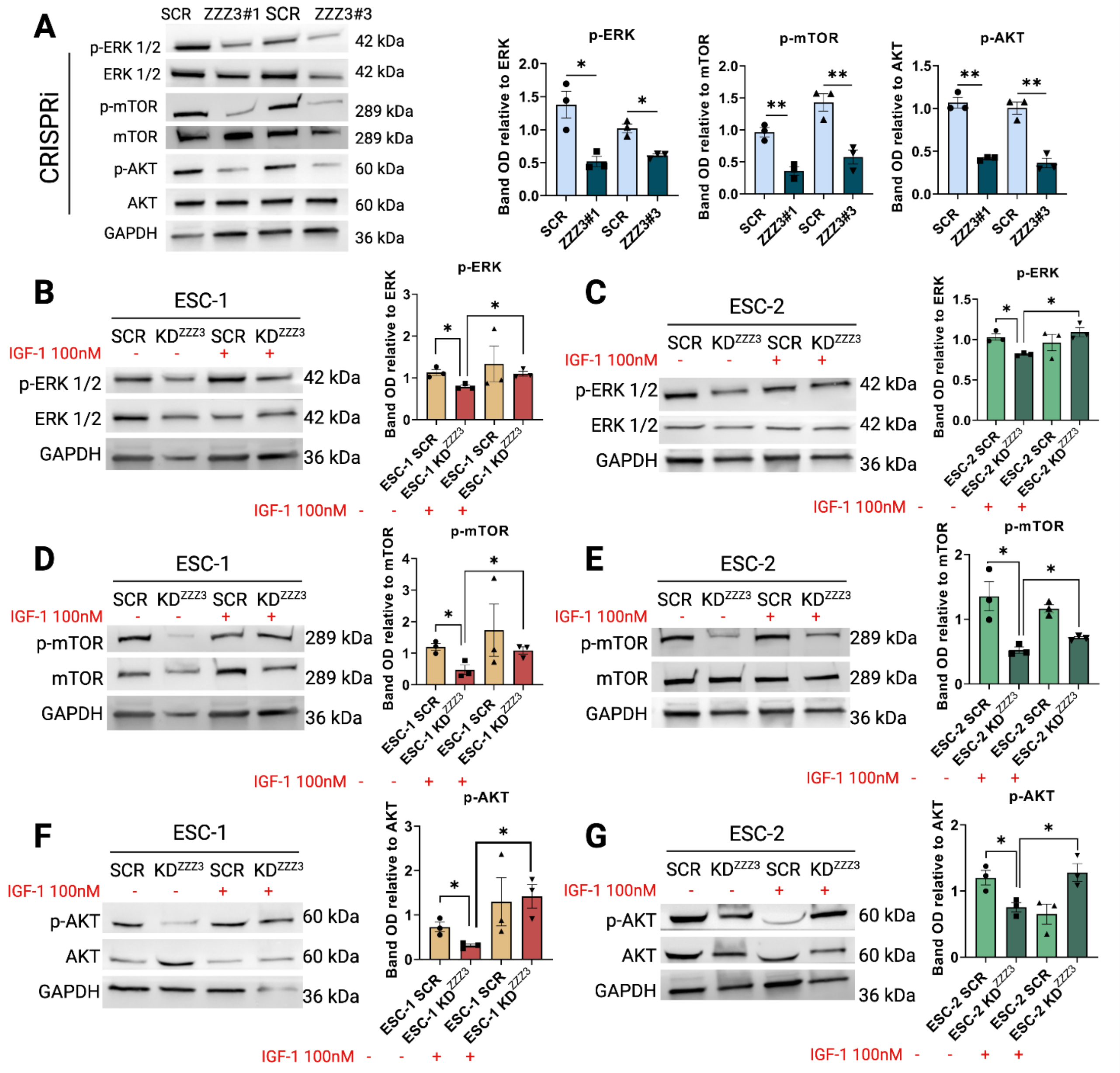
A
SCR ZZZ3#1 SCR ZZZ3#3
CRISPRi
p-ERK 1/2
ERK 1/2
p-mTOR
mTOR
p-AKT
AKT
GAPDH
42 kDa
42 kDa
289 kDa
289 kDa
60 kDa
60 kDa
36 kDa
p-ERK
Band OD relative to ERK
p-mTOR
Band OD relative to mTOR
p-AKT
Band OD relative to AKT
SCR
ZZZ3#1
SCR
ZZZ3#3
B
ESC-1
SCR KD^{ZZZ3} SCR KD^{ZZZ3}
IGF-1 100nM - - + +
p-ERK 1/2
ERK 1/2
GAPDH
Band OD relative to ERK
ESC-1 SCR
ESC-1 KD^{ZZZ3}
C
ESC-2
Band OD relative to ERK
ESC-2 SCR
ESC-2 KD^{ZZZ3}
D
ESC-1
p-mTOR
mTOR
Band OD relative to mTOR
E
ESC-2
F
ESC-1
p-AKT
AKT
Band OD relative to AKT
G
ESC-2

Figure S5. IGF-1 rescues dysregulated PI3K/Akt/mTOR signaling pathways in ZZZ3 knockdown hESCs. (Related to Fig. 6). A. Western blot analysis was used to confirm the reduction of p-ERK 1/2, p-mTOR and p-AKT expression upon knockdown of ZZZ3 using SCR control and ZZZ3 KD hESCs generated via CRISPRi system. Quantification of protein expression levels was performed using optical density (OD) measurement. Data are presented as mean ± SEM from n = 3 independent experiments. Significance was calculated vs. relative SCR ESCs using t-test, * $p \leq 0.05$, ** $p \leq 0.01$. **B-F.** Immunoblot analysis was performed as readout of the rescue of key components of the PI3K/Akt/mTOR Signaling Pathways in ZZZ3 KD hESCs (ESC-1 and ESC-2 cell lines). **B.** and **C.** Representative immunoblots showing the protein expression levels of total ERK and phospho-ERK (Thr202/Tyr204) in ZZZ3 KD ESC-1 and ESC-2 treated and untreated with IGF-1 (100 mM, 30 minutes); **D.** and **E.** Representative immunoblots showing the protein expression levels of total mTOR and phospho-mTOR (Ser2448) in ZZZ3 KD hESCs treated and untreated with IGF-1; **F.** and **G**. Representative immunoblots showing the protein expression levels of total Akt and phospho-Akt (Ser473) in ZZZ3 KD hESCs treated and untreated with IGF-1. Quantification of immunoblot signals reveals a significant restoration of PI3K, Akt, and mTOR phosphorylation levels upon IGF-1 treatment in ZZZ3 knockdown hESCs compared to untreated knockdown cells. Data are presented as mean ± standard error of the mean (SEM) from n = 3 independent experiments. Significance was calculated vs. relative SCR ESCs cells using *t*-test, * $p \leq 0.05$.

SUPPLEMENTARY TABLES

Table S3. Sequences used to induce ZZZ3 knockdown.

- **PiggyBac:** pPB[shRNA]-Hygro-U6>_shRNA

- **TET-KD:** pPB [TetOn]-TRE>EGFP: {hZZZ3[miR30-shRNA#1]} -rev (CAG>tTS:T2A:rtTA:P2A:Hygro)

- **CRISPRi - sgRNA delivery and transactivation plasmid:** pPB-Ins-U6p-sgRNAentry-EF1Ap-TetOn3G-IRES-Neo (the necessary tails in the ends for the cloning are highlighted).

**shRNA Knockdown PiggyBac**	Target Sequence
hZZZ3[shRNA#1]	TCACCAATTAGAACCTATTTA
hZZZ3[shRNA#2]	TCAAAGAACTTGGTCATAAAT
hZZZ3[shRNA#3]	GACGACAGCACCCTCTTAATA
**TET-inducible shRNA**	Sequence
shRNA#1	AGCAGAACACCAAACTTATATA
shRNA#2	CAAAGAACTTGGTCATAAAT
**CRISPRi - gRNA**	Sequence
ZZZ3_sgRNA #1	Forward (5’ → 3’) **CACC**ACAGACGATTGCTGTACTCG<br>Reverse (5’ → 3’) **AAAC**CGAGTACAGCAATCGTCTGT
ZZZ3_sgRNA #2	Forward (5’ → 3’) **CACC**TGGGTAAGCCCTAGGAAAAG<br>Reverse (5’ → 3’) **AAAC**CTTTTCCTAGGGCTTACCCA
ZZZ3_sgRNA #3	Forward (5’ → 3’) **CACC**GAGCCTGTGCCAATTCAGAA<br>Reverse (5’ → 3’) **AAAC**TTCTGAATTGGCACAGGCTC
ZZZ3_sgRNA #4	Forward (5’ → 3’) **CACC**AAGACCTTGAAAGTTTAGGC<br>Reverse (5’ → 3’) **AAAC**GCCTAAACTTTCAAGGTCTT

Table S4. List of antibodies used for Western Blot (WB) and Immunofluorescence (IF) analyses.

Antibody	Host species	Dilution	Cat.No	Company	Application
ZZZ3	Rabbit	1:2000	ab118800	Abcam	WB
Nanog	Goat	1:500	AF1997	R&D System	WB
Oct4	Rabbit	1:1000	bs-1111R	Bioss Antibodies	WB
pERK1/2 (Thr202/Tyr204)	Rabbit	1:1000	9101	Cell Signaling Technology	WB
ERK1/2	Mouse	1:1000	9107	Cell Signaling Technology	WB
p53	Mouse	1;500	sc-393031	Santa Cruz Biotechnology	WB
RPL19a	Mouse	1:1000	sc-100830	Santa Cruz Biotechnology	WB
Phospho-RPS6 (S235)	Rabbit	1:1000	ab227005	Abcam	WB
Ribosomal Protein S6	Mouse	1:2000	sc-74459	Santa Cruz Biotechnology	WB
Phospho-Akt (Ser473) (D9E)	Rabbit	1:2000	4060S	Cell Signaling Technology	WB
Akt	Rabbit	1:1000	9272S	Cell Signaling Technology	WB
Phospho-mTOR (Ser2448)	Rabbit	1:1000	5536S	Cell Signaling Technology	WB
mTOR	Rabbit	1:1000	2983S	Cell Signaling Technology	WB
Phospho-4E-BP1 (Thr37/46)	Rabbit	1:1000	2855	Cell Signaling Technology	WB
4E-BP1	Rabbit	1:1000	9452	Cell Signaling Technology	WB
Phospho-eIF4E(Ser209)	Rabbit	1:1000	9741	Cell Signaling Technology	WB
eIF4E	Rabbit	1:1000	9742	Cell Signaling Technology	WB
Phospho-p70 S6 Kinase (Thr421/Ser424)	Rabbit	1:1000	9204	Cell Signaling Technology	WB
p70 S6 Kinase	Rabbit	1:1000	2708	Cell Signaling Technology	WB
Cleaved Caspase-9	Rabbit	1:1000	9505	Cell Signaling Technology	WB
Caspase-3 (3G2)	Mouse	1:1000	9668	Cell Signaling Technology	WB

Antibody	Host species	Dilution	Cat.No	Company	Application
c-myc	Rabbit	1:1000	5605s	Cell Signaling Technology	WB
E2F4	Rabbit	1:1000	40291	Cell Signaling Technology	WB
Fibrillarin	Mouse	1:2000	ab4566	Abcam	WB
DDX18	Rabbit	1:2000	A300-636-A	Bethyl	WB
GAPDH	Rabbit	1:1000	bs10900R	Bioss Antibodies	WB
Actin	Goat	1:500	sc1616	Santa Cruz Biotechnology	WB
Peroxidase AffiniPure Donkey Anti-Rabbit IgG (H+L)		1:10000	711-035-152	Jackson Immuno Reaserch	WB
Peroxidase AffiniPure Sheep Anti-Mouse IgG (H+L)		1:10000	515-035-062	Jackson Immuno Reaserch	WB
Peroxidase AffiniPure Rabbit AntiGoat IgG (H+L)		1:5000	305-035-045	Jackson Immuno Reaserch	WB
ZZZ3	Rabbit	1:100	Pa5-84224	Invitrogen	IF
Fibrillarin	Mouse	1:500	ab4566	Abcam	IF
Nanog	Goat	1:200	AF1997	R&D System	IF
Oct4	Mouse	1:200	75463	Cell Signaling Technology	IF
Ki67	Rabbit	1:400	9129S	Cell Signaling Technology	IF
p21	Rabbit	1:400	2947S	Cell Signaling Technology	IF
Anti-Rabbit IgG Alexa Fluor 594		1:500	A-11012	Invitrogen	IF
Anti-Rabbit IgG Alexa Fluor 488		1:500	A-11008	Invitrogen	IF
Anti-Goat IgG Alexa Fluor 594		1:500	A-11058	Invitrogen	IF
Anti-Mouse IgG Alexa Fluor 488		1:2000	A-11001	Invitrogen	IF

Table S5. Primers used for quantitative PCR analysis.

Gene	Primer sequence
GAPDH	For_ TCCTCTGACTTCAACAGCGA
	Rev_ GGGTCTTACTCCTTGGAGGC
OCT4	For_ GGAGGAAGCTGACAACAATGAA
	Rev_ GGCCTGCACGAGGGTTT
NANOG	For_ TGCAAGAACTCTCCAACATCCT
	Rev_ ATTGCTATTCTTCGGCCAGTT
SOX2	For_ ATGCACCGCTACGACGTGA
	Rev_ CTTTTGCACCCCTCCCATTT
PDGFRA	For_ TAATGACTCACCTGGGGCCA
	Rev_ CTTTGGCTTCTCTGGGTGGT
SOX9	For_ AGCTCTGGAGACTTCTGAACGAGA
	Rev_ CGTTCTTCACCGACTTCCTCCGC
S100B	For_ CATCGACGTTTTCCACCAAT
	Rev_ GAAGTCACATTCGCCGTCTC
PECAM	For_ ATGCCGTGGAAAGCAGATAC
	Rev_ CTGTTCTTCTCGGAACATGGA
ACTA	For_ TCTGGCACCACACCTTCTACAATG
	Rev_ AGCACAGCCTGGATAGCAACG
HAND1	For_ CCAGCTACATCGCCTACCTG
	Rev_ CCGGTGCGTCCTTTAATCCT
DLX5	For_ TTCAGAAGACTCAGTACCTCGC
	Rev_ GAGTTACACGCCATTGGGTC
HTATSF1	For_ ACGATGGCGCATCTAGTTCT
	Rev_ ATCTGGAGGCAAACCAGACA
GATA6	For_ AGCGACTCCAGAGCCTTTC
	Rev_ATGCGAAGCGTAGGAACTGA
BIM	For_ GCTGTCTCGATCCTCCAGTG
	Rev_ TCCAATACGCCGCAACTCTT
BAX	For_ GGACGAACTGGACAGTAACATGG
	Rev_ GCAAAGTAGAAAAGGGCGACAAC

FAS	For_ GGAGTACACAGACAAAGCCCA
	Rev_ TTTGGTGCAAGGGTCACAGT

EXPERIMENTAL PROCEDURES

Cell culture. The human ES cell-lines (WA17 and RUES2; WiCell Research Institute, Madison, WI) were maintained in mTeSR1 Plus medium (STEMCELL Technologies, Vancouver, Canada) on plates coated with Matrigel (BD Biosciences, San Diego, CA) in a humidified incubator at 37°C and 5% CO_2. Medium was replaced every two days and cells were split every 5 to 6 days using Gentle Dissociation reagent (STEMCELL Technologies, Vancouver, Canada). hES cells were routinely tested for Mycoplasma using the Mycoplasma PCR Detection kit (Applied Biological Materials, Richmond, Canada).

Cell proliferation assay. Human ESCs were plated onto Matrigel-coated 12-well plates at a density of $3x10^4$ cells per well and cultured in mTeSR1 Plus medium for specified durations (24h, 48h, 72h, and 96h). Subsequently, cells were detached using Accutase (Thermo Fisher Scientific) and counted using a cell counting chamber. Each experiment was repeated independently three times.

MTT assay. 5,000 hES cells per well were seeded in triplicate onto a 96-well plate matrigel-coated. At 80% confluence, cells were treated with 0.5 mg/ml of 3-[4,5-dimethylthiazol-2-yl] -2,5-diphenyl-tetrazolium bromide (MTT) for 2 hours. Subsequently, the MTT solution was replaced with 2-propanol (Sigma-Aldrich) and agitated for 10 minutes. Absorbance was then measured at 570 nm using a Varioskan LUX plate reader (Thermo Fisher Scientific).

Synchronization and cell cycle profile analysis. SCR control and ZZZ3 knockdown ESCs were synchronized by treating them with nocodazole (100 ng/ml, Thermo Fisher Scientific) for 12 hours, causing their accumulation in the G2/M phase. Cell cycle analysis was conducted by harvesting cells immediately after nocodazole treatment (T0) and 8 hours after nocodazole withdrawal (T1). For cell profile analysis, cells were collected by centrifugation, washed with ice-cold PBS, and fixed with 70% ethanol overnight at 4°C. Following fixation, the cells were washed with PBS, resuspended in PBS containing propidium iodide (50 µg/ml, Thermo Fisher Scientific), RNAsi (100 µg/ml, Thermo Fisher Scientific), and NP40 (0.01%, Santa

Cruz Biotechnology), and incubated for 1 hour in darkness. ZZZ3 knockdown hESCs generated with piggyBac encoding for doxycycline-inducible ZZZ3 shRNA (DoxKD) were utilized to rescue the proliferation defect (Dox^+/Dox^-). Flow cytometry analysis was performed on the BD LSRFortessa x-20 Flow Cytometer, and data was processed using FlowJo software.

TUNEL assay. For *in vitro* apoptosis detection, ESCs were initially seeded into matrigel-coated 8-well chamber slides. The cells were then fixed using 3.7% (vol/vol) formaldehyde (Sigma-Aldrich) and processed with the Click-iT™ Plus TUNEL Assay Kit AlexaFluor 594 (Thermo Fisher Scientific) following the manufacturer's instructions. After completing the assay, the cells were mounted with Dako Fluorescent Mounting Medium (Agilent), and images were captured using Leica microscopy systems (DMi8) equipped with Leica LAS X software (version 3.7.4.23463). TUNEL-positive cells were manually quantified using ImageJ software.

Immunofluorescence staining. Immunofluorescence analysis was performed on Matrigel-coated glass coverslip in wells. Cells were fixed in 3.7% (vol/vol) formaldehyde for 15 minutes at room temperature, washed in PBS, permeabilized for 1h at RT in PBS + 0.3% Triton X-100 (Sigma-Aldrich) (PBST), and blocked for 1h at RT in PBST containing 10% of fetal bovine serum (FBS) (Thermo Fisher Scientific). Cells were subjected to immunostaining overnight at 4°C with primary antibodies (Table S4) diluted in blocking solution. After washing with PBS, cells were incubated with Alexa Fluor 488- or 594- conjugated secondary antibodies (Alexa, Life Technologies) for 1h at RT. Nuclei were stained with DAPI (4′,6-diamidino-2-phenylindole) (Thermo Fisher Scientific). Finally, cells were mounted with DAKO Fluorescent Mounting Medium (Agilent), and images were acquired using Leica microscopy systems (DMi8, Thunder DMi8, and Stellaris 5 confocal) and Leica LAS X software (v.3.7.4.23463). All immunostainings' analyses were performed using ImageJ software. A list of antibodies used for immunostaining is provided in (Supplementary Table S4).

Western blot analysis. Cells were washed with cold PBS and then scraped from the plate using RIPA buffer (150mM Sodium Chloride, 1% Triton x-100, 0.5% sodium deoxycholate, 0.1% SDS (sodium dodecyl sulfate), 50mM Tris hydrochloride, pH 8.0), supplemented with Halt™ Protease Inhibitor and Halt™ Phosphatase Inhibitor Cocktails (Thermo Fisher Scientific). The protein content was determined using Bradford (Bio-Rad) protein assay. Equal amounts of proteins (30-50 µg) were denatured in Laemmli sample buffer at 95°C for 5 minutes, separated on 4-20% Mini-PROTEAN TGX precast gels (Bio-Rad), and transferred onto nitrocellulose membranes (Bio-Rad) using a Trans-Blot® Turbo™ Transfer System (Bio-Rad). Blots were blocked in 5% non-fat milk for 1 h at room temperature and subsequently subjected to overnight primary antibody incubation at 4°C, followed by two quick rinses and three washes for 5 min in PBST (PBS + 0.1% Tween-20). Secondary antibody incubation was performed for 1h at RT. Clarity™ Western ECL Blotting Substrates (Bio-Rad) was used to detect the HRP signal and the western blot images were collected using the Alliance™ Q9-Atom (Uvitec). For rescuing the PI3K-AKT-mTOR defect, both SCR and ZZZ3 knockdown ESCs were treated with 100 nM of insulin-like growth factor (IGF-1) (Sigma-Adrich) for 30 minutes. Uncropped western blots images are shown in File S1. The details regarding specific antibodies used can be found in Table S4.

RNA extraction, reverse transcription, and quantitative real-time PCR. Total RNA was extracted using TRIzol Reagent (Thermo Fisher Scientific) following manufacturer's instructions. Reverse transcription was performed using High-Capacity cDNA Reverse Transcription Kit (Thermo Fisher Scientific). Quantitative PCR analyses were performed in real time using a QuantStudio™ 7 Pro Real Time PCR system (Applied Biosystem) and SensiFAST SYBR Hi-ROX kit (Meridian Bioscience). Gene expressions were calculated following normalization to *GAPDH* (Glyceraldehyde 3-phosphate dehydrogenase) levels using the comparative Ct (cycle threshold) method. Statistical differences were calculated using two-tailed *t*-test or multiple unpaired *t*-test with Welch correction, with a significance of * $p \leq 0.05$, ** $p \leq 0.01$, and *** $p \leq 0.001$. Data are presented as mean ± SEM from three independent experiments. The primer sequences utilized in the qRT-PCR analysis are provided in the Supplementary Table S5.

Nucleolar staining. Nucleoli were stained using the NUCLEOLAR-ID® Green Detection Kit (ENZO) following manufacturer's instructions. The hESCs were grown on coverslips placed inside a Petri dish filled with the mTeSR1 culture medium. At 80% confluence the media was removed and a sufficient volume of NUCLEOLAR-ID® Green Detection Reagent was dispensed onto the monolayer of cells in darkness. Cells were incubated for 15-30 minutes at 37°C. After the incubation period, cells were washed with 100 µL of 1X Assay Buffer. Excess buffer was removed and the stained cells were analyzed using a Stellaris5 confocal microscopy (Leica Microsystems). For imaging the nucleolus, a standard FITC filter set was utilized. Hoechst 34580 dye (Invitrogen) was used to label nuclear DNA.

Polysome profile. Before harvesting, cells were washed with ice-cold PBS supplemented with 100 µg/mL cycloheximide and resuspended in 1 mL lysis buffer (10 mM Tris-HCl pH7.4, 100 mM KCl, 10 mM MgCl2, 1% Triton-X 100, 1 mM DTT, 10 U/mL RNAseOUT (Invitrogen), 100 µg/mL of cycloheximide), and scraped. After 5 min of incubation on ice, cell lysate was centrifuged for 10 min at 14,000 rpm at 4°C. The supernatant was collected and protein content was determined by Bradford analysis (Bio-Rad). Equal protein amounts (4.2 mg) were loaded onto a 10-60% sucrose gradient obtained by adding 6 mL of 10% sucrose over a layer of 6 mL of 60% sucrose prepared in lysis buffer without Triton and containing 0.5 mM DTT, in a 12-mL tube (Polyallomer; Beckman Coulter). Gradients were prepared using a gradient maker (Gradient Master, Biocomp). Polysomes were separated by centrifugation at 37,000 rpm for 2.5 hours using a Beckman SW41 rotor. Twelve fractions of 920 µL were collected while polysomes were monitored by following the absorbance at 254 nm. Total protein was retrieved by 100% ethanol (EtOH) precipitation performed overnight, washed twice with 70% EtOH and analyzed by SDS-PAGE followed by Western blot.

DDA data analysis. Raw files were processed in MaxQuant software (version 2.0.1.0) using the Andromeda search engine. The MS/MS spectra were searched against a human proteome database (downloaded in March 2016 and containing 42.013 sequences). For label free quantification (LFQ) analysis the following settings were used: Carbamidomethylation of cysteines as static modification, and oxidation of methionine and protein N-terminal acetylation as variable modifications. High confidence and unique peptides (minimum 1 peptide

per protein group) were used for protein identification. Further parameters were set as follows: first and main search peptide tolerance, respectively 20 ppm and 4.5 ppm; isotope and centroid match tolerance, respectively 2 and 8 ppm; maximum number of missed cleavages, 2. Match between runs (MBR) option was activated, with match-time window set at 0.7 min and initial alignment window at 20 min. Only unique peptides were selected for quantification with a minimum ratio count of 1. For statistical analysis of MaxQuant output, the Perseus software (version 2.0.6.0) was used as follows: the LFQ intensity of proteins from the MaxQuant analysis were imported and contaminants, reverse identification, and proteins only identified by site were excluded from further data analysis. Data were transformed in logarithmic scale (log2). After filtering (at least three valid LFQ values in at least one group), remaining missing LFQ values were imputed from a normal distribution (width, 0.3; down shift, 1.8). Finally, for all the data sets, paired two-sample t-test was used to assess statistical significance of protein abundances using a 5% permutation-based FDR adjustment. Based on statistical measures described, we detected 525 interacting proteins associated with ZZZ3 in ESC1 and 922 interacting proteins in ESC2. To narrow down potential interactors of ZZZ3, we focused on those exhibiting fold changes of ≥ 2.5 (n = 474 in ESC-1 and n = 744 in ESC-2). The mass spectrometry proteomics data have been deposited to the ProteomeXchange Consortium via the PRIDE (Perez-Riverol et al., 2022) partner repository.

LIST OF OTHER SUPPLEMENTARY FILES:

File S1. Uncropped full-length western blots.

Table S1. Interactome data and Gene Ontology

Table S2. Differentially expressed genes (DEGs)_RNA-Seq ZZZ3 KD ESCs *vs*. SCR control ESCs.
