# Supplementary material for: Unraveling the impact of ZZZ3 on the mTOR/ribosome pathway in human embryonic stem cells homeostasis
Source: Stem Cell Reports. 2024 May 2;19(5):729–43. doi: 10.1016/j.stemcr.2024.04.002 (PMC11103890; doi:10.1016/j.stemcr.2024.04.002)
Supplement: File S1. Uncropped full-length western blots [file mmc2.pdf]

Uncropped western blot images

The bands relative to immunoblot analysis performed in this study and shown in the text are marked by the red rectangle. For all uncropped images the correspondence to the main/supplementary figure is indicated. (NOTE UNCROPPED WB SONO REPLICATI BIOLOGICI)

FIG. 2B

1° REPLICATE

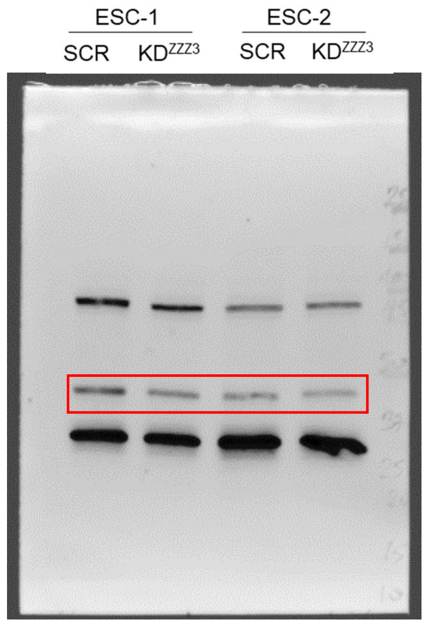

OCT4 MW 42kDa

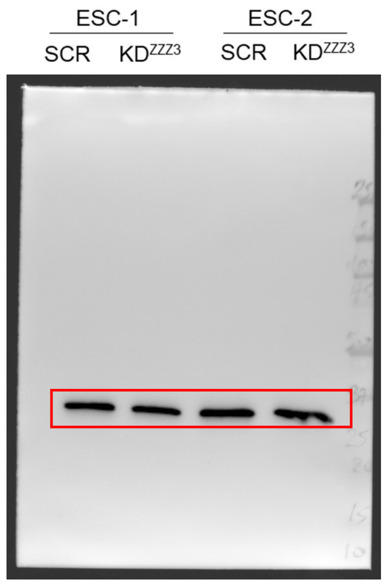

GAPDH MW 36kDa

2° REPLICATE

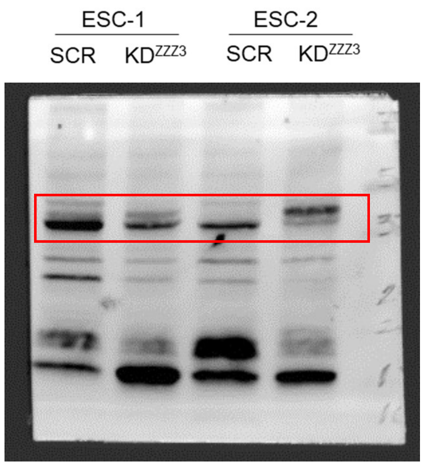

OCT4 MW 42kDa

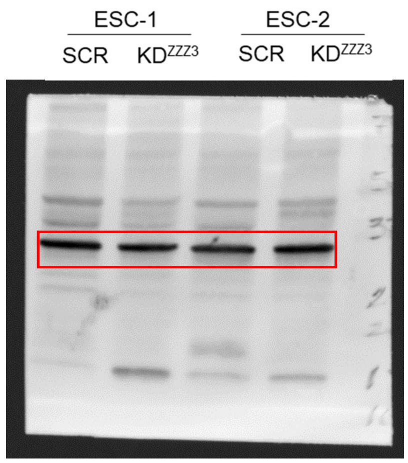

GAPDH MW 36kDa

3° REPLICATE

ESC-1 ESC-2

SCR KD^{ZZZ3} SCR KD^{ZZZ3}

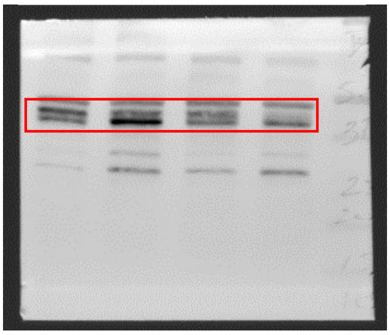

OCT4 MW 42kDa

ESC-1 ESC-2

SCR KD^{ZZZ3} SCR KD^{ZZZ3}

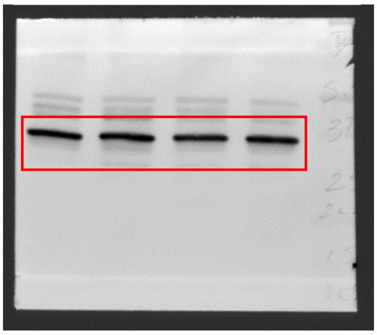

GAPDH MW 36kDa

1° REPLICATE

ESC-1 ESC-2

SCR KD^{ZZZ3} SCR KD^{ZZZ3}

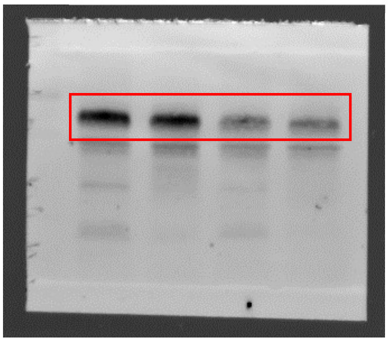

NANOG MW 34kDa

ESC-1 ESC-2

SCR KD^{ZZZ3} SCR KD^{ZZZ3}

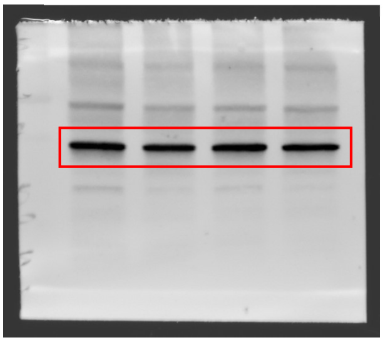

GAPDH MW 36kDa

2° REPLICATE

ESC-1 ESC-2

SCR KD^{ZZZ3} SCR KD^{ZZZ3}

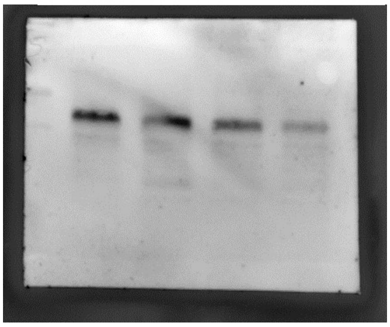

NANOG MW 34kDa

ESC-1 ESC-2

SCR KD^{ZZZ3} SCR KD^{ZZZ3}

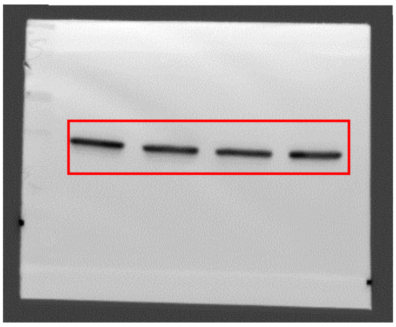

GAPDH MW 36kDa

3° REPLICATE

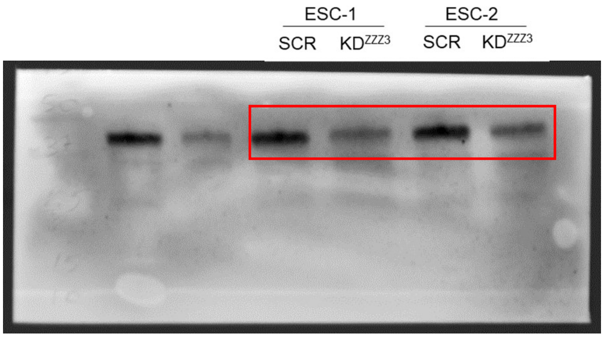

NANOG MW 34kDa

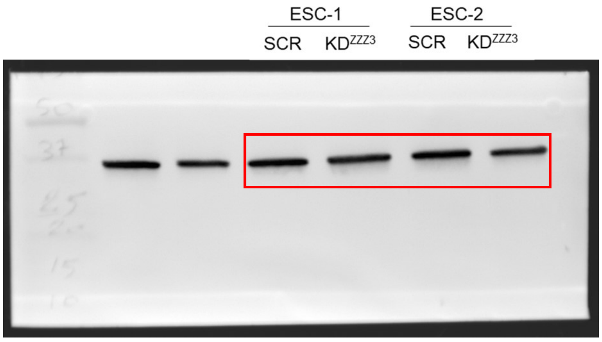

GAPDH MW 36kDa

FIG. 2G

1° REPLICATE

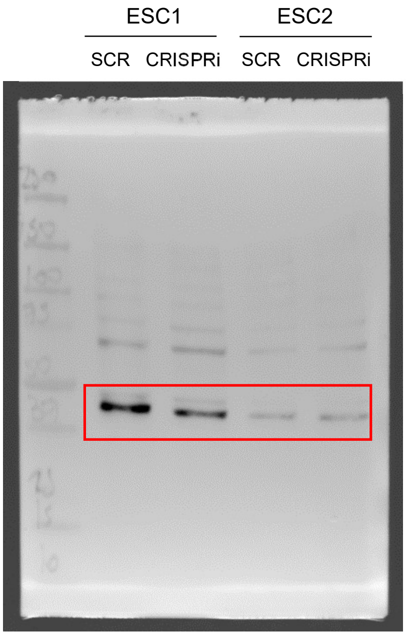

OCT4 MW 42kDa

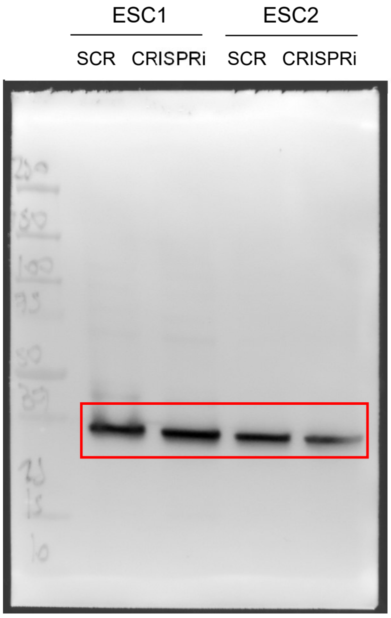

GAPDH MW 36kDa

2° REPLICATE

ESC1 ESC2

SCR CRISPRi SCR CRISPRi

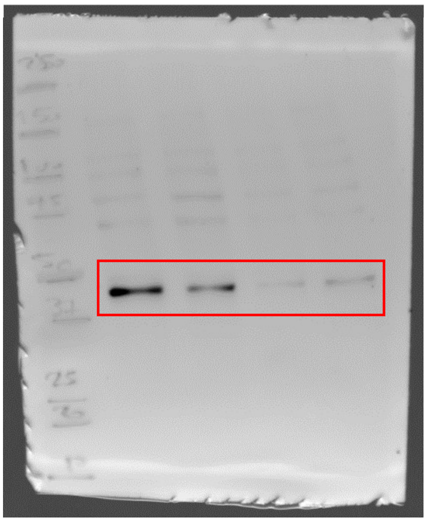

OCT4 MW 42kDa

ESC1 ESC2

SCR CRISPRi SCR CRISPRi

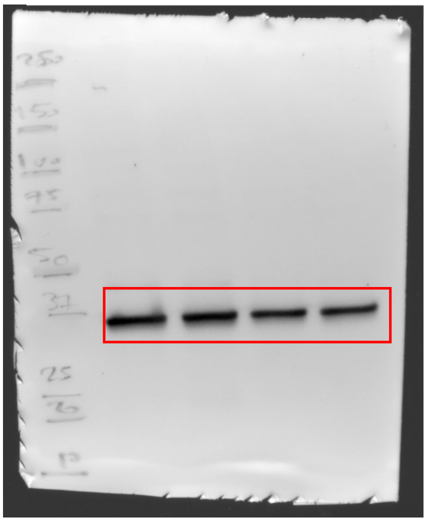

GAPDH MW 36kDa

3° REPLICATE

ESC1 ESC2

SCR CRISPRi SCR CRISPRi

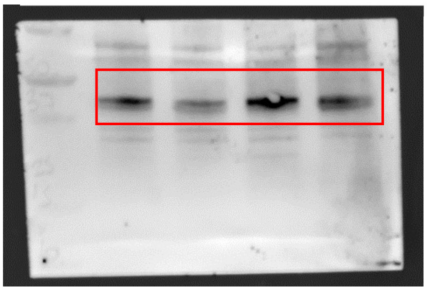

OCT4 MW 42kDa

ESC1 ESC2

SCR CRISPRi SCR CRISPRi

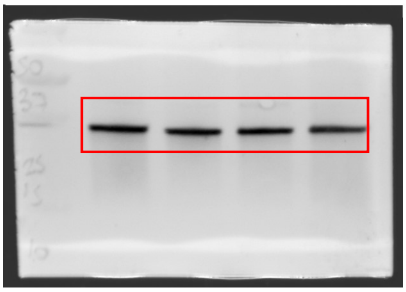

GAPDH MW 36kDa

1° REPLICATE

ESC1 ESC2

SCR CRISPRi SCR CRISPRi

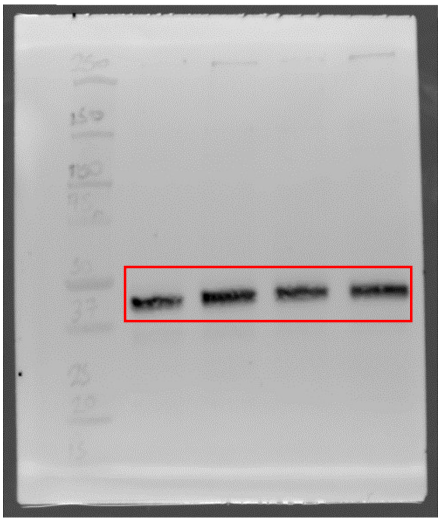

NANOG MW 34kDa

ESC1 ESC2

SCR CRISPRi SCR CRISPRi

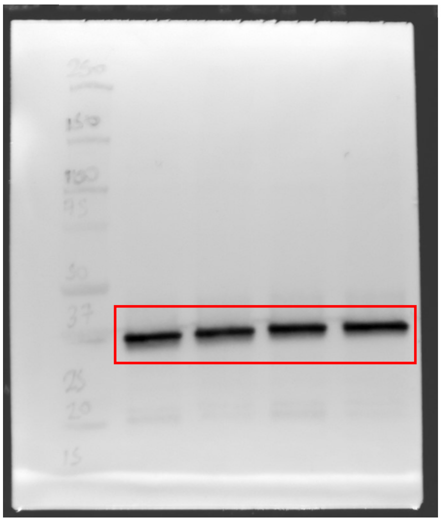

GAPDH MW 36kDa

2° REPLICATE

ESC1 ESC2

SCR CRISPRi SCR CRISPRi

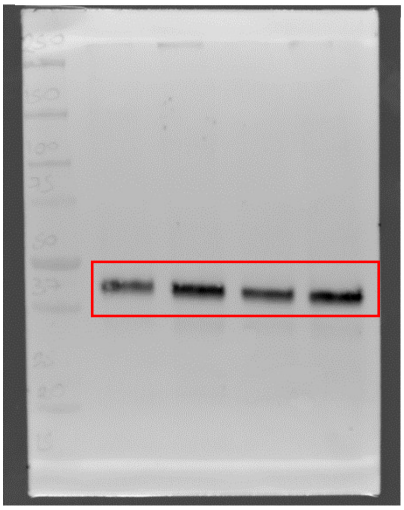

NANOG MW 34kDa

ESC1 ESC2

SCR CRISPRi SCR CRISPRi

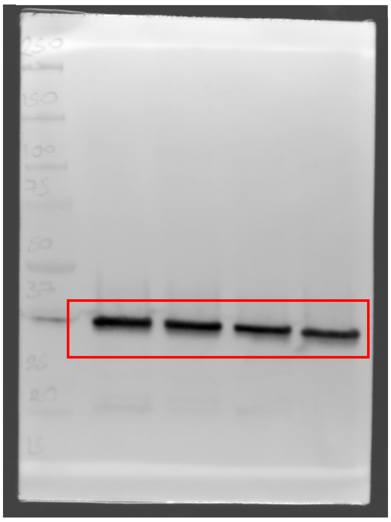

GAPDH MW 36kDa

3° REPLICATE

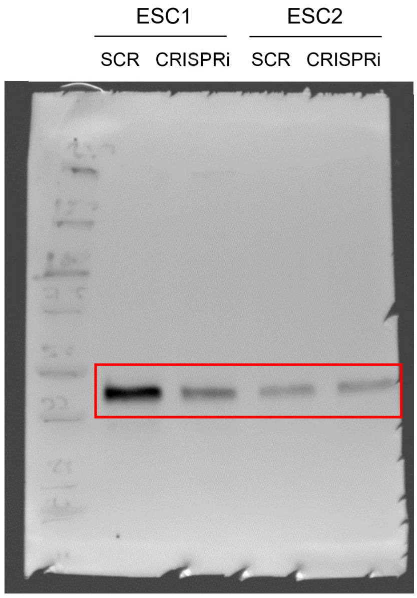

NANOG MW 34kDa

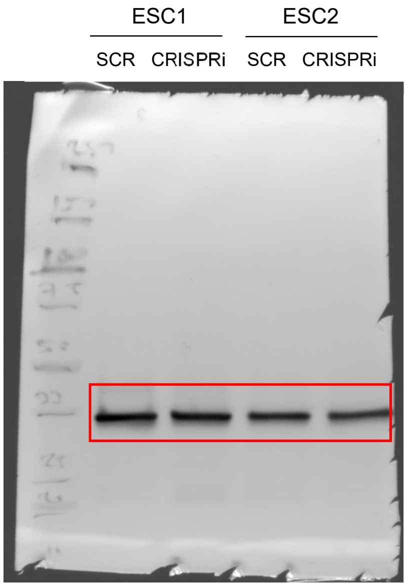

GAPDH MW 36kDa

FIG. 3E

1° REPLICATE

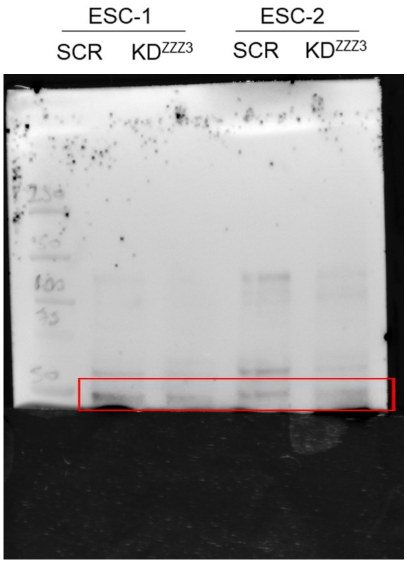

c-MYC MW 62kDa

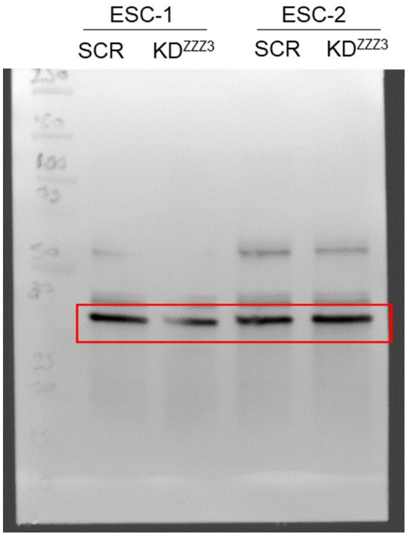

GAPDH MW 36kDa

2° REPLICATE

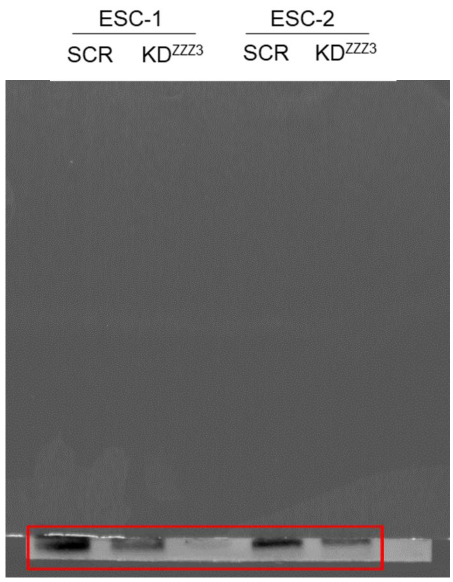

c-MYC MW 62kDa

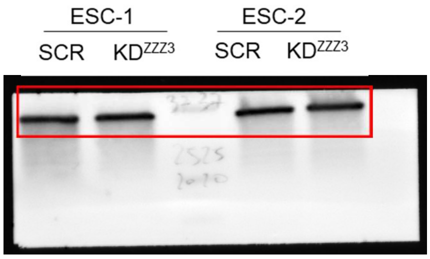

GAPDH MW 36kDa

3° REPLICATE

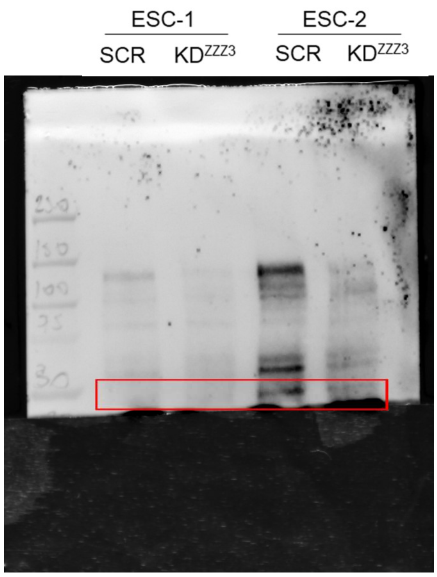

c-MYC MW 62kDa

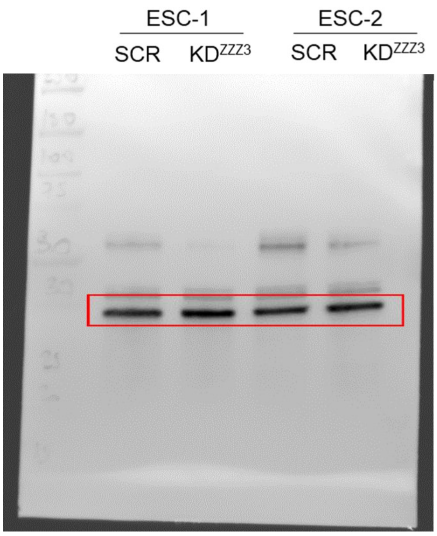

GAPDH MW 36kDa

1° REPLICATE

ESC-1 ESC-2

SCR KD^{ZZZ3} SCR KD^{ZZZ3}

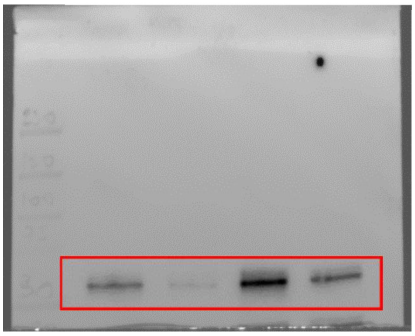

E2F4a MW 62kDa

ESC-1 ESC-2

SCR KD^{ZZZ3} SCR KD^{ZZZ3}

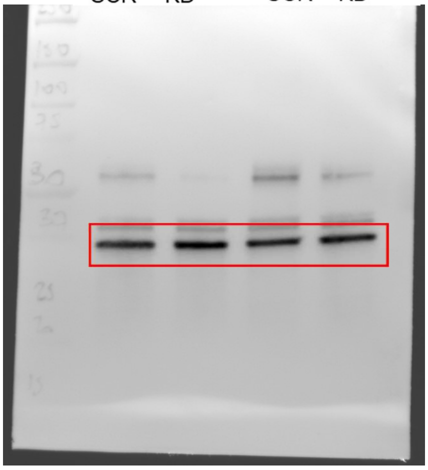

GAPDH MW 36kDa

2° REPLICATE

ESC-1 ESC-2

SCR KD^{ZZZ3} SCR KD^{ZZZ3}

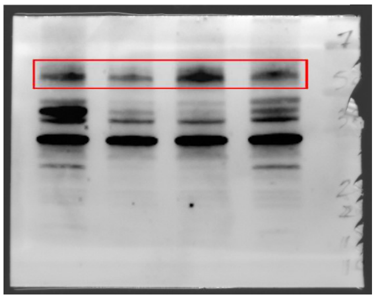

E2F4a MW 62kDa

ESC-1 ESC-2

SCR KD^{ZZZ3} SCR KD^{ZZZ3}

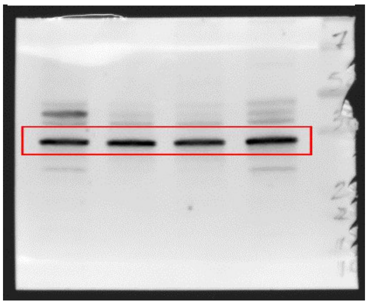

GAPDH MW 36kDa

3° REPLICATE

ESC-1 ESC-2

SCR KD^{ZZZ3} SCR KD^{ZZZ3}

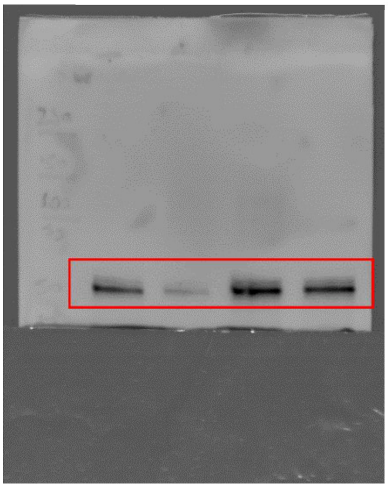

E2F4a MW 62kDa

ESC-1 ESC-2

SCR KD^{ZZZ3} SCR KD^{ZZZ3}

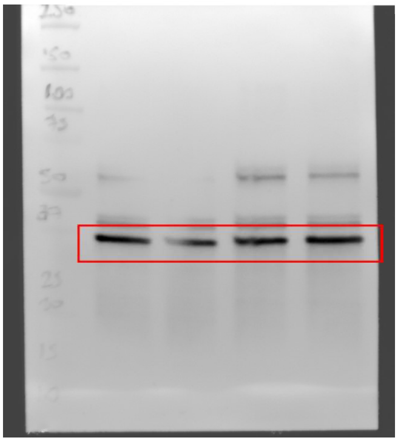

GAPDH MW 36kDa

FIG. 5C

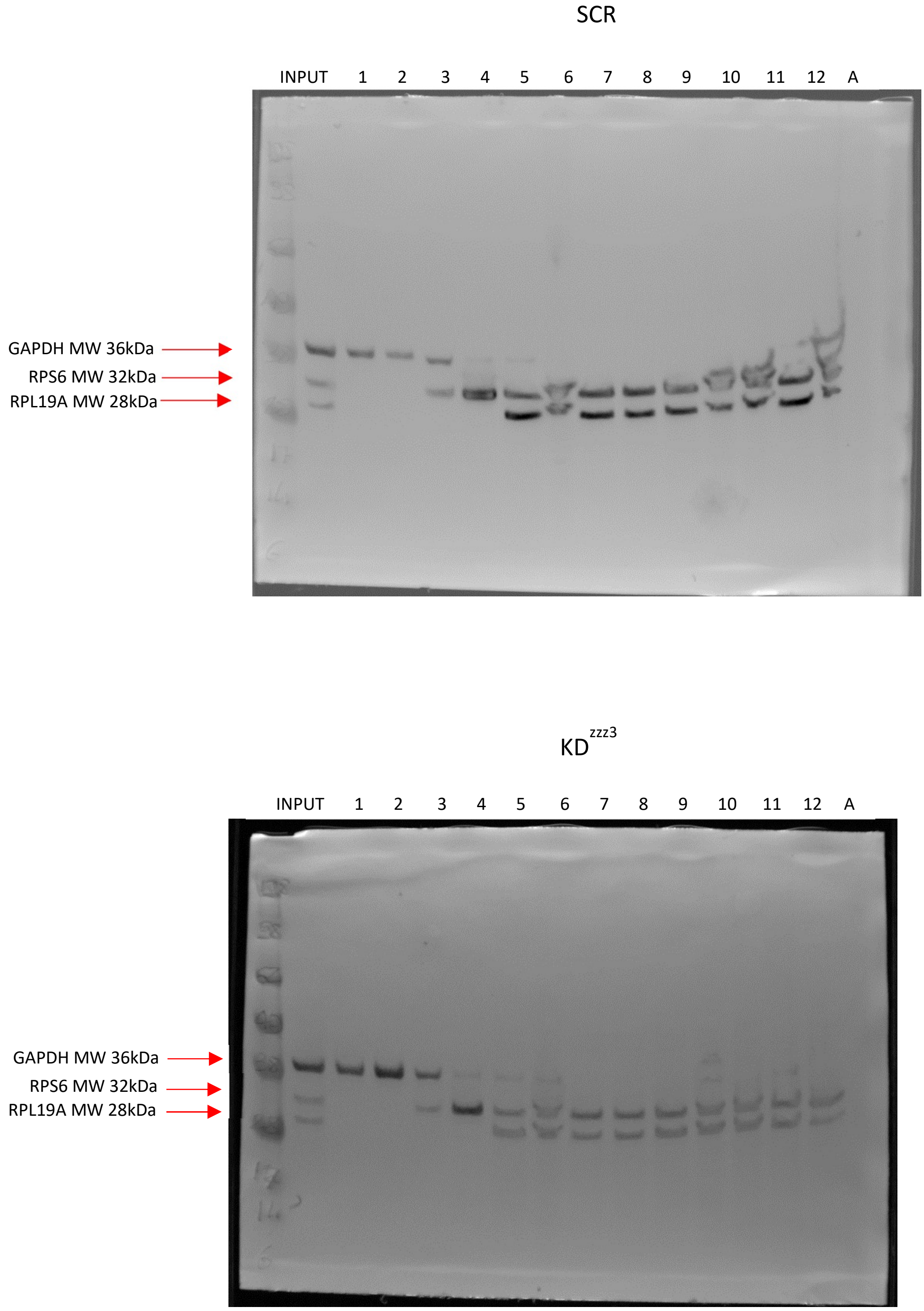
SCR
INPUT 1 2 3 4 5 6 7 8 9 10 11 12 A
GAPDH MW 36kDa
RPS6 MW 32kDa
RPL19A MW 28kDa
KD zzz3
INPUT 1 2 3 4 5 6 7 8 9 10 11 12 A
GAPDH MW 36kDa
RPS6 MW 32kDa
RPL19A MW 28kDa

FIG. 5E

1° REPLICATE

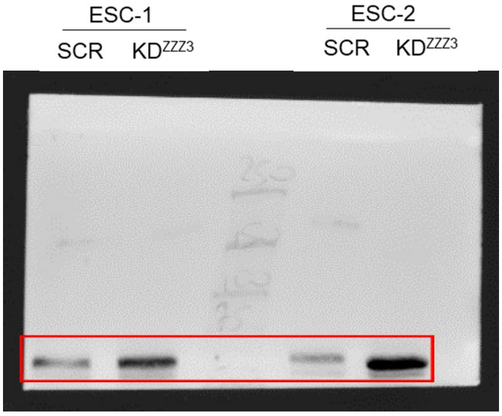

P53 MW 53kDa

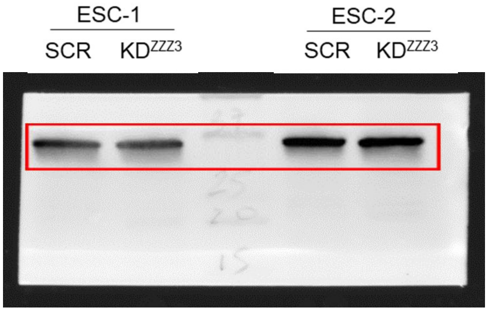

GAPDH MW 36kDa

2° REPLICATE

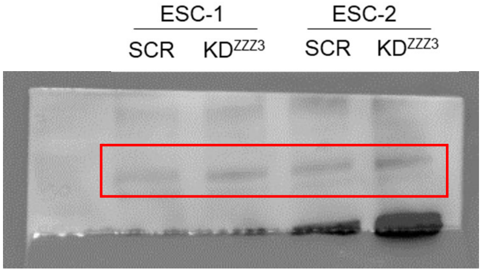

P53 MW 53kDa

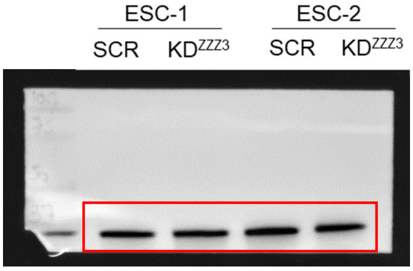

GAPDH MW 36kDa

3° REPLICATE

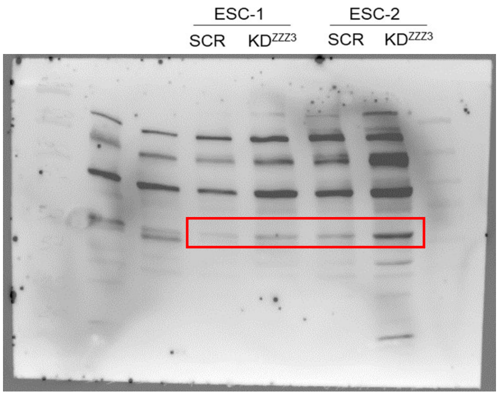

P53 MW 53kDa

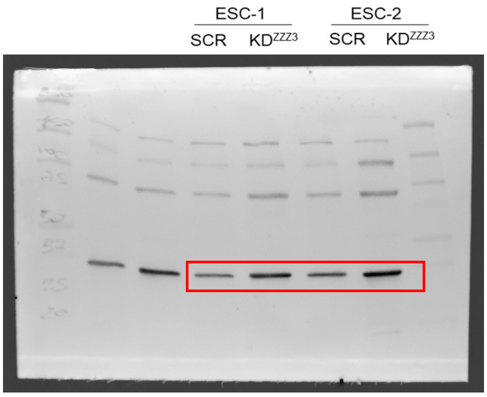

GAPDH MW 36kDa

FIG. 6B

1° REPLICATE

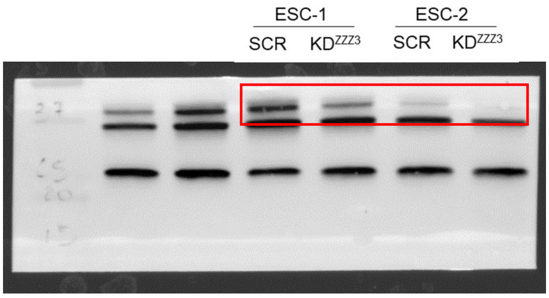

p-ERK1/2 (Thr202/Tyr204) MW 42kDa

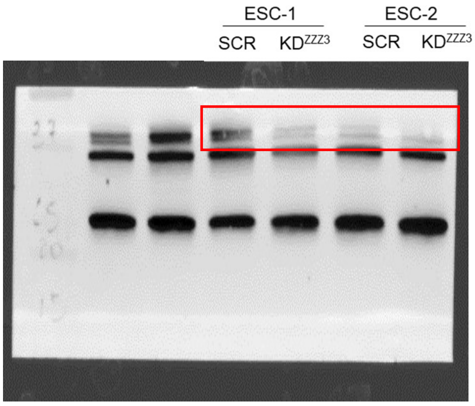

ERK1/2 MW 42kDa

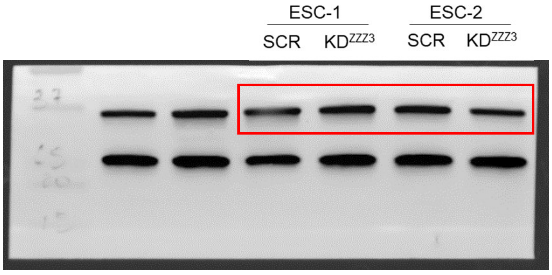

GAPDH MW 36kDa

2° REPLICATE

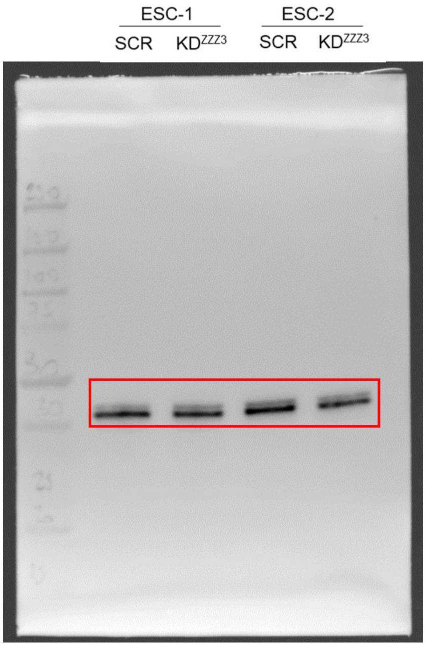

p-ERK1/2 (Thr202/Tyr204) MW 42kDa

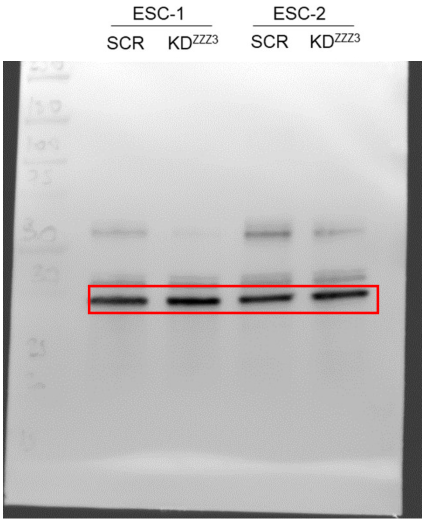

GAPDH MW 36kDa

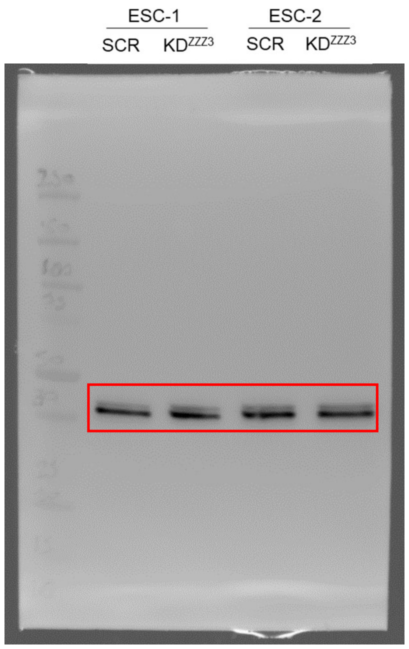

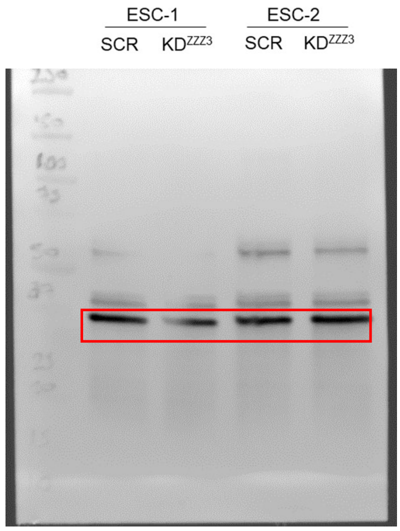

ERK1/2 MW 42kDa

GAPDH MW 36kDa

3° REPLICATE

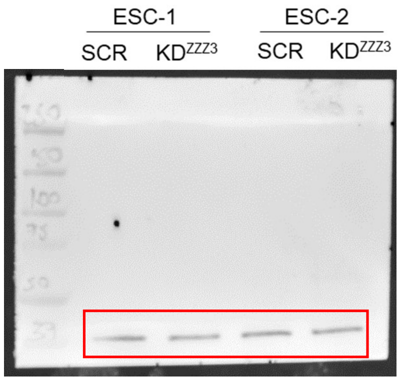

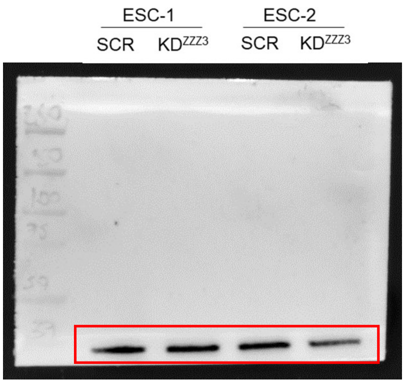

p-ERK1/2 (Thr202/Tyr204) MW 42kDa

GAPDH MW 36kDa

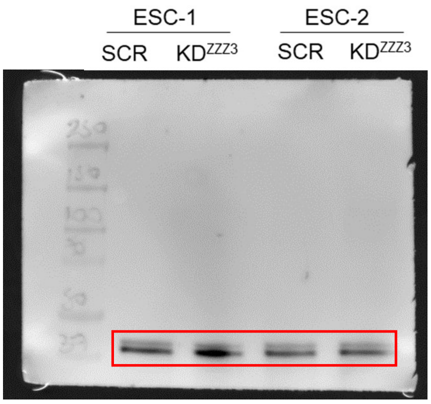

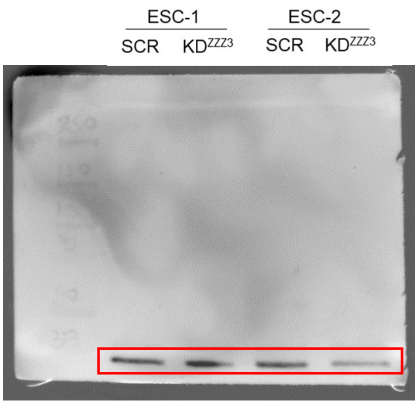

ERK1/2 MW 42kDa

GAPDH MW 36kDa

FIG. 6C

1° REPLICATE

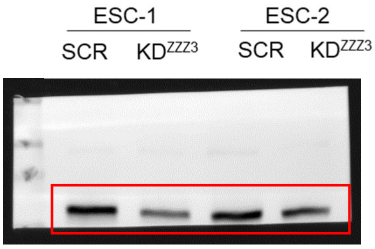

p-AKT (S473) MW 60kDa

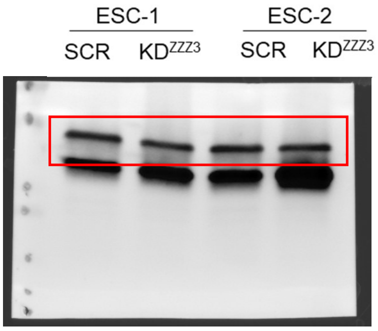

GAPDH MW 36kDa

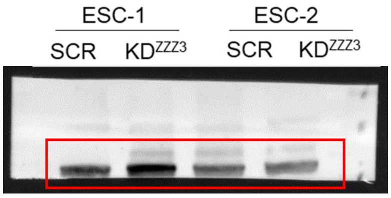

AKT MW 60kDa

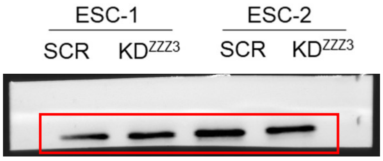

GAPDH MW 36kDa

2° REPLICATE

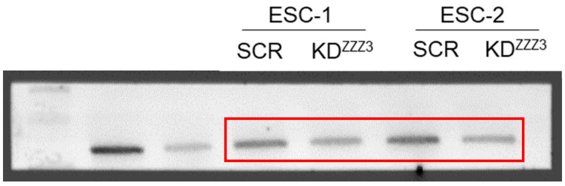

p-AKT (S473) MW 60kDa

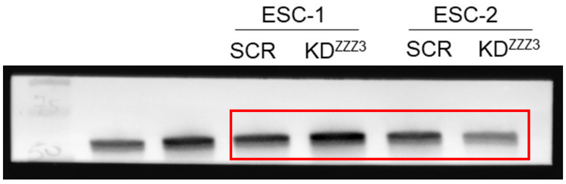

AKT MW 60kDa

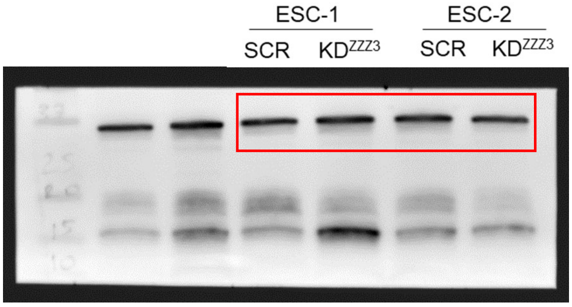

GAPDH MW 36kDa

3° REPLICATE

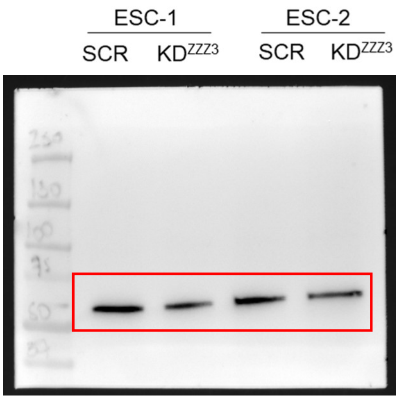

p-AKT (S473) MW 60kDa

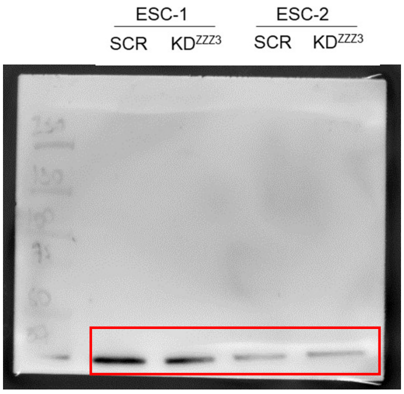

GAPDH MW 36kDa

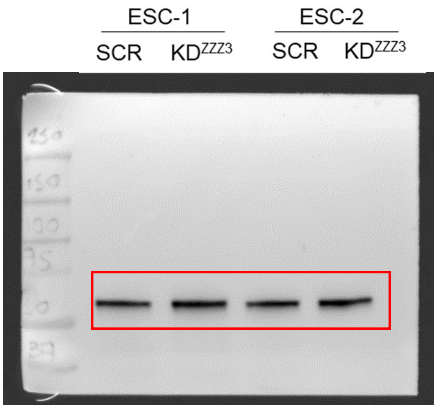

AKT MW 60kDa

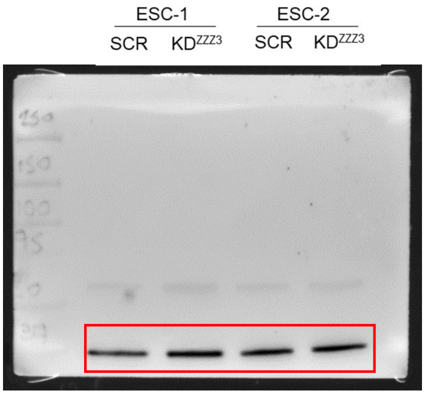

GAPDH MW 36kDa

FIG. 6D

1° REPLICATE

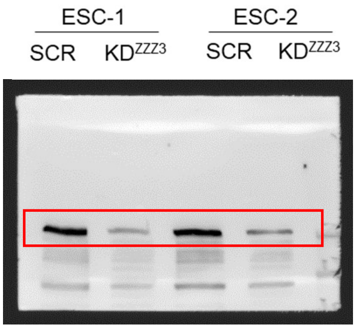

p-mTOR(S2448) MW 289kDa

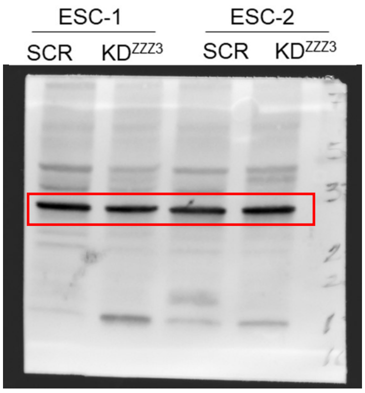

GAPDH MW 36kDa

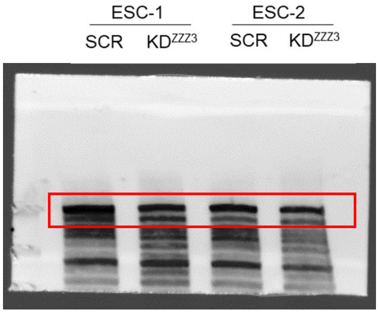

mTOR MW 289kDa

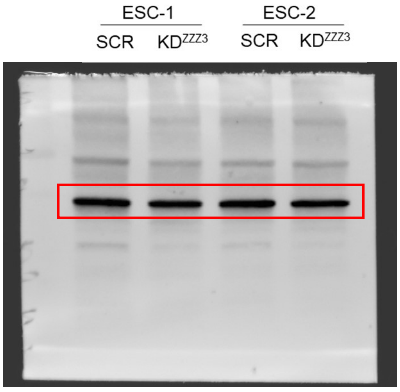

GAPDH MW 36kDa

2° REPLICATE

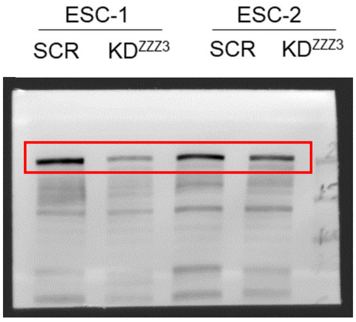

p-mTOR(S2448) MW 289kDa

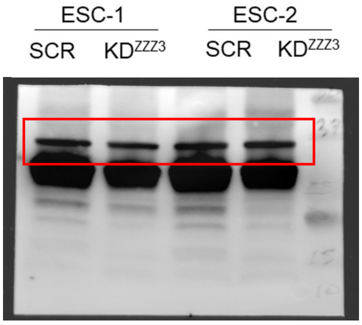

GAPDH MW 36kDa

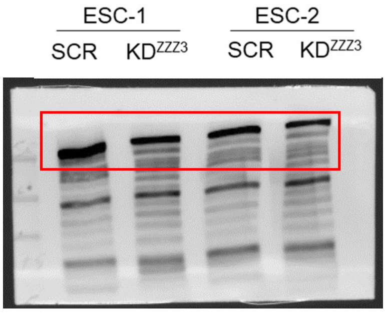

mTOR MW 289kDa

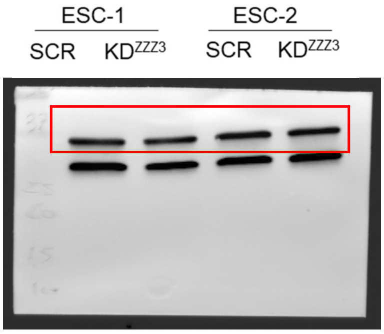

GAPDH MW 36kDa

3° REPLICATE

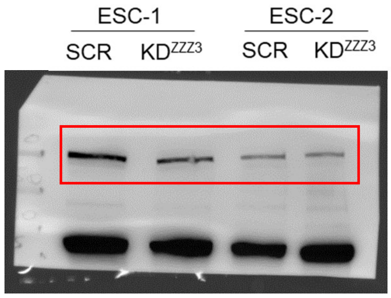

p-mTOR(S2448) MW 289kDa

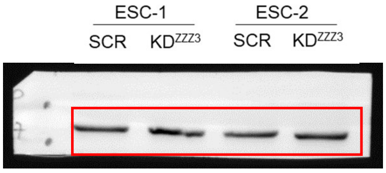

ACTIN MW 42kDa

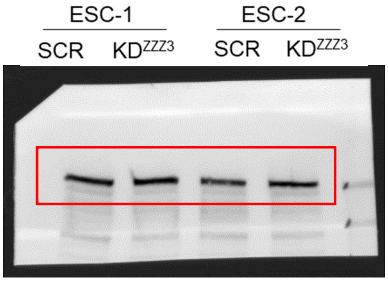

mTOR MW 289kDa

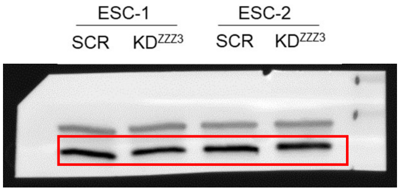

GAPDH MW 36kDa

FIG. 6E

1° REPLICATE

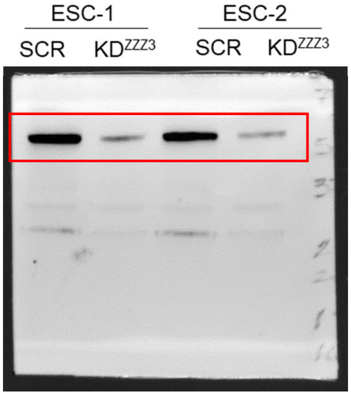

p-P70S6K (T421/S424) MW 70kDa

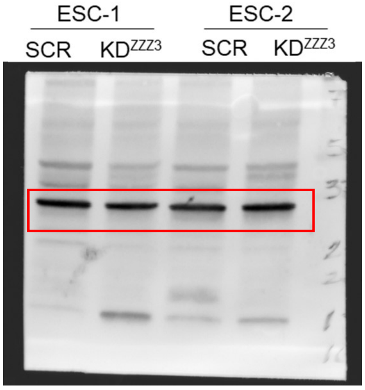

GAPDH MW 36kDa

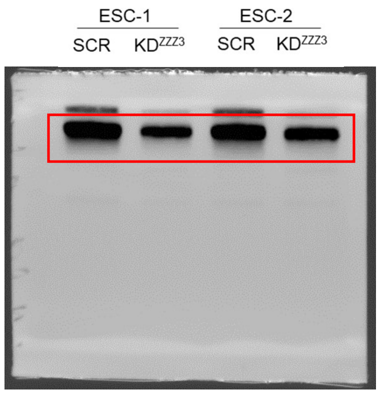

P70S6K MW 70kDa

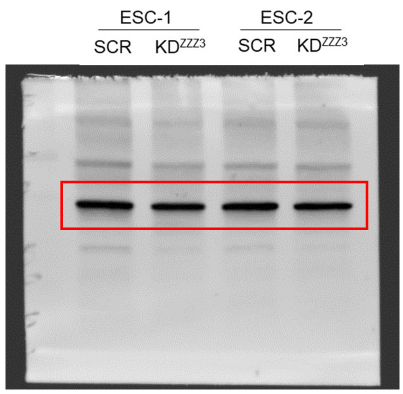

GAPDH MW 36kDa

2° REPLICATE

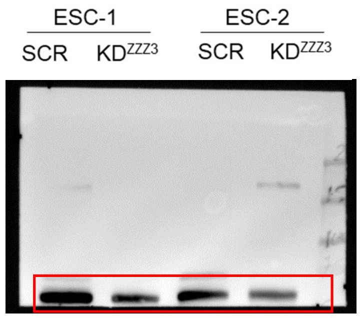

p-P70S6K (T421/S424) MW 70kDa

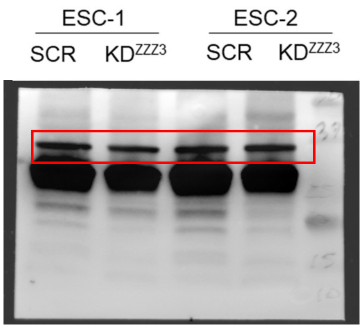

GAPDH MW 36kDa

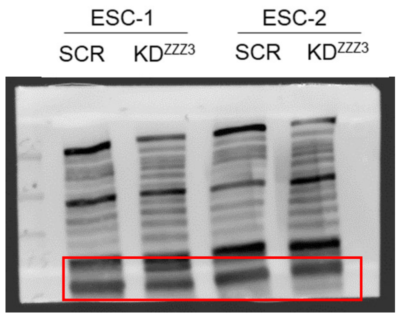

P70S6K MW 70kDa

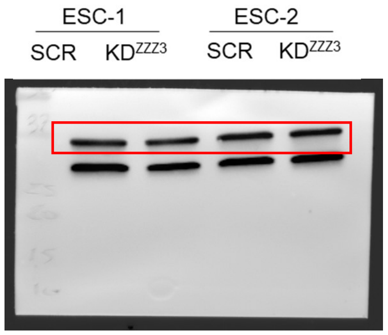

GAPDH MW 36kDa

3° REPLICATE

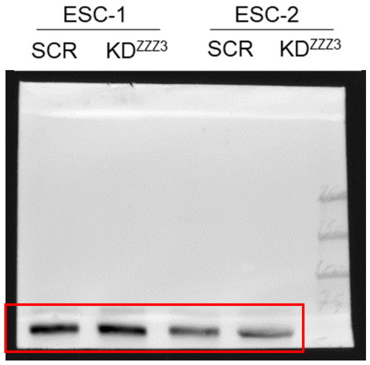

p-P70S6K (T421/S424) MW 70kDa

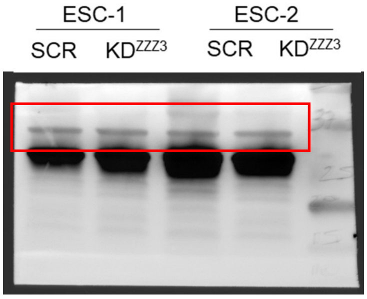

GAPDH MW 36kDa

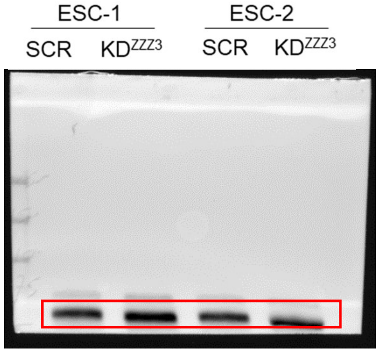

P70S6K MW 70kDa

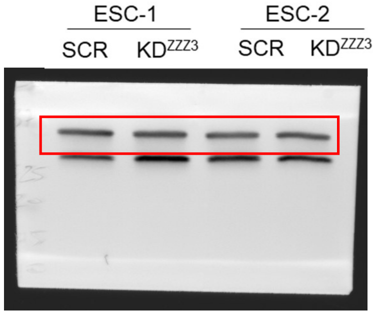

GAPDH MW 36kDa

FIG. 6F

1° REPLICATE

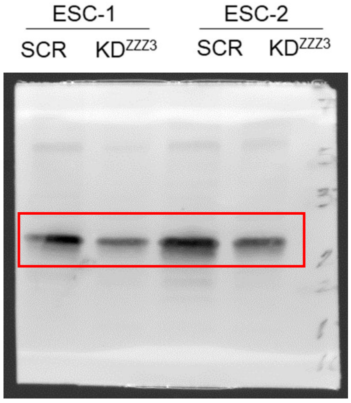

p-RPS6 (S235) MW 32kDa

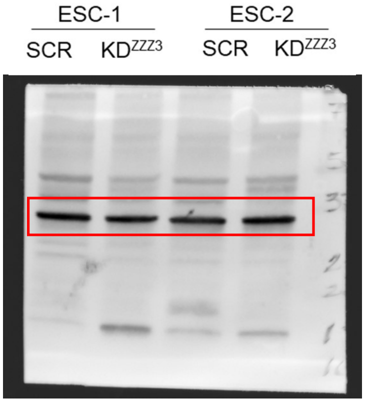

GAPDH MW 36kDa

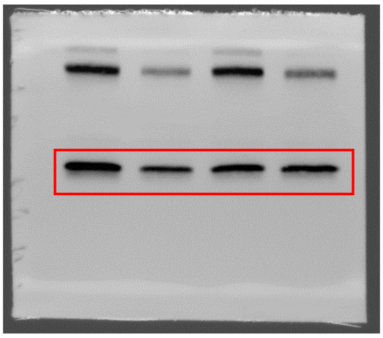

RPS6 MW 32kDa

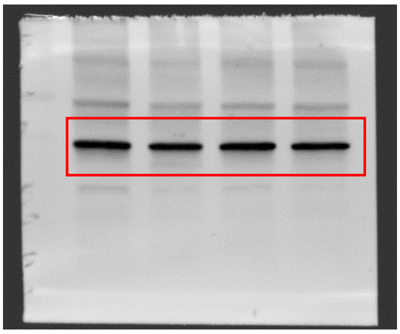

GAPDH MW 36kDa

2° REPLICATE

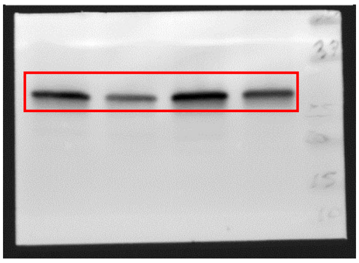

p-RPS6 (S235) MW 32kDa

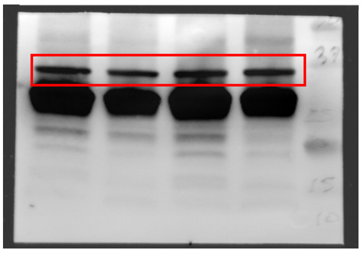

GAPDH MW 36 kDa

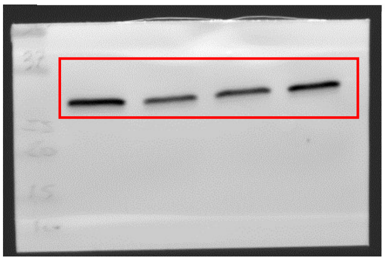

RPS6 MW 32kDa

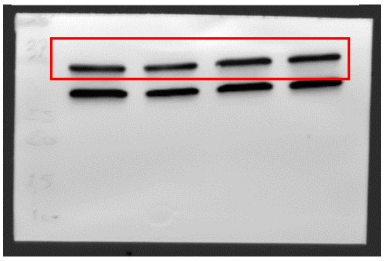

GAPDH MW 36kDa

3° REPLICATE

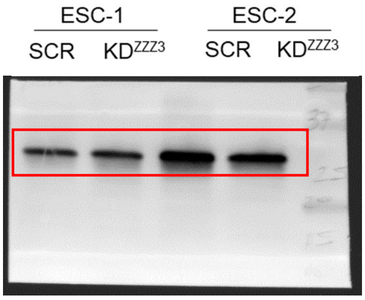

p-RPS6 (S235) MW 32kDa

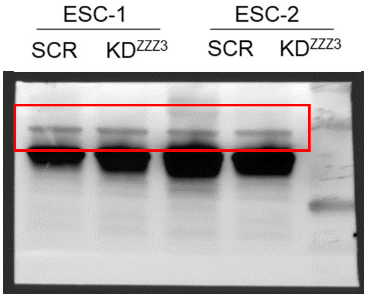

GAPDH MW 36kDa

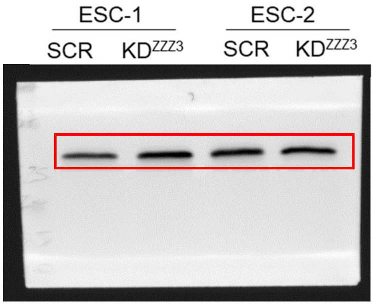

RPS6 MW 32kDa

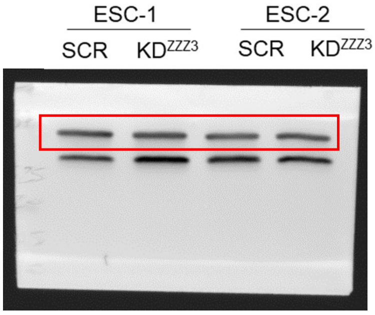

GAPDH MW 36kDa

FIG. 6G

1° REPLICATE

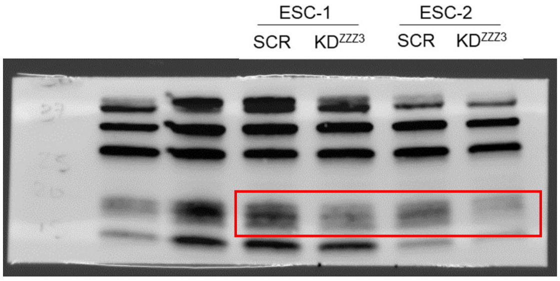

p-4E-BP1(T37/46) MW 15-20kDa

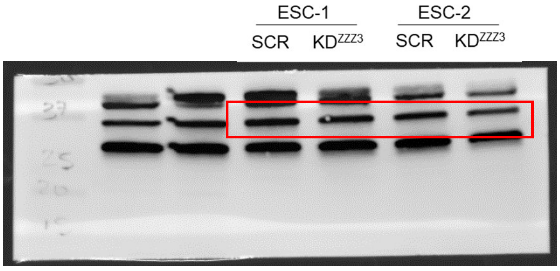

GAPDH MW 36kDa

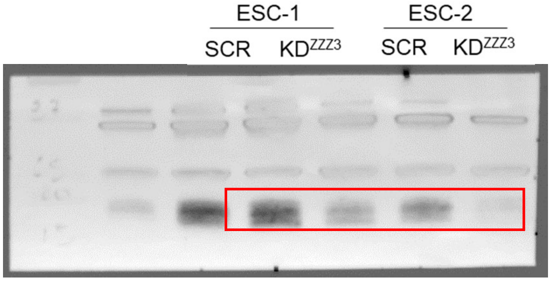

4E-BP1 MW 15-20kDa

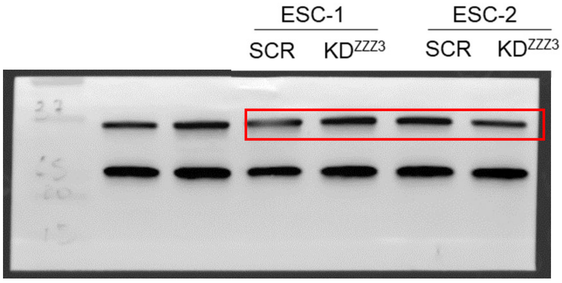

GAPDH MW 36kDa

2° REPLICATE

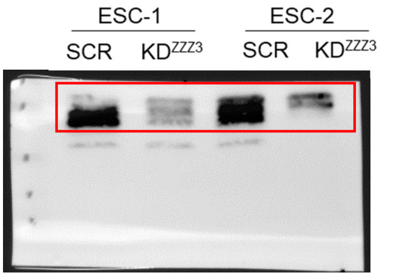

p-4E-BP1(T37/46) MW 15-20kDa

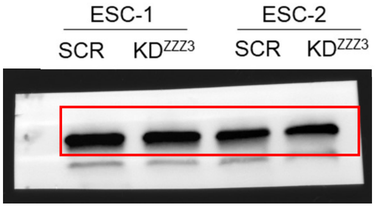

GAPDH MW 36kDa

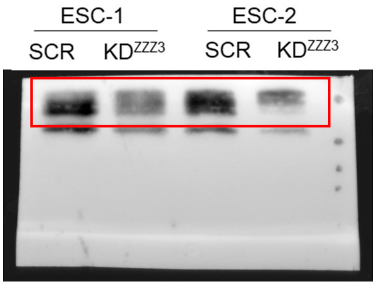

4E-BP1 MW 15-20kDa

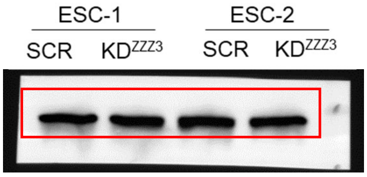

GAPDH MW 36kDa

3° REPLICATE

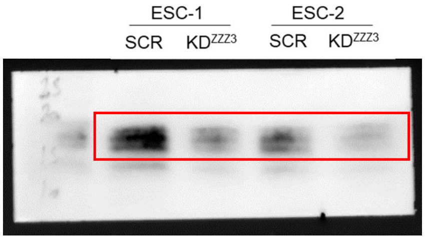

p-4E-BP1(T37/46) MW 15-20kDa

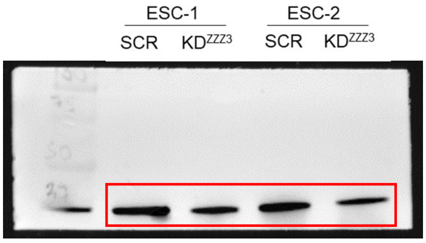

GAPDH MW 36kDa

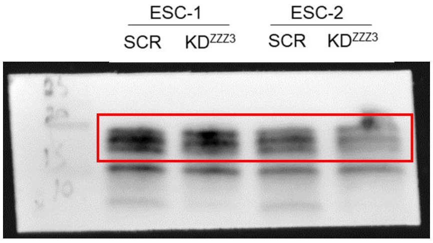

4E-BP1 MW 15-20kDa

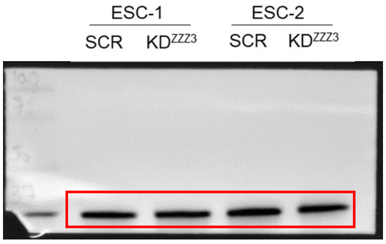

GAPDH MW 36kDa

FIG. 6H

1° REPLICATE

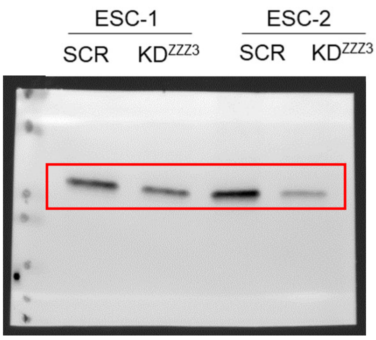

p-EIF4E (S209) MW 25kDa

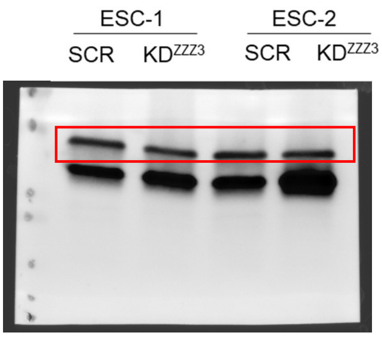

GAPDH MW 36kDa

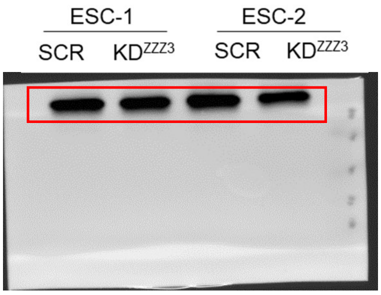

EIF4E MW 25kDa

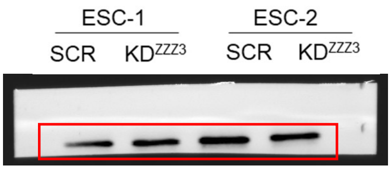

GAPDH MW 36kDa

2° REPLICATE

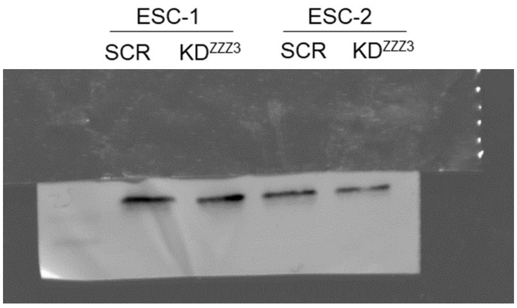

p-EIF4E (S209) MW 25kDa

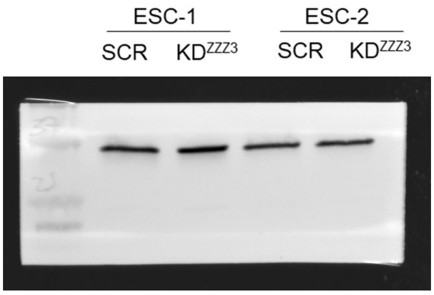

GAPDH MW 36kDa

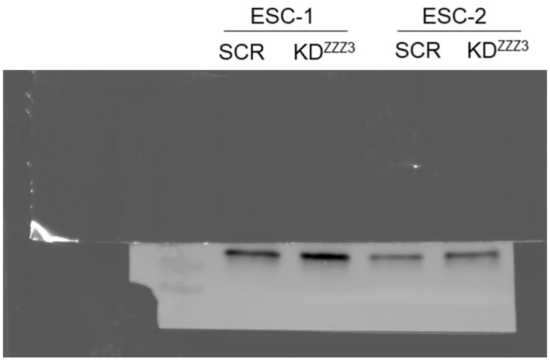

EIF4E MW 25kDa

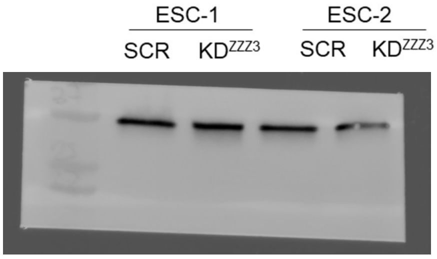

GAPDH MW 36kDa

3° REPLICATE

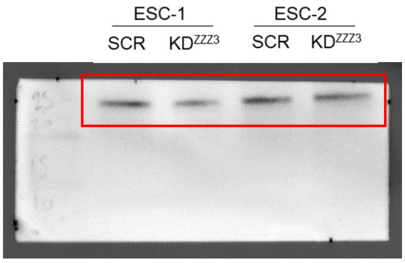

p-EIF4E (S209) MW 25kDa

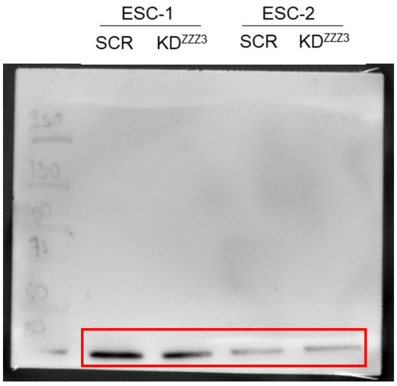

GAPDH MW 36kDa

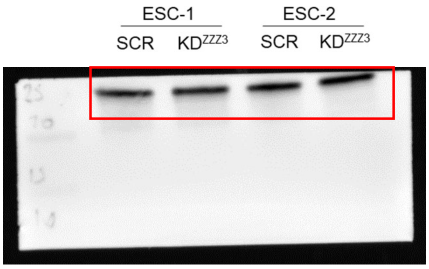

EIF4E MW 25kDa

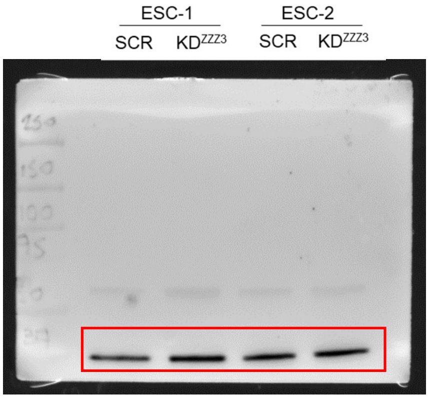

GAPDH MW 36kDa

SUPPLEMENTARY S1D

ESC-1 Input IP IgG

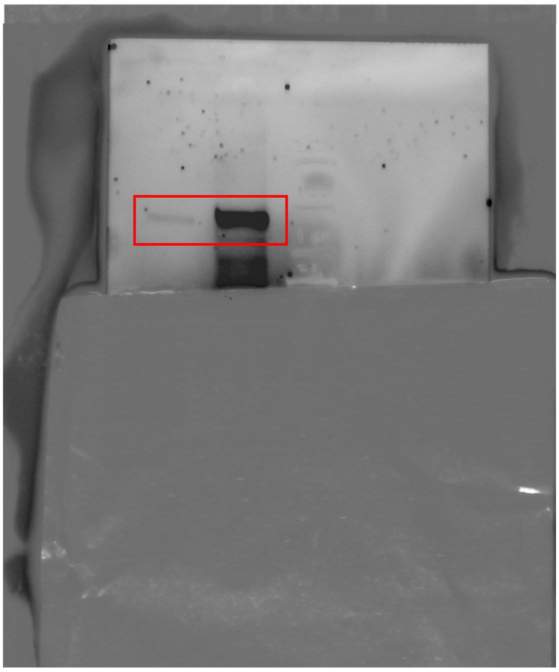

ESC-2 Input IP IgG

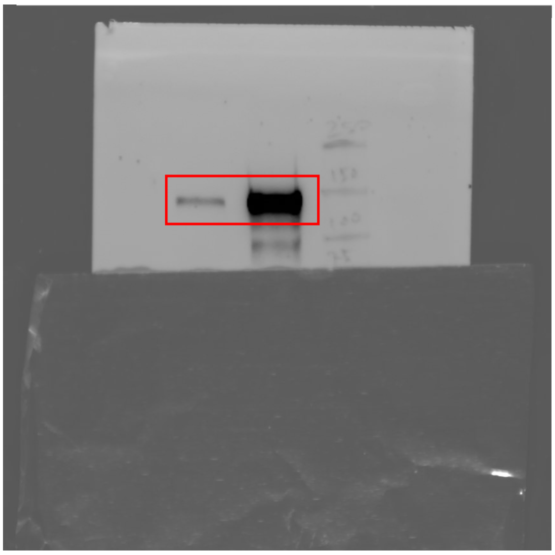

ZZZ3 MW102kDa

ESC-1 Input IP IgG

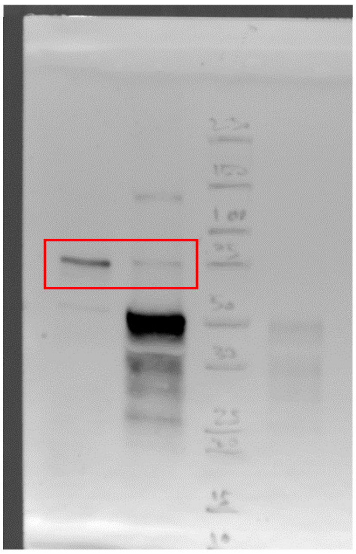

ESC-2 Input IP IgG

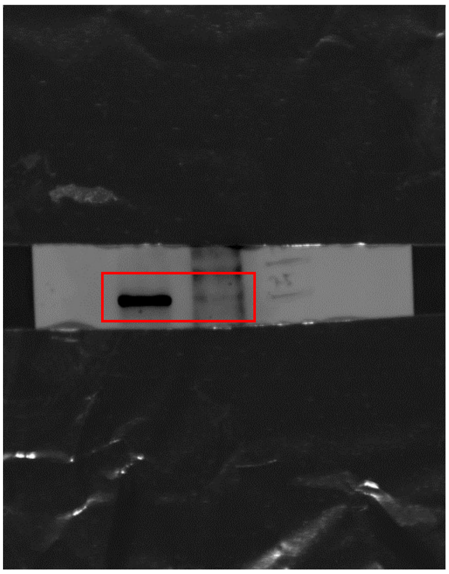

DDX18 MW75kDa

ESC-1

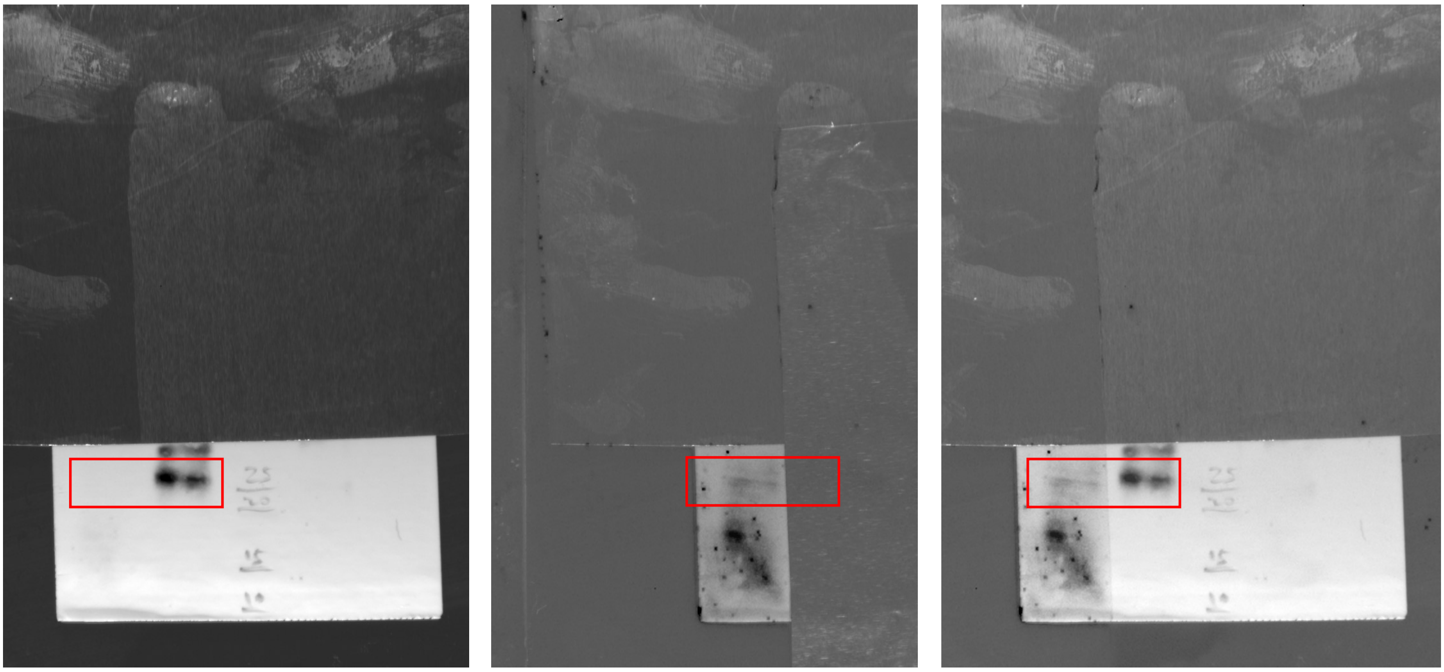

Input IP IgG | Input | Input IP IgG

RPS6 MW32kDa

Since the exposition time required for the Input and IP bands were significantly different, aluminium foil was used to avoid the oversaturation of the IP band. Here we reported the first exposition, the second exposition for the Input band acquisition and the final merge.

ESC-2 Input IP IgG

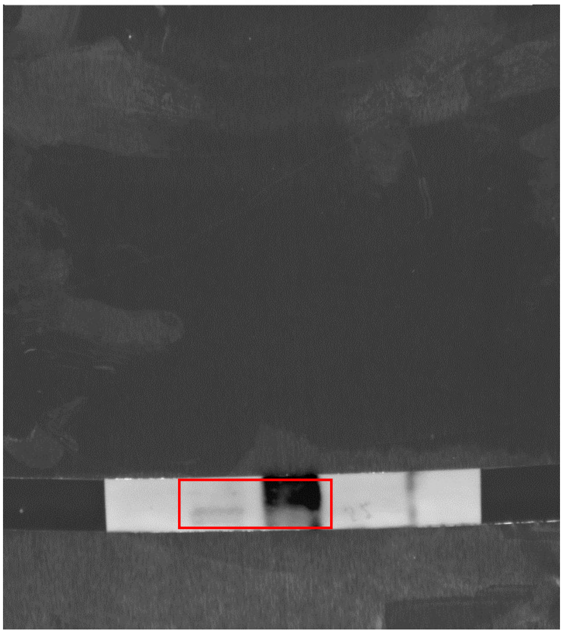

RPS6 MW32kDa

ESC-1 Input IP IgG

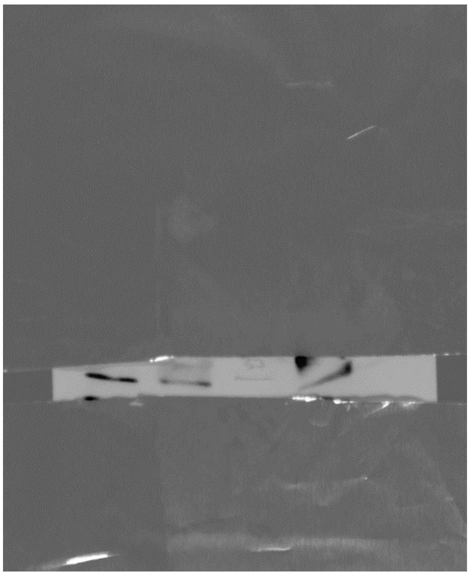

Fibrillarin MW35kDa

ECS-2

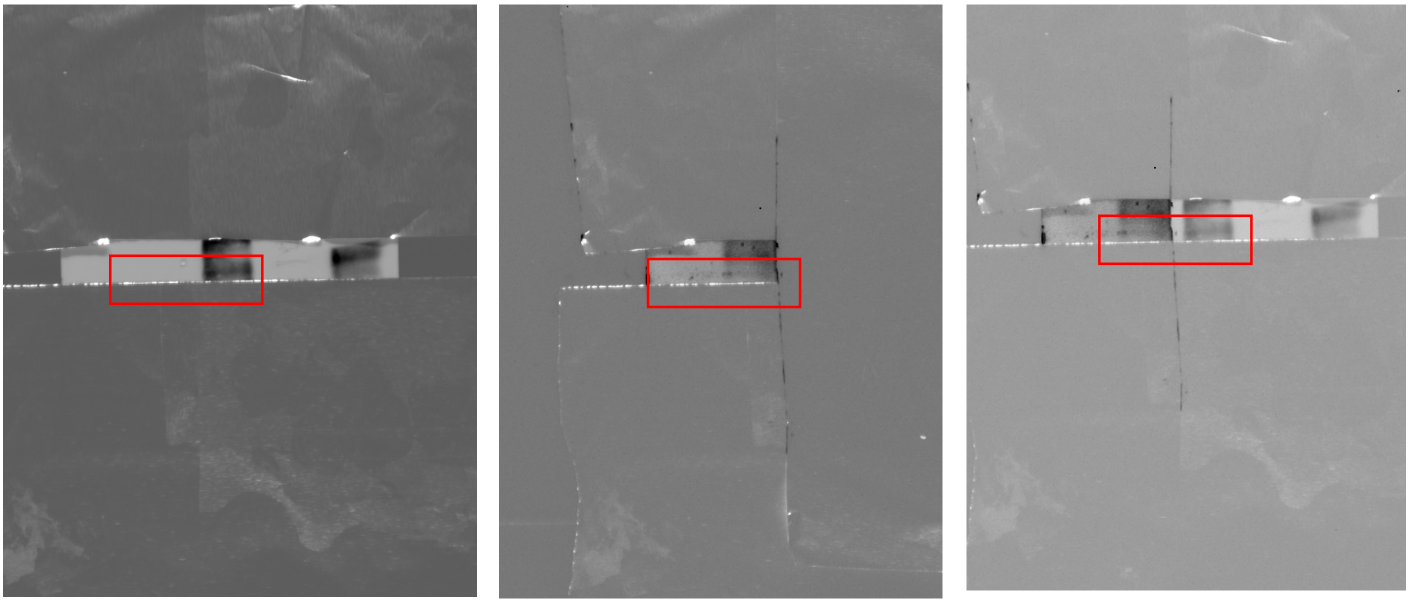

Input IP IgG

Input

Input IP IgG

Fibrillarin MW35kDa

Since the exposition time required for the Input and IP bands were significantly different, aluminium foil was used to avoid the oversaturation of the IP band. Here we reported the first exposition, the second exposition for the Input band acquisition and the final merge.

SUPPLEMENTARY S2B

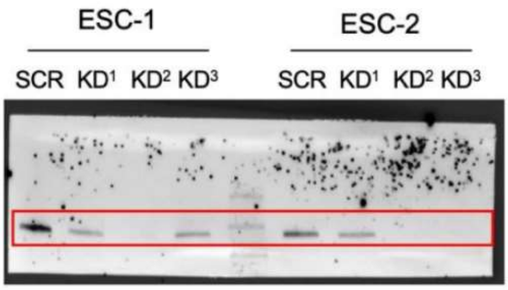

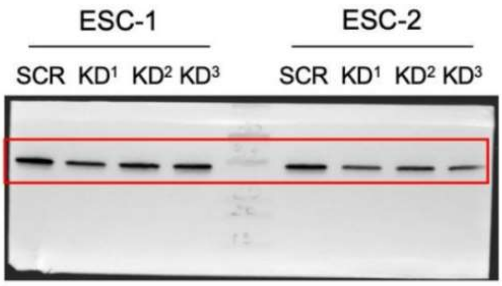

ZZZ3 MW102kDa

GAPDH MW36kDa

SUPPLEMENTARY S2D

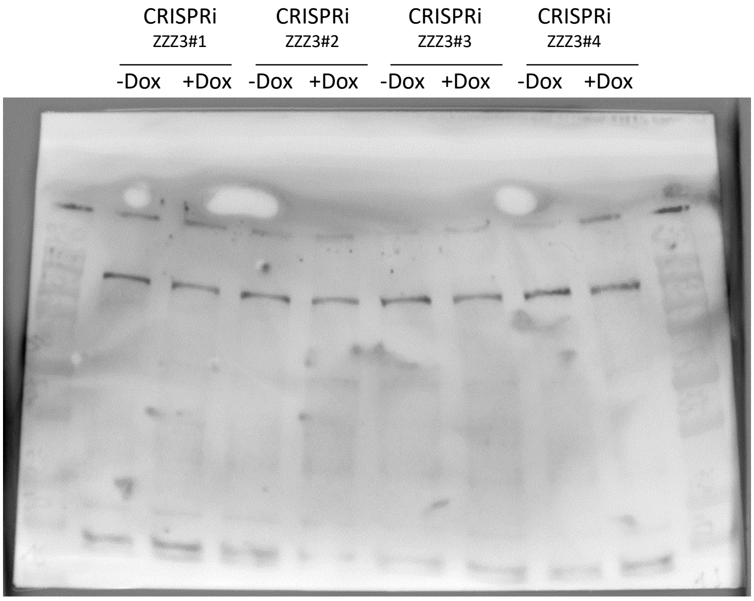

ZZZ3 MW 102kDa

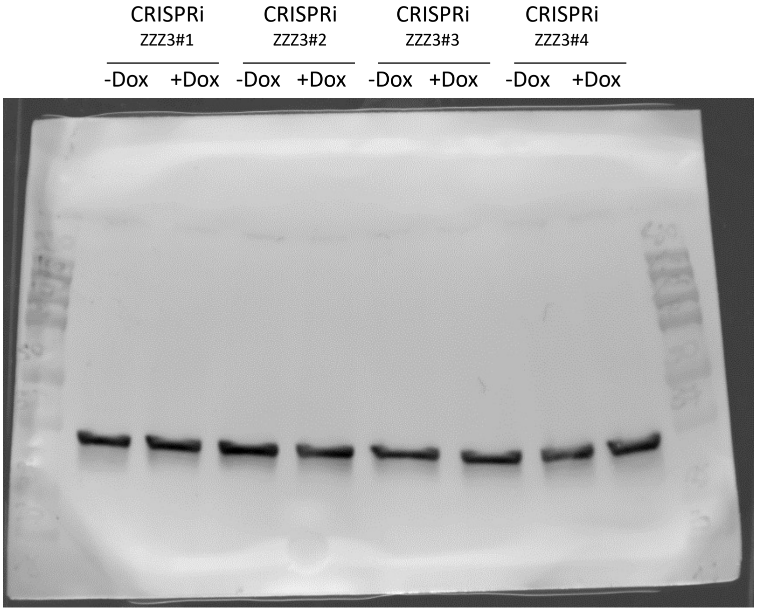

GAPDH MW 36kDa

SUPPLEMENTARY S2F

TET SCR | TET-sh$^{ZZZ3\#1}$ -Dox +Dox | TET-sh$^{ZZZ3\#2}$ -Dox +Dox

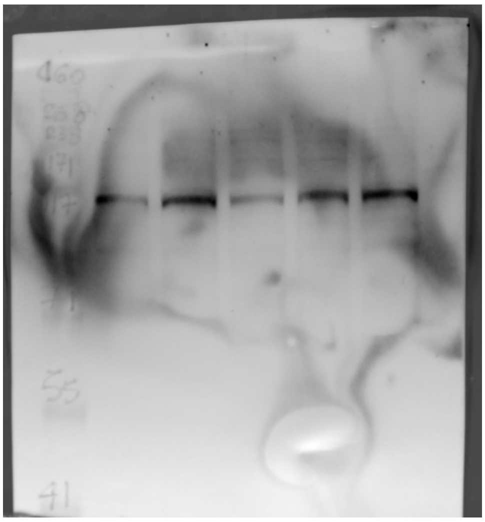

ZZZ3 MW 102kDa

TET SCR | TET-sh$^{ZZZ3\#1}$ -Dox +Dox | TET-sh$^{ZZZ3\#2}$ -Dox +Dox

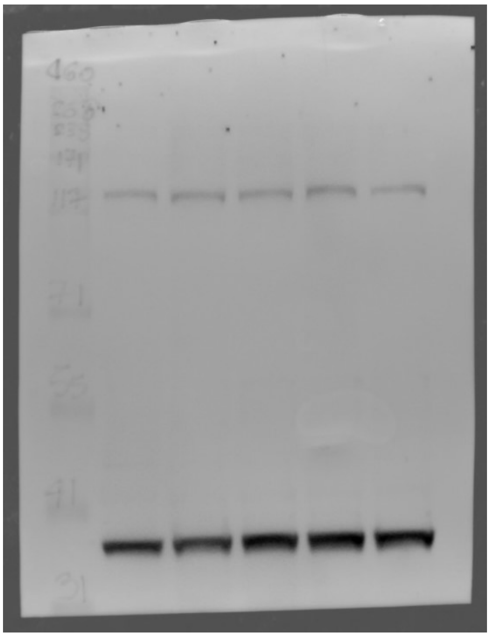

GAPDH MW 36kDa

SUPPLEMENTARY S4F

1° REPLICATE

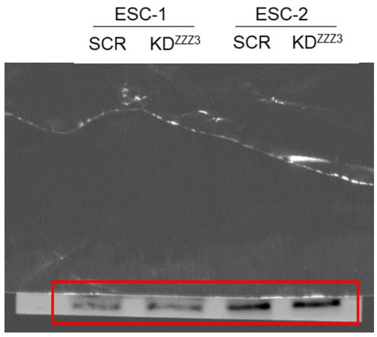

Cleaved Caspase-9 MW 39kDa

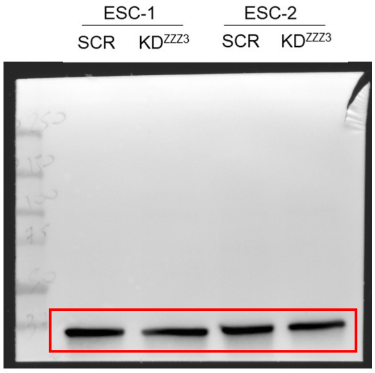

GAPDH MW 36kDa

2° REPLICATE

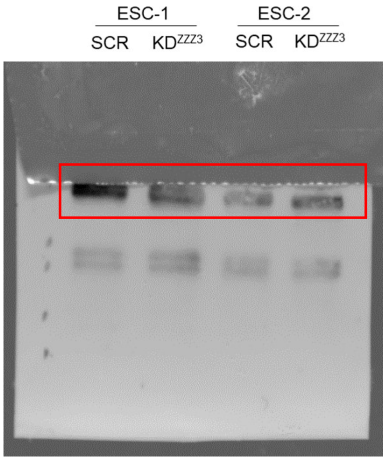

Cleaved Caspase-9 MW 39kDa

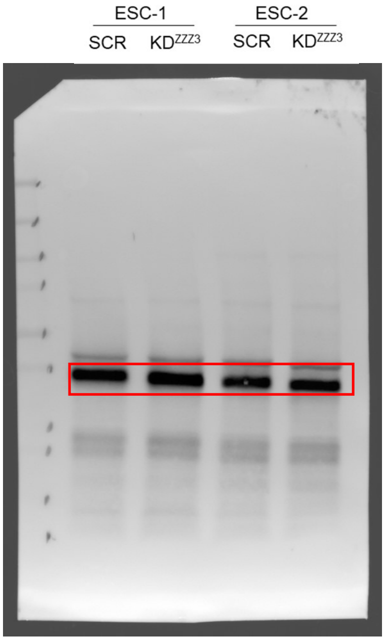

GAPDH MW 36kDa

3° REPLICATE

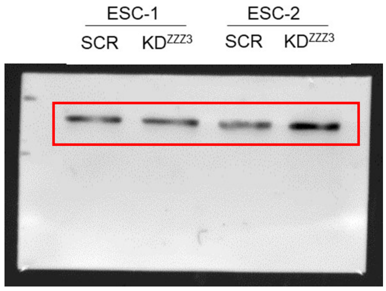

Cleaved Caspase-9 MW 39kDa

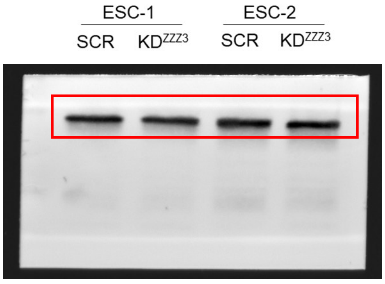

GAPDH MW 36kDa

1° REPLICATE

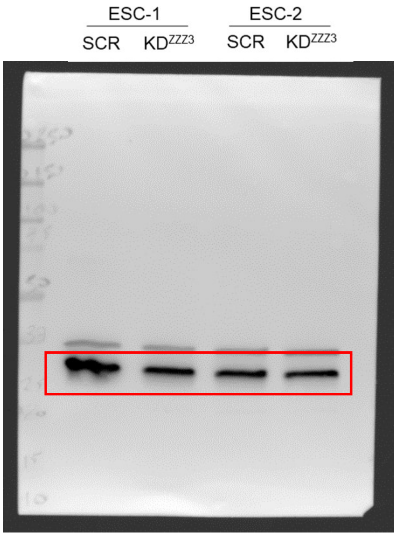

Caspase- 3 MW 35kDa

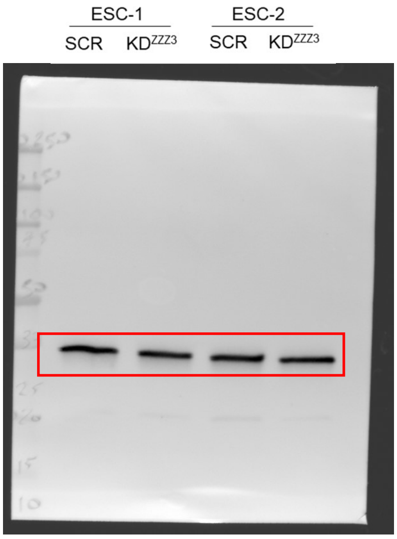

GAPDH MW 36kDa

2° REPLICATE

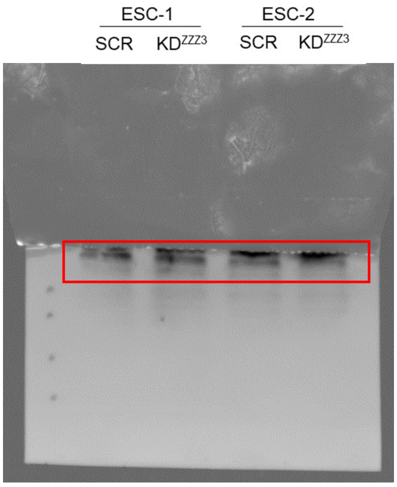

Caspase- 3 MW 35kDa

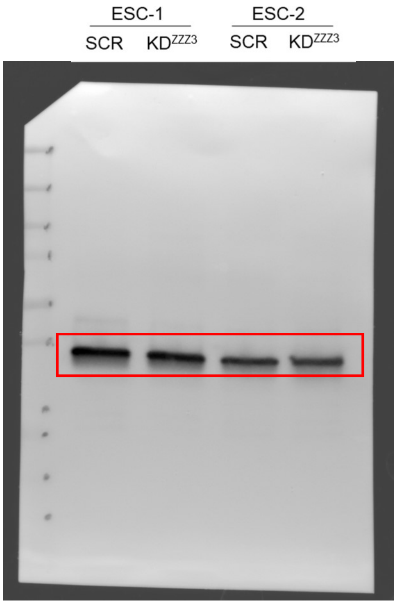

GAPDH MW 36kDa

3° REPLICATE

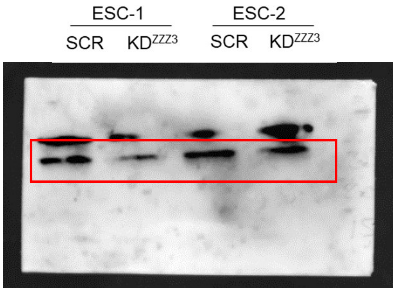

Caspase- 3 MW 35kDa

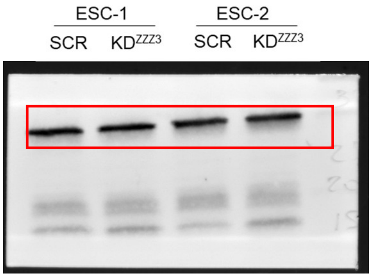

GAPDH MW 36kDa

SUPPLEMENTARY S5A

1° REPLICATE

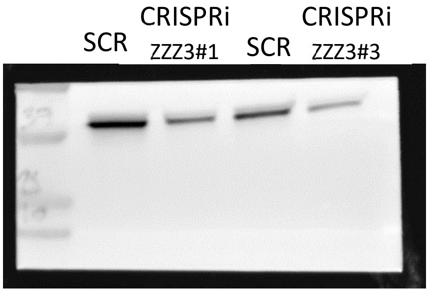

p-ERK1/2 (Thr202/Tyr204) MW 42kDa

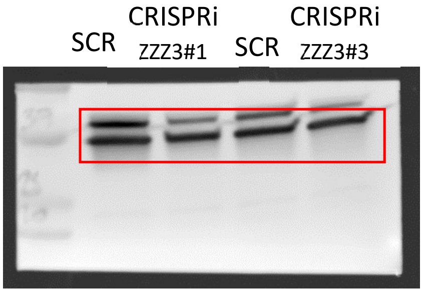

GAPDH MW 36kDa

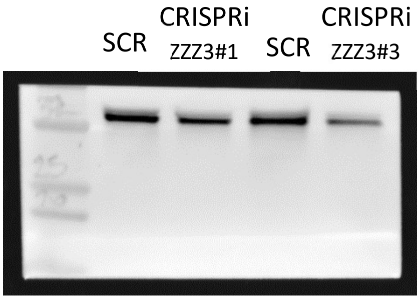

ERK1/2 MW 42kDa

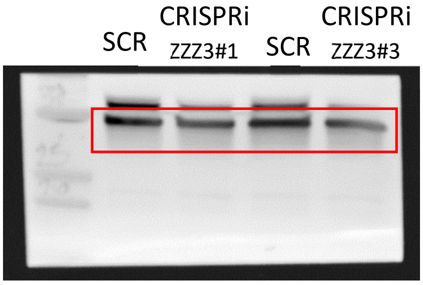

GAPDH MW 36kDa

2° REPLICATE

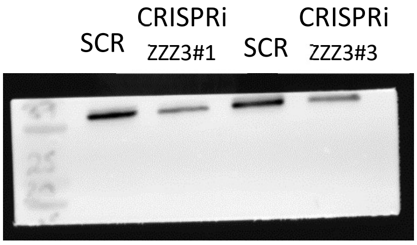

p-ERK1/2(Thr202/Tyr204) MW42kDa

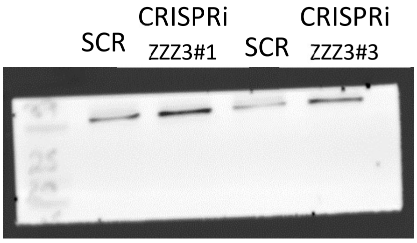

ERK1/2 MW 42kDa

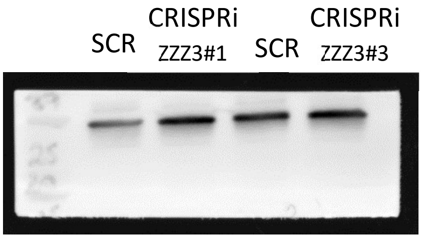

GAPDH MW 36kDa

3° REPLICATE

SCR | CRISPRi ZZZ3#1 | SCR | CRISPRi ZZZ3#3

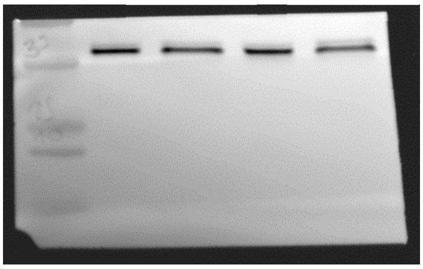

p-ERK1/2 (Thr202/Tyr204) MW 42kDa

SCR | CRISPRi ZZZ3#1 | SCR | CRISPRi ZZZ3#3

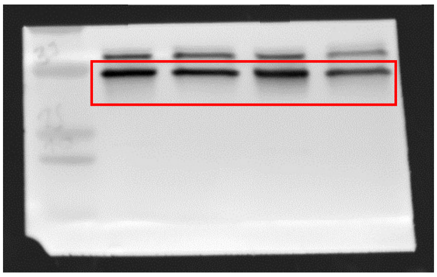

GAPDH MW 36kDa

SCR | CRISPRi ZZZ3#1 | SCR | CRISPRi ZZZ3#3

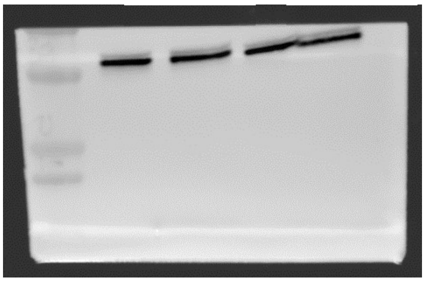

ERK1/2 MW 42kDa

SCR | CRISPRi ZZZ3#1 | SCR | CRISPRi ZZZ3#3

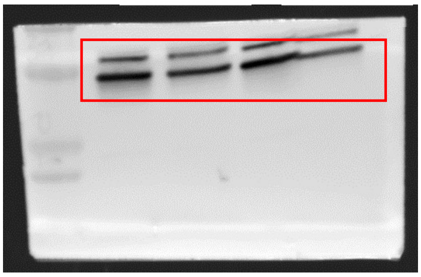

GAPDH MW 36kDa

1° REPLICATE

SCR | CRISPRi ZZZ3#1 | SCR | CRISPRi ZZZ3#3

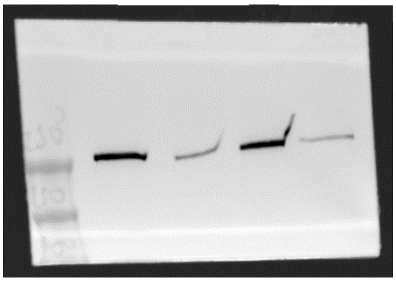

p-mTOR(S2448) MW 289kDa

SCR | CRISPRi ZZZ3#1 | SCR | CRISPRi ZZZ3#3

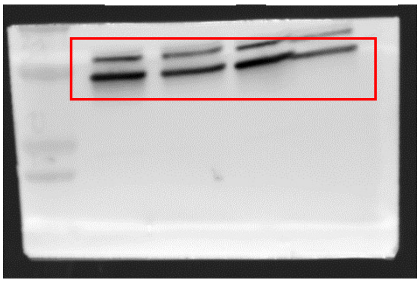

GAPDH MW 36kDa

SCR | CRISPRi ZZZ3#1 | SCR | CRISPRi ZZZ3#3

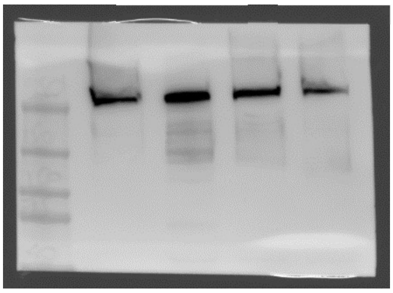

mTOR MW 289kDa

SCR | CRISPRi ZZZ3#1 | SCR | CRISPRi ZZZ3#3

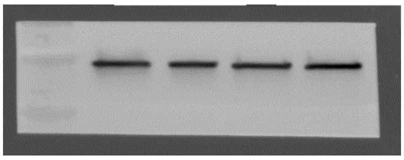

GAPDH MW 36kDa

2° REPLICATE

SCR | CRISPRi ZZZ3#1 | SCR | CRISPRi ZZZ3#3

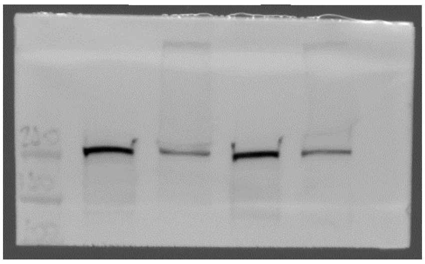

p-mTOR(S2448) MW 289kDa

SCR | CRISPRi ZZZ3#1 | SCR | CRISPRi ZZZ3#3

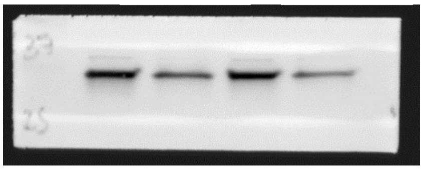

GAPDH MW 36kDa

SCR | CRISPRi ZZZ3#1 | SCR | CRISPRi ZZZ3#3

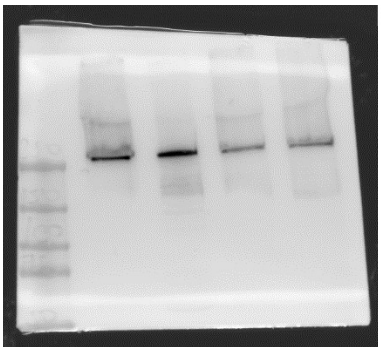

mTOR MW 289kDa

SCR | CRISPRi ZZZ3#1 | SCR | CRISPRi ZZZ3#3

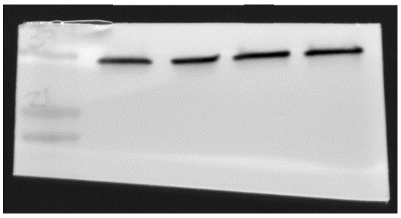

GAPDH MW 36kDa

3° REPLICATE

SCR | CRISPRi ZZZ3#1 | SCR | CRISPRi ZZZ3#3

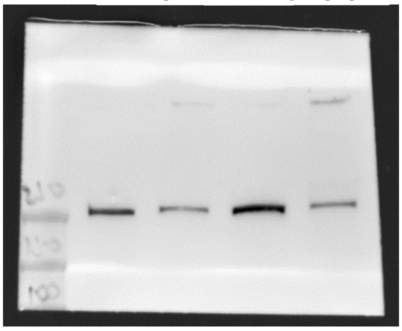

p-mTOR(S2448) MW 289kDa

SCR | CRISPRi ZZZ3#1 | SCR | CRISPRi ZZZ3#3

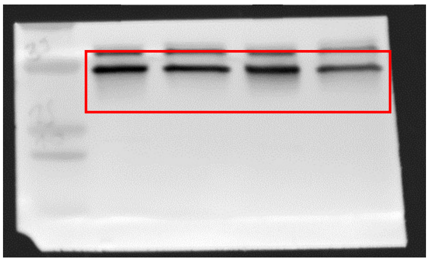

GAPDH MW 36kDa

SCR | CRISPRi ZZZ3#1 | SCR | CRISPRi ZZZ3#3

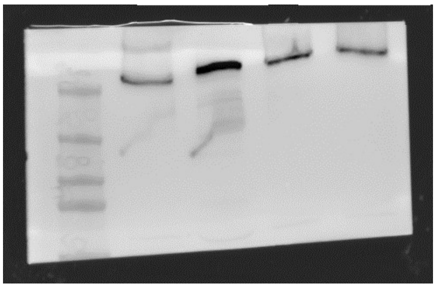

mTOR MW 289kDa

SCR | CRISPRi ZZZ3#1 | SCR | CRISPRi ZZZ3#3

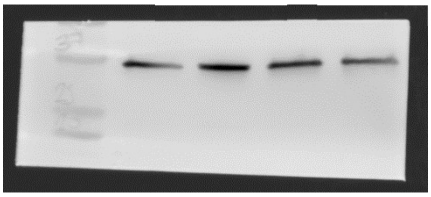

GAPDH MW 36kDa

1° REPLICATE

SCR | CRISPRi ZZZ3#1 | SCR | CRISPRi ZZZ3#3

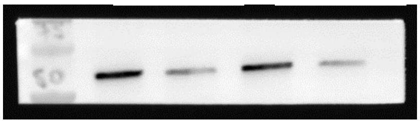

p-AKT (S473) MW 60kDa

SCR | CRISPRi ZZZ3#1 | SCR | CRISPRi ZZZ3#3

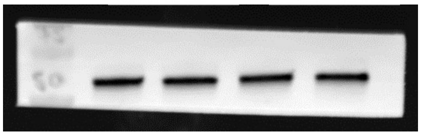

AKT (S473) MW 60kDa

SCR | CRISPRi ZZZ3#1 | SCR | CRISPRi ZZZ3#3

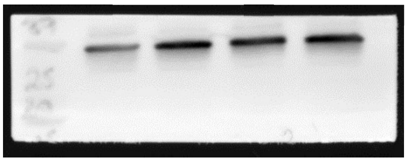

GAPDH MW 36kDa

2° REPLICATE

SCR | CRISPRi ZZZ3#1 | SCR | CRISPRi ZZZ3#3

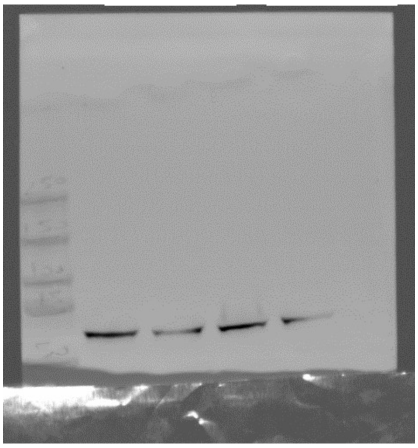

p-AKT (S473) MW 60kDa

SCR | CRISPRi ZZZ3#1 | SCR | CRISPRi ZZZ3#3

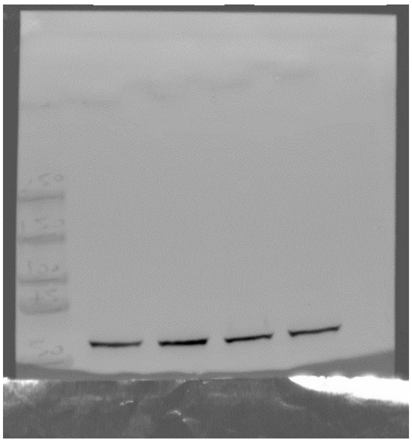

AKT (S473) MW 60kDa

SCR | CRISPRi ZZZ3#1 | SCR | CRISPRi ZZZ3#3

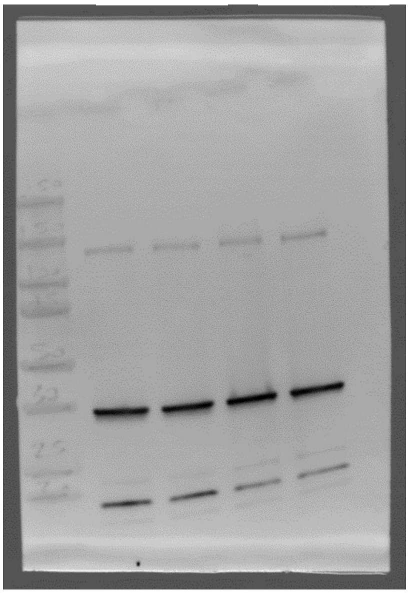

GAPDH MW 36kDa

3° REPLICATE

SCR | CRISPRi ZZZ3#1 | SCR | CRISPRi ZZZ3#3

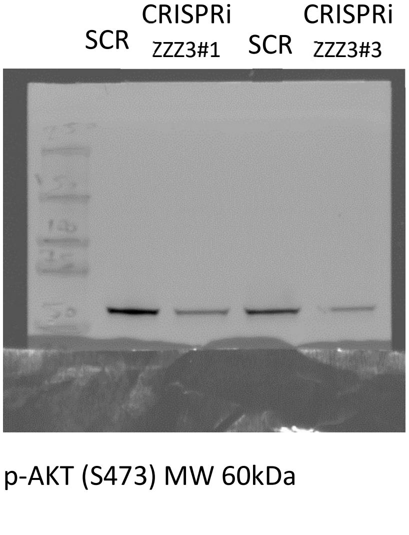

p-AKT (S473) MW 60kDa

SCR | CRISPRi ZZZ3#1 | SCR | CRISPRi ZZZ3#3

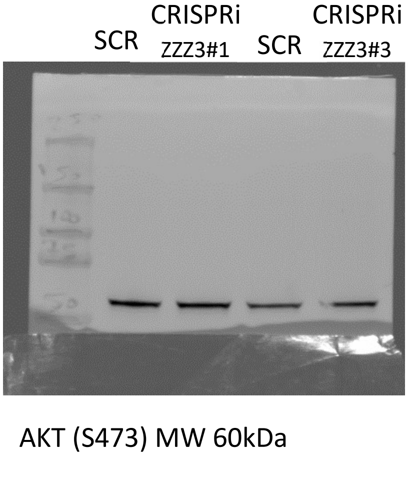

AKT (S473) MW 60kDa

SCR | CRISPRi ZZZ3#1 | SCR | CRISPRi ZZZ3#3

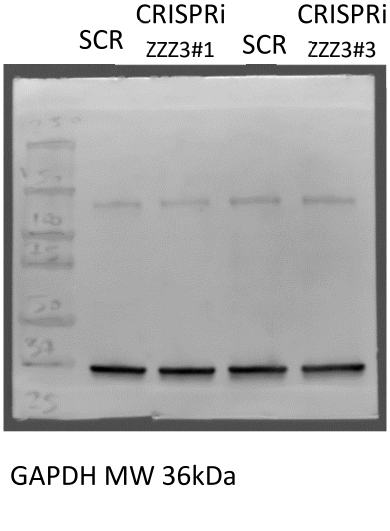

GAPDH MW 36kDa

SUPPLEMENTARY S5B

1° REPLICATE

ESC1

IGF-1 [100nM] SCR - | KD^{ZZZ3} - | SCR + | KD^{ZZZ3} +

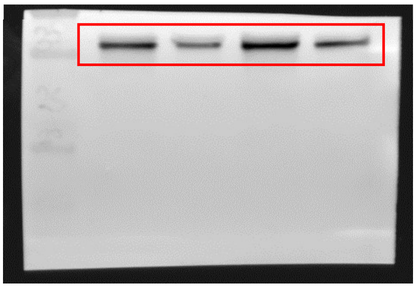

p-ERK1/2 (Thr202/Tyr204) MW 42kDa

ESC1

IGF-1 [100nM] SCR - | KD^{ZZZ3} - | SCR + | KD^{ZZZ3} +

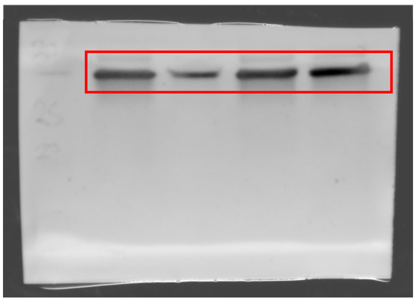

GAPDH MW 36kDa

ESC1

IGF-1 [100nM] SCR - | KD^{ZZZ3} - | SCR + | KD^{ZZZ3} +

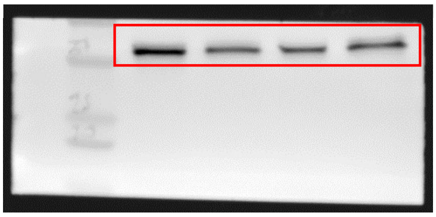

ERK1/2 MW 42kDa

ESC1

IGF-1 [100nM] SCR - | KD^{ZZZ3} - | SCR + | KD^{ZZZ3} +

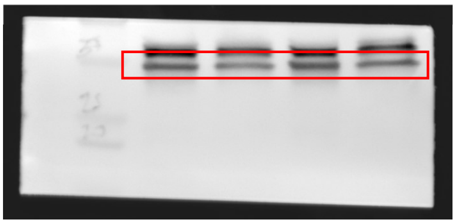

GAPDH MW 36kDa

2° REPLICATE

ESC1

IGF-1 [100nM] SCR - | KD^{ZZZ3} - | SCR + | KD^{ZZZ3} +

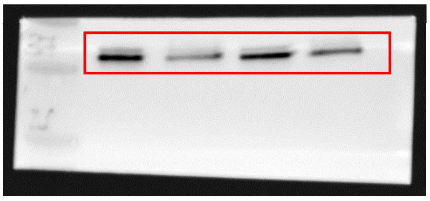

p-ERK1/2 (Thr202/Tyr204) MW 42kDa

ESC1

IGF-1 [100nM] SCR - | KD^{ZZZ3} - | SCR + | KD^{ZZZ3} +

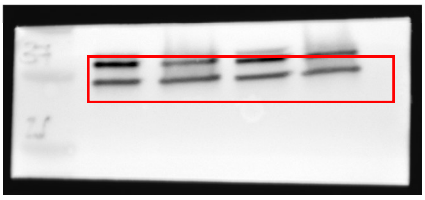

GAPDH MW 36kDa

ESC1

IGF-1 [100nM] SCR - | KD^{ZZZ3} - | SCR + | KD^{ZZZ3} +

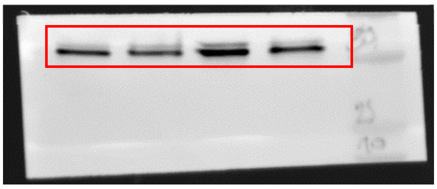

ERK1/2 MW 42kDa

ESC1

IGF-1 [100nM] SCR - | KD^{ZZZ3} - | SCR + | KD^{ZZZ3} +

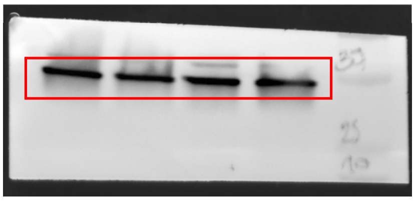

GAPDH MW 36kDa

3° REPLICATE

ESC1

IGF-1 [100nM] | SCR - | KD^{ZZZ3} - | SCR + | KD^{ZZZ3} +

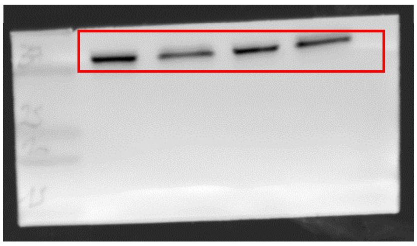

p-ERK1/2 (Thr202/Tyr204) MW 42kDa

ESC1

IGF-1 [100nM] | SCR - | KD^{ZZZ3} - | SCR + | KD^{ZZZ3} +

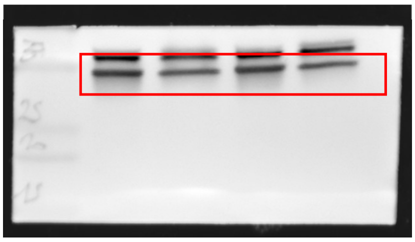

GAPDH MW 36kDa

ESC1

IGF-1 [100nM] | SCR - | KD^{ZZZ3} - | SCR + | KD^{ZZZ3} +

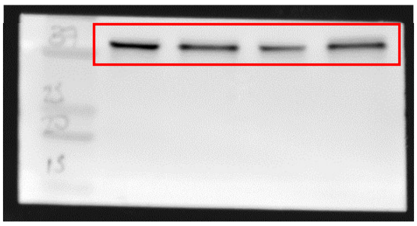

ERK1/2 MW 42kDa

ESC1

IGF-1 [100nM] | SCR - | KD^{ZZZ3} - | SCR + | KD^{ZZZ3} +

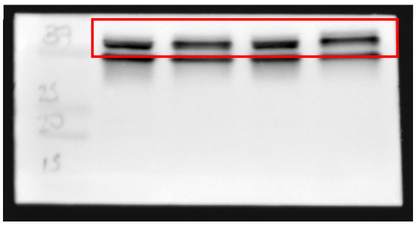

GAPDH MW 36kDa

SUPPLEMENTARY S5C

1° REPLICATE

ESC2

IGF-1 [100nM] | SCR - | KD^{ZZZ3} - | SCR + | KD^{ZZZ3} +

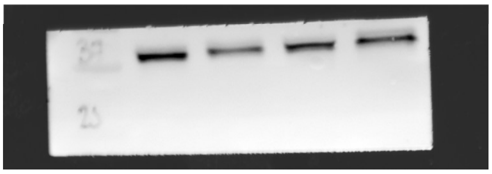

p-ERK1/2 (Thr202/Tyr204) MW 42kDa

ESC2

IGF-1 [100nM] | SCR - | KD^{ZZZ3} - | SCR + | KD^{ZZZ3} +

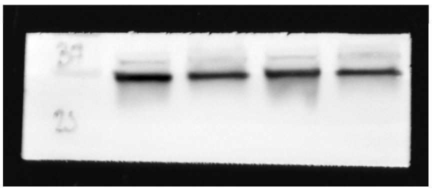

GAPDH MW 36kDa

ESC2

IGF-1 [100nM] | SCR - | KD^{ZZZ3} - | SCR + | KD^{ZZZ3} +

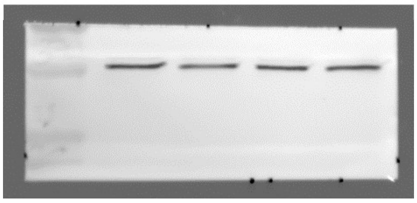

ERK1/2 MW 42kDa

ESC2

IGF-1 [100nM] | SCR - | KD^{ZZZ3} - | SCR + | KD^{ZZZ3} +

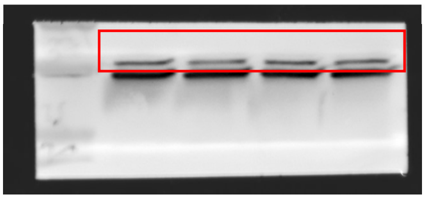

GAPDH MW 36kDa

2° REPLICATE

ESC2

SCR KD^{ZZZ3} SCR KD^{ZZZ3}

IGF-1 [100nM] - - + +

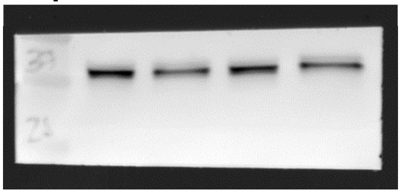

p-ERK1/2 (Thr202/Tyr204) MW 42kDa

ESC2

SCR KD^{ZZZ3} SCR KD^{ZZZ3}

IGF-1 [100nM] - - + +

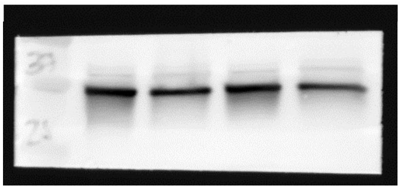

GAPDH MW 36kDa

ESC2

SCR KD^{ZZZ3} SCR KD^{ZZZ3}

IGF-1 [100nM] - - + +

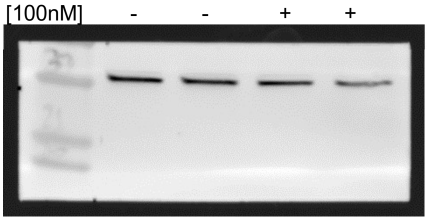

ERK1/2 MW 42kDa

ESC2

SCR KD^{ZZZ3} SCR KD^{ZZZ3}

IGF-1 [100nM] - - + +

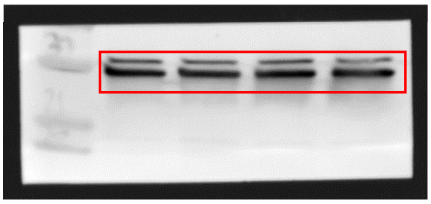

GAPDH MW 36kDa

3° REPLICATE

ESC2

SCR KD^{ZZZ3} SCR KD^{ZZZ3}

IGF-1 [100nM] - - + +

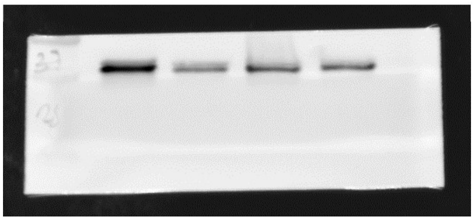

p-ERK1/2 (Thr202/Tyr204) MW 42kDa

ESC2

SCR KD^{ZZZ3} SCR KD^{ZZZ3}

IGF-1 [100nM] - - + +

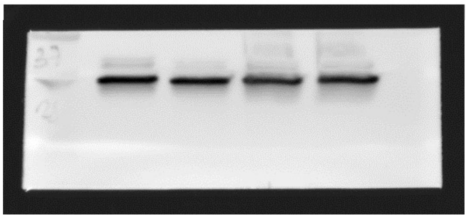

GAPDH MW 36kDa

ESC2

SCR KD^{ZZZ3} SCR KD^{ZZZ3}

IGF-1 [100nM] - - + +

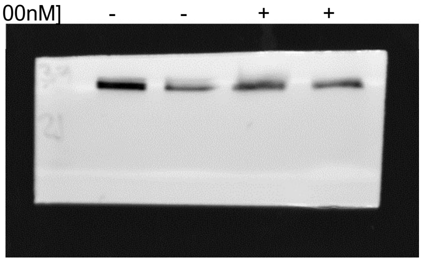

ERK1/2 MW 42kDa

ESC2

SCR KD^{ZZZ3} SCR KD^{ZZZ3}

IGF-1 [100nM] - - + +

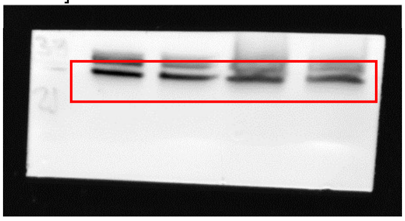

GAPDH MW 36kDa

SUPPLEMENTARY S5D

1° REPLICATE

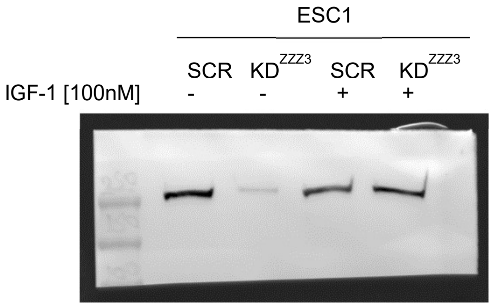

p-mTOR(S2448) MW 289kDa

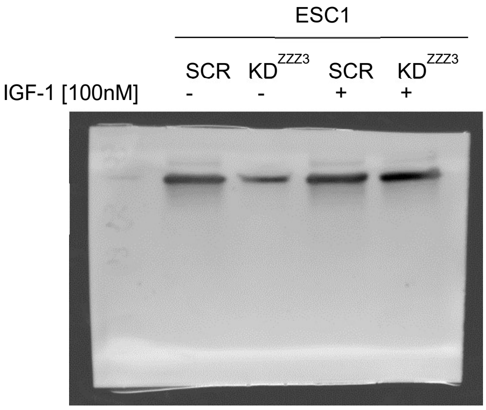

GAPDH MW 36kDa

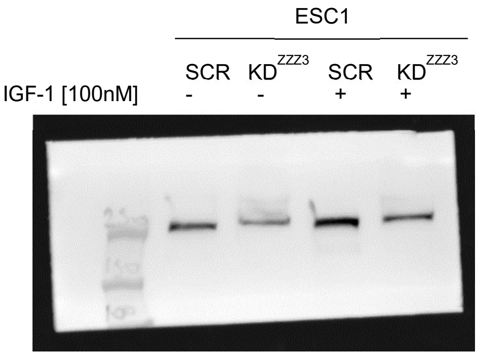

mTOR MW 289kDa

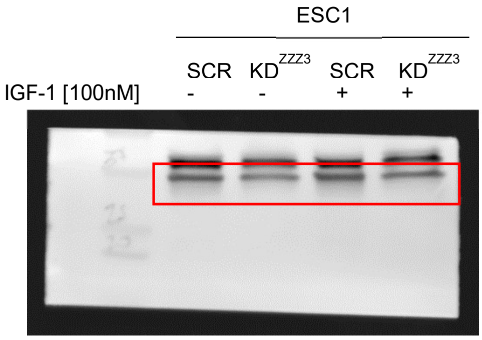

GAPDH MW 36kDa

2° REPLICATE

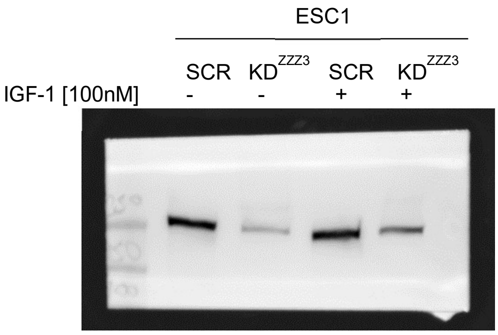

p-mTOR(S2448) MW 289kDa

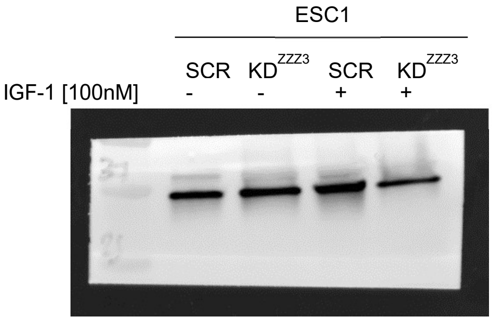

GAPDH MW 36kDa

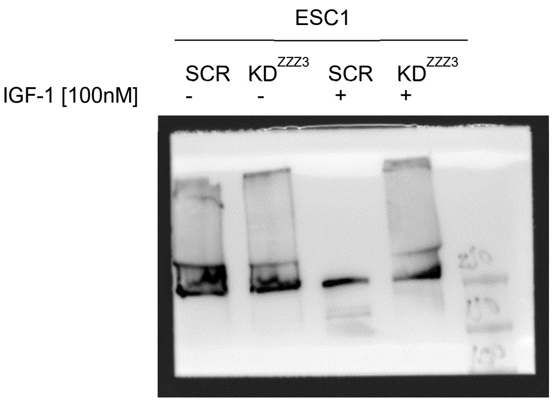

mTOR MW 289kDa

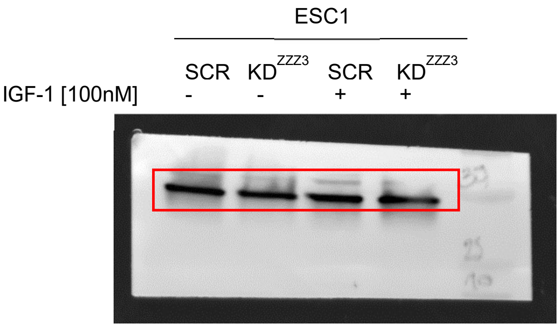

GAPDH MW 36kDa

3° REPLICATE

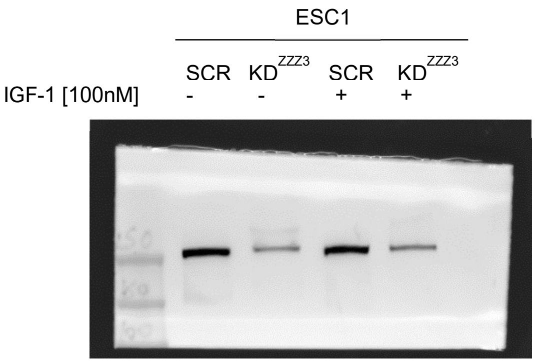

p-mTOR(S2448) MW 289kDa

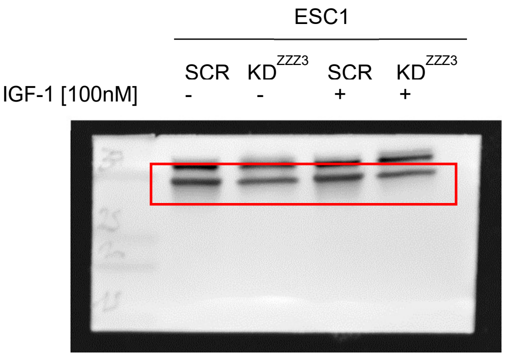

GAPDH MW 36kDa

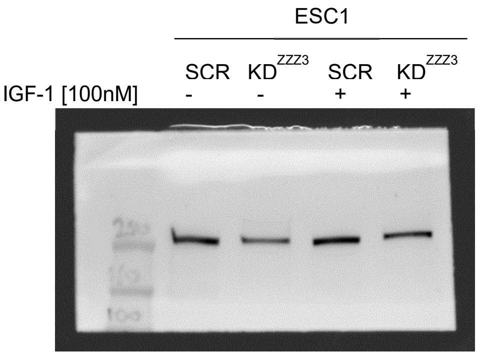

mTOR MW 289kDa

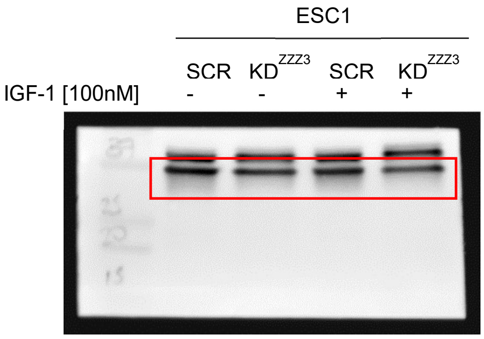

GAPDH MW 36kDa

SUPPLEMENTARY S5E

1° REPLICATE

ESC2

SCR KD^{ZZZ3} SCR KD^{ZZZ3}

IGF-1 [100nM] - - + +

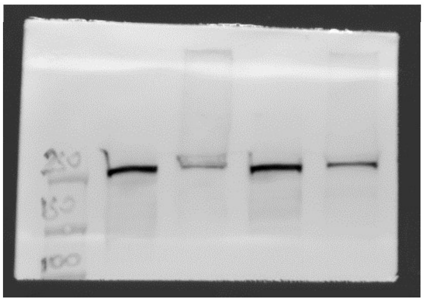

p-mTOR(S2448) MW 289kDa

ESC2

SCR KD^{ZZZ3} SCR KD^{ZZZ3}

IGF-1 [100nM] - - + +

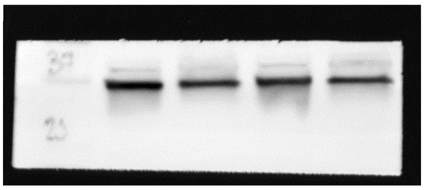

GAPDH MW 36kDa

ESC2

SCR KD^{ZZZ3} SCR KD^{ZZZ3}

IGF-1 [100nM] - - + +

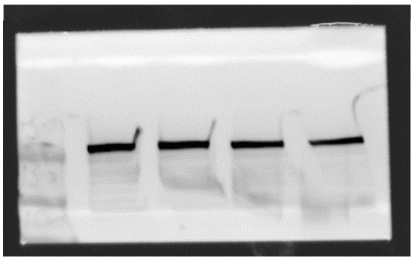

mTOR MW 289kDa

ESC2

SCR KD^{ZZZ3} SCR KD^{ZZZ3}

IGF-1 [100nM] - - + +

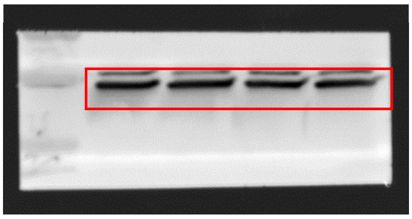

GAPDH MW 36kDa

2° REPLICATE

ESC2

SCR KD^{ZZZ3} SCR KD^{ZZZ3}

IGF-1 [100nM] - - + +

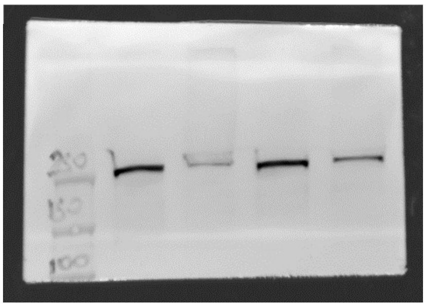

p-mTOR(S2448) MW 289kDa

ESC2

SCR KD^{ZZZ3} SCR KD^{ZZZ3}

IGF-1 [100nM] - - + +

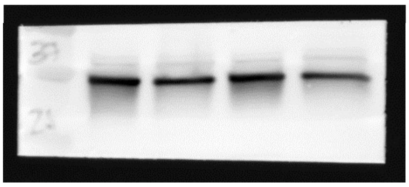

GAPDH MW 36kDa

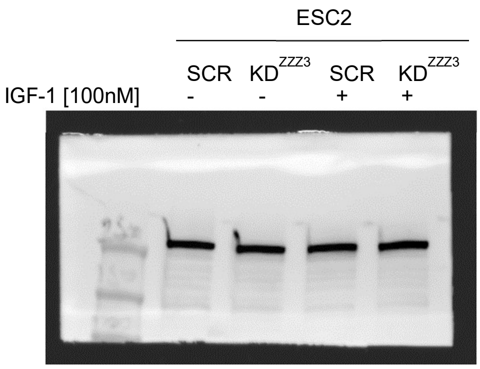

mTOR MW 289kDa

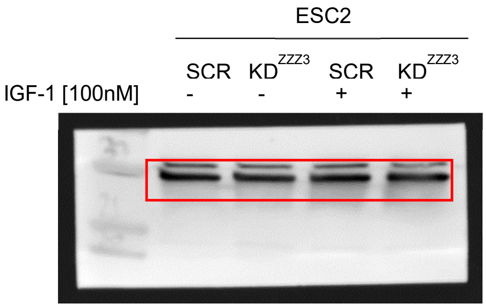

GAPDH MW 36kDa

3°REPLICATE

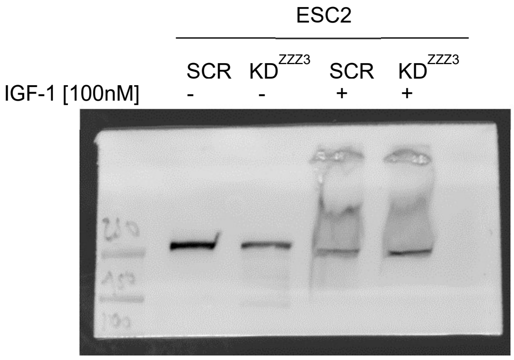

p-mTOR(S2448) MW 289kDa

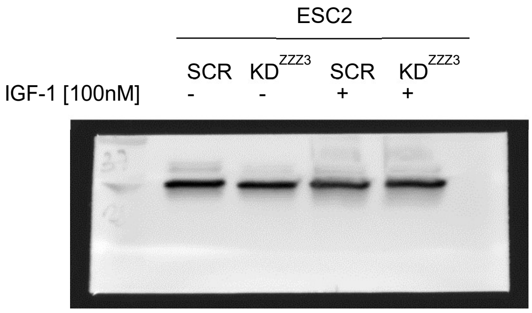

GAPDH MW 36kDa

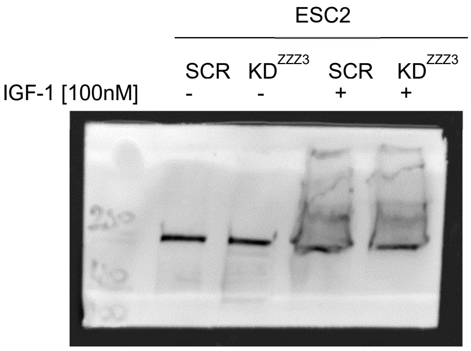

mTOR MW 289kDa

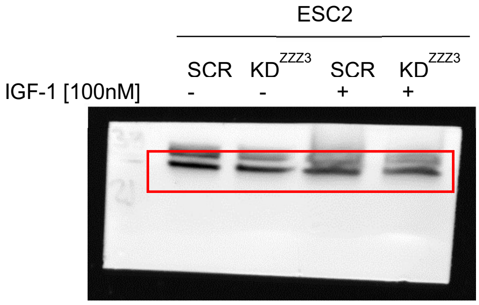

GAPDH MW 36kDa

SUPPLEMENTARY S5F

1° REPLICATE

ESC1

SCR KD^{ZZZ3} SCR KD^{ZZZ3}

IGF-1 [100nM] - - + +

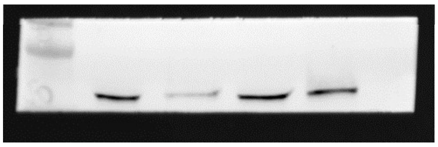

p-AKT (S473) MW 60kDa

ESC1

SCR KD^{ZZZ3} SCR KD^{ZZZ3}

IGF-1 [100nM] - - + +

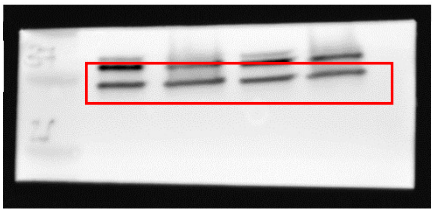

GAPDH MW 36kDa

ESC1

SCR KD^{ZZZ3} SCR KD^{ZZZ3}

IGF-1 [100nM] - - + +

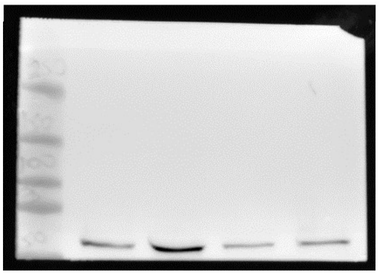

AKT (S473) MW 60kDa

ESC1

SCR KD^{ZZZ3} SCR KD^{ZZZ3}

IGF-1 [100nM] - - + +

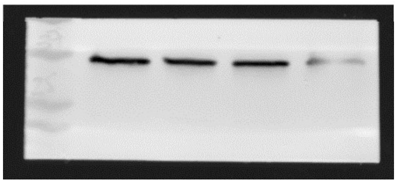

GAPDH MW 36kDa

2°REPLICATE

ESC1

SCR KD^{ZZZ3} SCR KD^{ZZZ3}

IGF-1 [100nM] - - + +

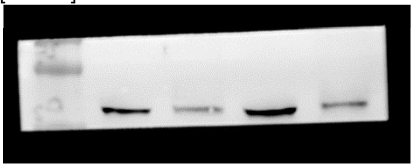

p-AKT (S473) MW 60kDa

ESC1

SCR KD^{ZZZ3} SCR KD^{ZZZ3}

IGF-1 [100nM] - - + +

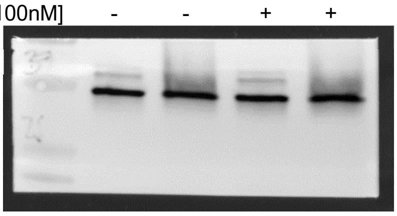

GAPDH MW 36kDa

ESC1

SCR KD^{ZZZ3} SCR KD^{ZZZ3}

IGF-1 [100nM] - - + +

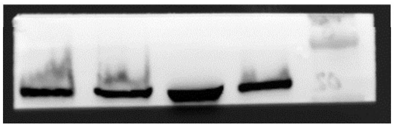

AKT (S473) MW 60kDa

ESC1

SCR KD^{ZZZ3} SCR KD^{ZZZ3}

IGF-1 [100nM] - - + +

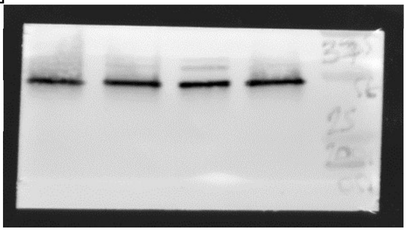

GAPDH MW 36kDa

3° REPLICATE

ESC1

SCR KD^{ZZZ3} SCR KD^{ZZZ3}

IGF-1 [100nM] - - + +

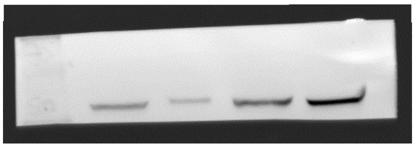

p-AKT (S473) MW 60kDa

ESC1

SCR KD^{ZZZ3} SCR KD^{ZZZ3}

IGF-1 [100nM] - - + +

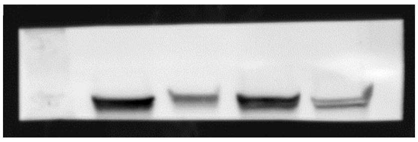

AKT (S473) MW 60kDa

ESC1

SCR KD^{ZZZ3} SCR KD^{ZZZ3}

IGF-1 [100nM] - - + +

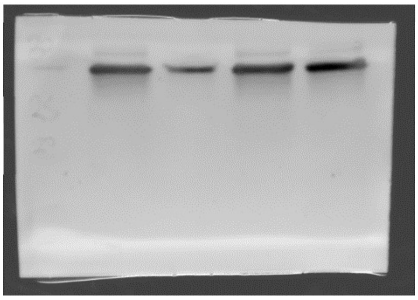

GAPDH MW 36kDa

SUPPLEMENTARY S5G

1° REPLICATE

ESC2

SCR KD^{ZZZ3} SCR KD^{ZZZ3}

IGF-1 [100nM] - - + +

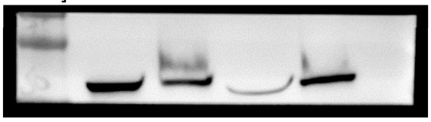

p-AKT (S473) MW 60kDa

ESC2

SCR KD^{ZZZ3} SCR KD^{ZZZ3}

IGF-1 [100nM] - - + +

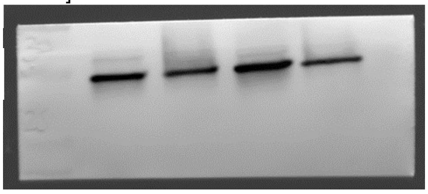

GAPDH MW 36kDa

ESC2

SCR KD^{ZZZ3} SCR KD^{ZZZ3}

IGF-1 [100nM] - - + +

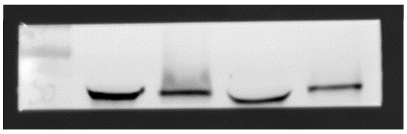

AKT (S473) MW 60kDa

ESC2

SCR KD^{ZZZ3} SCR KD^{ZZZ3}

IGF-1 [100nM] - - + +

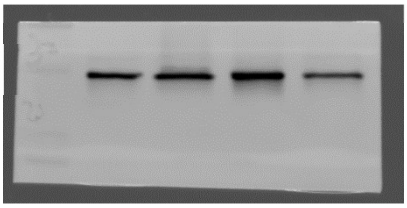

GAPDH MW 36kDa

2° REPLICATE

ESC2

SCR KD^{ZZZ3} SCR KD^{ZZZ3}

IGF-1 [100nM] - - + +

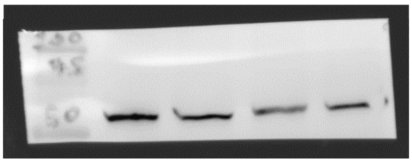

p-AKT (S473) MW 60kDa

ESC2

SCR KD^{ZZZ3} SCR KD^{ZZZ3}

IGF-1 [100nM] - - + +

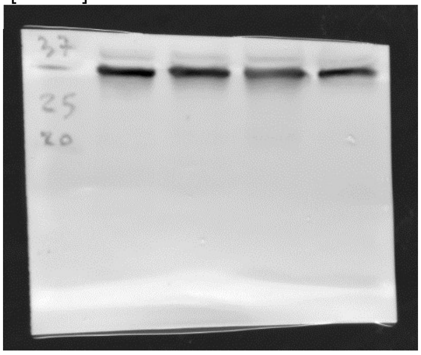

GAPDH MW 36kDa

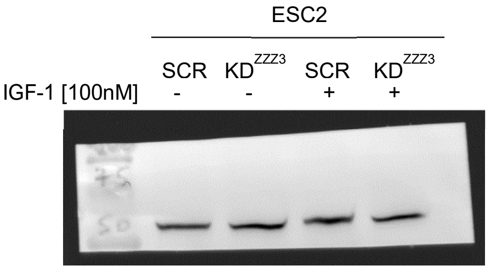

AKT (S473) MW 60kDa

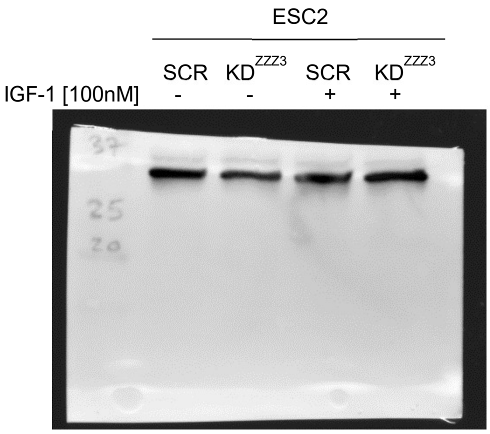

GAPDH MW 36kDa

3° REPLICATE

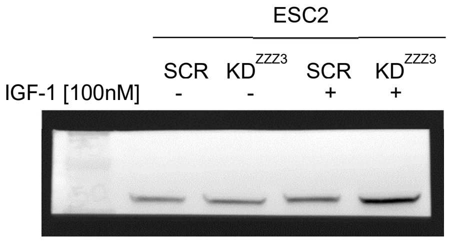

p-AKT (S473) MW 60kDa

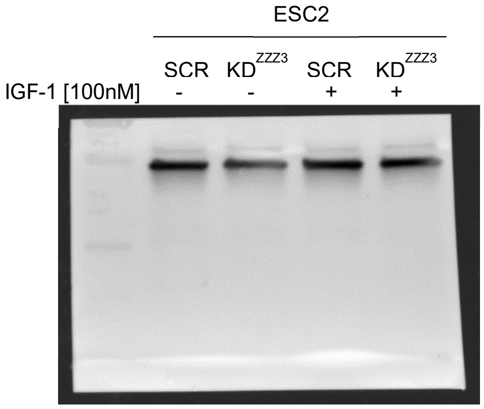

GAPDH MW 36kDa

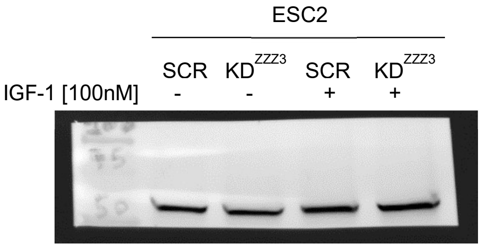

AKT (S473) MW 60kDa

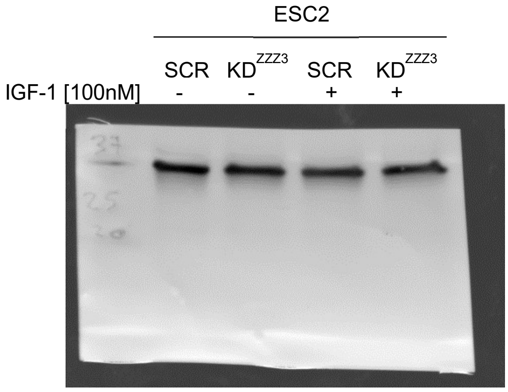

GAPDH MW 36kDa
