# Supplementary material for: Unraveling the impact of ZZZ3 on the mTOR/ribosome pathway in human embryonic stem cells homeostasis
Source: Stem Cell Reports. 2024 May 2;19(5):729–43. doi: 10.1016/j.stemcr.2024.04.002 (PMC11103890; doi:10.1016/j.stemcr.2024.04.002)
Supplement: Document S2. Article plus supplemental information [file mmc5.pdf]

Stem Cell Reports
Article
OPEN ACCESS

Unraveling the impact of ZZZ3 on the mTOR/ribosome pathway in human embryonic stem cells homeostasis

Michela Lo Conte,[1,7] Valeria Lucchino,[1,7] Stefania Scalise,[1] Clara Zannino,[1] Desirèe Valente,[1] Giada Rossignoli,[6] Maria Stella Murfuni,[1] Chiara Cicconetti,[3,4] Luana Scaramuzzino,[1] Danilo Swann Matassa,[5] Anna Procopio,[1] Graziano Martello,[6] Giovanni Cuda,[1,8,*] and Elvira Immacolata Parrotta[2]

[1]Department of Experimental and Clinical Medicine, University Magna Graecia, 88100 Catanzaro, Italy
[2]Department of Medical and Surgical Sciences, University Magna Graecia, 88100 Catanzaro, Italy
[3]Department of Life Sciences and Systems Biology, University of Turin, Via Nizza 52, 10126 Torino, Italy
[4]Italian Institute for Genomic Medicine (IIGM), 10060 Candiolo Torino, Italy
[5]Department of Department of Molecular Medicine and Medical Biotechnology, University of Naples Federico II, 80131 Naples, Italy
[6]Department of Biology (DiBio), University of Padua, Padua, Italy
[7]These authors contributed eqully
[8]Lead contact
*Correspondence: cuda@unicz.it
https://doi.org/10.1016/j.stemcr.2024.04.002

SUMMARY

Embryonic stem cells (ESCs) are defined as stem cells with self-renewing and differentiation capabilities. These unique properties are tightly regulated and controlled by complex genetic and molecular mechanisms, whose understanding is essential for both basic and translational research. A large number of studies have mostly focused on understanding the molecular mechanisms governing pluripotency and differentiation of ESCs, while the regulation of proliferation has received comparably less attention. Here, we investigate the role of ZZZ3 (zinc finger ZZ-type containing 3) in human ESCs homeostasis. We found that knockdown of *ZZZ3* negatively impacts ribosome biogenesis, translation, and mTOR signaling, leading to a significant reduction in cell proliferation. This process occurs without affecting pluripotency, suggesting that ZZZ3-depleted ESCs enter a "dormant-like" state and that proliferation and pluripotency can be uncoupled also in human ESCs.

INTRODUCTION

Embryonic stem cells (ESCs) are characterized by their indefinite self-renewal and pluripotency, allowing differentiation into the three germ layers (Martin, 1981; Sheridan et al., 2012). This dynamic nature stems from large fluctuations in gene expression, shaping a diverse protein landscape (Gabut et al., 2020). While extensive research has focused on transcriptional networks governing ESC pluripotency (Chambers et al., 2007; Masui et al., 2007; Niwa et al., 2000; Tee and Reinberg, 2014), limited understanding exists regarding proliferation regulation. In mouse ESCs, pluripotency and proliferation can be independent processes, meaning that it is possible for mouse ESCs to maintain their pluripotent state without actively proliferating. This separation of pluripotency maintenance and cell division is facilitated by the mammalian target of rapamycin (mTOR) signaling pathway, which plays a pivotal role in orchestrating developmental pausing. Paused blastocysts display diminished mTOR activity, gene expression, and cellular proliferation while maintaining pluripotency (Bulut-Karslioglu et al., 2016). Additionally, genetic inactivation of mTORC1-specific protein raptor reduces proliferation without impacting pluripotency (Xu et al., 2022). Embryonic development arrest induces diapause, a reversible biosynthetic dormant state involving the Wnt/β-catenin signaling and Esrrb pathway (Carbognin et al., 2023; Fan et al., 2020). Cell growth and proliferation entail complex biosynthetic processes, including ribosome biogenesis and translation. The zinc finger ZZ-type containing 3, also known as ZZZ3, acts as a histone H3 reader within the ATAC (Ada2a-containing) complex. Previous studies have demonstrated its role in regulating ribosomal genes in both mouse embryonic stem cells (mESCs) and human adenocarcinoma cells (Fischer et al., 2021; Mi et al., 2018). Our study investigates the role of ZZZ3 in human embryonic stem cells (hESCs), independent of its association with the ATAC complex. Utilizing a combination of interactome, transcriptome, and cellular analyses, we demonstrated that ZZZ3 regulates hESCs proliferation by modulating the mTOR/ribosome biogenesis pathway. Intriguingly, *ZZZ3* knockdown (KD) led to a notable decrease in hESCs proliferation, while maintaining pluripotency and differentiation potential. This observation suggests the potential existence of a pluripotent state marked by biosynthetic quiescence occurring *in vitro* within hESCs.

RESULTS

ZZZ3 interacts with ribosomal proteins and co-localizes in the nucleolus with fibrillarin

To characterize the ZZZ3 biological network in hESCs, we conducted a co-immunoprecipitation (IP) assay using an

This is an open access article under the CC BY license (http://creativecommons.org/licenses/by/4.0/).

anti-ZZZ3 antibody. The co-precipitated proteins were subsequently identified through nanoscale liquid chromatography coupled to tandem mass spectrometry (nanoLC-MS/MS). Experiments were conducted using two lines of hESCs, WA17 and RUES, denoted herein as ESC-1 and ESC-2, respectively. Through the application of the Perseus software for statistical analysis, we identified 474 proteins that co-purified with ZZZ3 in ESC-1 and 744 proteins co-purified with ZZZ3 in ESC-2. Candidate ZZZ3 interactors were chosen based on statistically significant variances (p value <0.05 corrected using the Benjamini-Hochberg procedure) and fold change (FC) values of at least 2.5 compared to samples immunoprecipitated with control IgG. Gene Ontology (GO) enrichment analysis highlighted several significantly overrepresented annotations for biological processes linked to ribosome biogenesis, rRNA processing, rRNA metabolism, RNP complex biogenesis, RNP complex assembly, and cytoplasmic translation (Figure 1A). In the Cellular Component GO hierarchy, the small subunit processome, spliceosome complex, and ribosome emerged as the most prominent terms. Meanwhile, in the molecular function category, enrichment was observed in terms including structural constituent of ribosome, rRNA binding, snRNA binding, and helicase activity (Figures S1A and S1B; Table S_GO). Furthermore, analysis of protein-protein interaction (PPI) networks among proteins associated with ZZZ3 (FC >10), alongside the enrichment analysis of biological processes characterized by elevated scores, enabled the identification of numerous ribosomal protein components. Specifically, these included constituents of the 40S subunit such as RPS8, RPS15, and RPS15A, as well as components of the 60S subunit such as RPL27, RPL27A, and RPL36 (Figure 1B). The principal biological processes enriched by ZZZ3-interacting proteins are depicted in the PPI network (Figure 1C). Given that ribosome biogenesis primarily occurs in the cell's nucleolus, we employed immunostaining to identify the co-expression of ZZZ3 and fibrillarin (FBL) in wild-type ESC-1 and -2. FBL is a nucleolar protein actively engaged in pre-rRNA processing, pre-rRNA methylation, and ribosome assembly (Rodriguez-Corona et al., 2015; Tollervey et al., 1993). The intensity-based correlation analysis, performed utilizing the Pearson's correlation coefficient, revealed strong co-localization of ZZZ3 and FBL within the nucleolus of hESCs (Figure S1C). Consistent with prior research associating ZZZ3 with the regulation of ribosomal gene expression (Fischer et al., 2021; Mi et al., 2018), we found that many ZZZ3 interactors are indeed ribosomal proteins and that ZZZ3 co-localized with FBL in the nucleolus. Validation of the interaction between ZZZ3 and co-precipitated proteins was accomplished via IP followed by western blot analysis, as illustrated in Figure S1D.

Pluripotency and differentiation remain uncompromised upon ZZZ3 deficiency

Pluripotent ESCs exhibit high proliferative activity, necessitating rapid ribosome biogenesis to sustain their growth and proliferation. As ZZZ3 interacts with and potentially regulates genes encoding ribosomal proteins, we aimed to probe the effect of ZZZ3 depletion on hESCs' pluripotency. To address this, we employed stable KD of ZZZ3 expression in ESC-1 and ESC-2 lines using piggyBac (PB) vectors (Figure S2A). Three distinct PB short hairpin RNAs (PB-shRNAs) were employed independently to achieve silencing of *ZZZ3*; shRNA#2 was selected for subsequent experiments due to its high KD efficiency in both ESC lines (Figure S2B). Furthermore, we generated ZZZ3 KD hESCs using a CRISPR-interference (CRISPRi) PB vector as an alternative method for *ZZZ3* silencing, thus providing an orthogonal system (Figures S2C and S2D). Initially, we evaluated the expression of alkaline phosphatase (AP) to assess the proportion of undifferentiated cells between ZZZ3 KD and SCR control ESCs. We observed no discernible differences in either AP activity or cell morphology between ZZZ3 KD ESCs and control cells (Figure 2A). Pluripotency is stabilized by a triad of interconnected pluripotency transcription factors, namely OCT4, SOX2, and NANOG, that cooperatively regulate gene expression (Li and Izpisua Belmonte, 2018; Ng and Surani, 2011). Quantitative immunoblotting and immunostaining analyses confirmed the sustained expression of OCT4, NANOG, and SOX2 proteins in ZZZ3 KD ESCs (Figures 2B–2E). Gene expression analysis via RNA sequencing (RNA-seq) of a panel of pluripotency regulator genes, including *OCT4*, *SOX2*, *NANOG*, *MYC*, *LIN28A*, *LIN28B*, *BCOR*, *FOXO1*, *DPPA2*, *DPPA4*, *UTF1*, and *KLF7*, revealed a comparable expression pattern between modified and control ESCs (Figure 2F). Furthermore, the expression levels of *OCT4*, *NANOG*, and *SOX2* mRNA were assessed via quantitative reverse-transcription polymerase chain reaction in ZZZ3 KD ESCs. Once more, comparative analysis revealed no discernible differences in mRNA expression between the control (SCR) and ZZZ3 KD ESCs (Figure 2G). Immunoblot analysis was conducted to examine the expression levels of OCT4, NANOG, and SOX2 in ESCs transfected with CRISPRi-ZZZ3. Remarkably, no significant differences were detected in their relative expression compared to control cells (Figures 2H and 2I). To investigate further in this direction, as additional means for pluripotency, we analyzed the capacity of ZZZ3 KD hESCs to form embryoid bodies (EBs) and differentiate properly. We focused on early steps of differentiation such as the emergence of the three germ layers using a commercial kit (R&D Systems). Both SCR and ZZZ3 KD ESCs resulted comparably positive for SOX17, OTX2, and BRACHYURY as shown by quantitative immunofluorescence analysis (Figure S3A). Additionally, gene expression analysis by

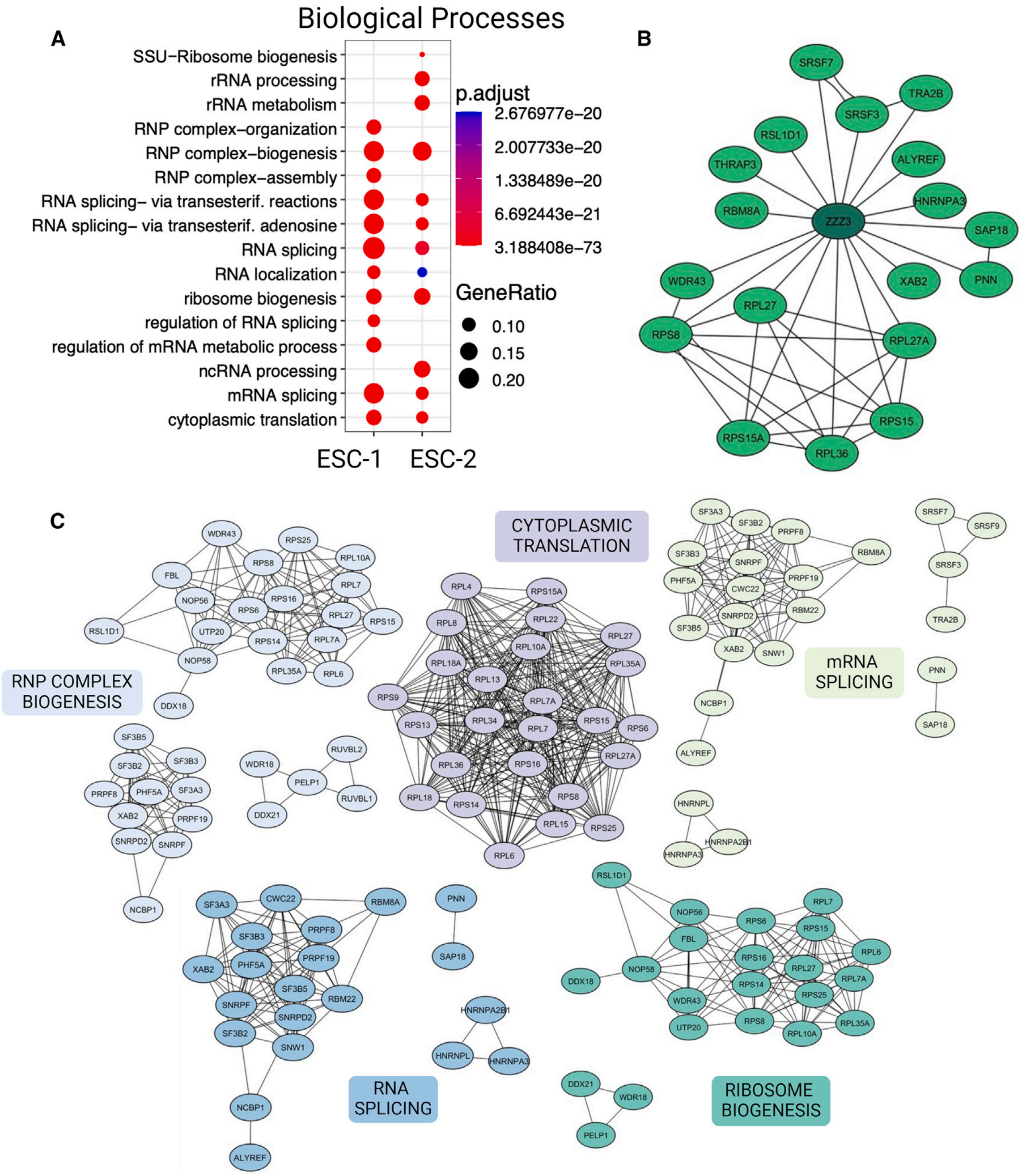

Figure 1. ZZZ3 interacts with proteins involved in cytoplasmic translation, ribosome biogenesis, RNP complex biogenesis, and RNA processing

(A) Gene ontology (GO) enrichment analysis was performed on genes selected based on their significance (p value <0.05, corrected using the Benjamini-Hochberg procedure) and fold change (FC $\geq$ 2.5). The analysis was conducted in R, utilizing the Bioconductor package. The enriched biological processes (BPs) associated with the selected genes are presented.

(B) The protein-protein interaction (PPI) network illustrates the relationships among the top differentially expressed genes (DEGs) with FC $\geq$ 10. This network highlights DEGs enriching the BP with the highest gene ratio in ESC-1 and ESC-2. Edges in the network represent known interactions between DEGs and ZZZ3.

(C) PPI networks were constructed for all members of the top five BPs with the highest gene ratio in ESC-1 and ESC-2. The networks were generated using the Cytoscape software (version 3.10.0).

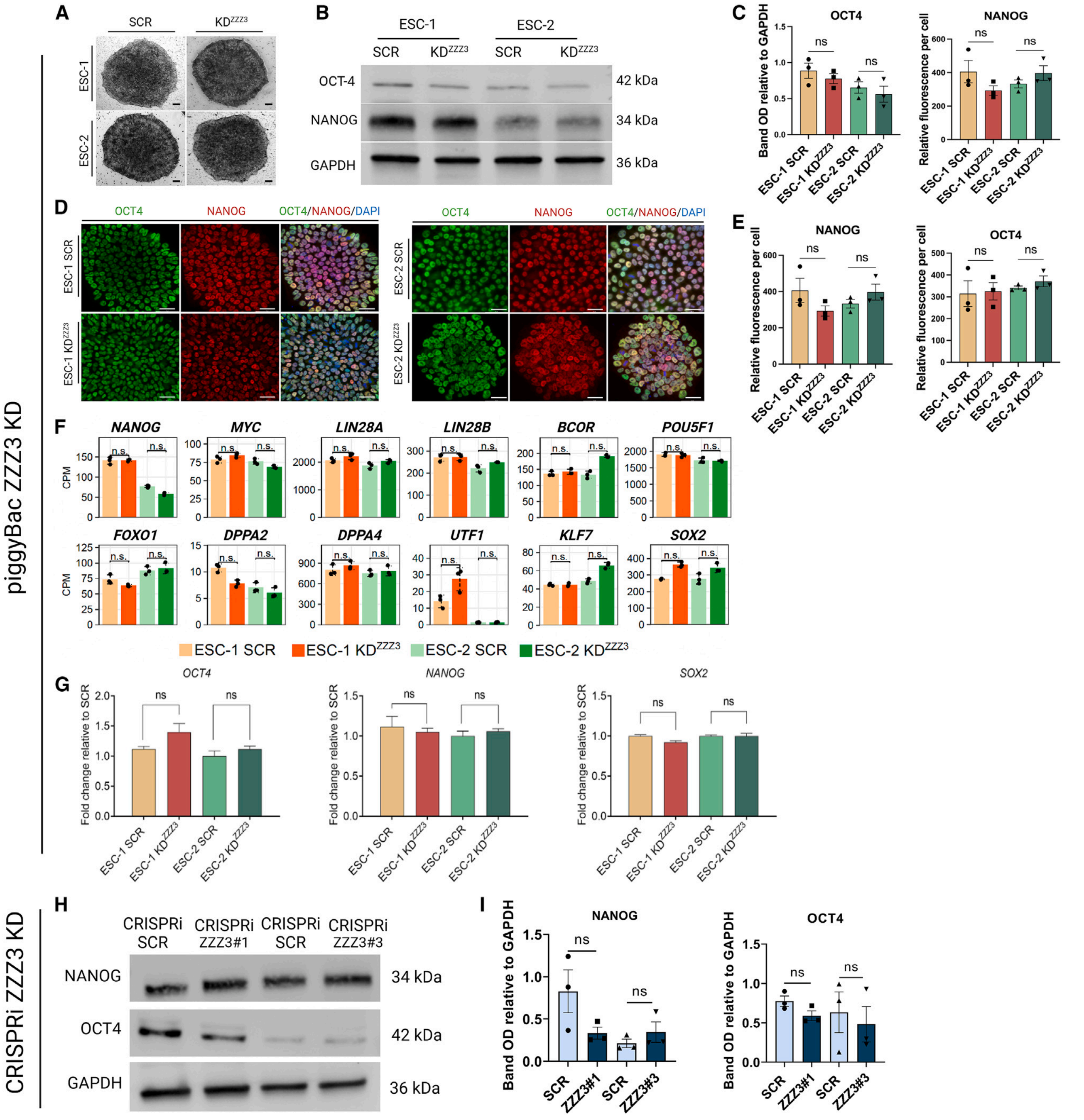

Figure 2. Pluripotency and differentiation are kept intact upon ZZZ3 KD

(A) Alkaline phosphatase staining of SCR control and ZZZ3 KD ESCs. Scale bar 200 μm.

(B) Representative western blot images of the OCT4 and NANOG expression and relative quantification (C).

(D) Representative fluorescence microscopy images of SCR control and ZZZ3 KD hESCs for OCT4 and NANOG. Nuclei were counterstained using DAPI. Scale bar, 50 μm.

(E) Quantification of fluorescence intensity using ImageJ software.

(F) Barplots show the gene expression levels of a panel of pluripotency regulators in SCR and ZZZ3 KD hESCs as determined by RNA-seq analysis. Mean expression levels ± SEM from three independent experiments is represented by bars, with individual data points overlaid as dots. Statistical analysis was performed using ANOVA, $p > 0.05$.

(legend continued on next page)

RNA-seq of a panel of three germ layer genes unveiled a consistent expression pattern in both SCR and ZZZ3 KD ESCs (Figure S3B). Finally, we extended the analysis assessing the expression changes for specific target genes (*PDGFRA*, *SOX9*, and *S100B* for ectoderm, *PECAM*, *ACTA*, and *HAND1* for mesoderm, and *DLX5*, *HTATSF1*, and *GATA6* for endoderm) via qPCR on EBs at day 10 of differentiation (Figure S3C). Additionally, we assessed the differentiation capabilities of hESCs following ZZZ3 CRISPRi-mediated silencing, employing it as an orthogonal system. Specifically, we examined the emergence of the three germ layers by immunostaining the cells with antibodies targeting SOX17, OTX2, and BRACHYURY in both SCR and CRISPR-ZZZ ESCs (Figure S3D). Once more, our observations revealed no significant alterations in the expression patterns of the analyzed markers. These assays allowed us to conclude that hESCs with ZZZ3 KD can undergo differentiation in a manner similar to that of control cells providing additional confirmation that depleting ZZZ3 does not impair pluripotency.

KD of ZZZ3 induces a “dormant-like” pluripotent status

Pluripotency and proliferation are two processes in mESCs that can be uncoupled. Indeed, during diapause, pluripotency is kept intact although proliferation is strongly reduced. Because ZZZ3 interacts with ribosomal proteins and might be involved in the regulation of ribosome biogenesis, which occurs synergistically with cell growth and proliferation, we further wondered what was the effect of ZZZ3 KD on ESCs proliferation. Cell proliferation analysis, performed by cell counting at specific time points (24, 48, 72, and 96 h) and MTT assay, revealed that ZZZ3-depleted cells were significantly less proliferative compared with control ESCs (Figures 3A and 3B). The size of EBs correlates with proliferative capacity of ESCs, and cells with faster cell cycle progression are more likely to contribute to larger EBs (Hwang et al., 2009). We measured the diameter of EBs generated from ZZZ3 KD and SCR control hESCs and observed that modified cells generated smaller EBs (Figure 3C). Next, we performed immunostaining for Ki67, a protein involved in the cell cycle regulation, thus serving as a cellular marker for cell proliferation (Bullwinkel et al., 2006; Sun et al., 2017). We found that the expression of Ki67 is dramatically reduced in ZZZ3 KD hESCs (Figure 3D). Additionally, the expression of c-MYC and E2F4a, markers of cell proliferation tested via immunoblot, resulted in a significant decrease in silenced cells (Figure 3E). To investigate deeper in this direction, following synchronization of the cell cycle in the G2/M phase with nocodazole, we performed flow cytometry to assess the distribution of cells across the G1, S, and G2 phases in ZZZ3 KD hESCs compared to SCR controls. Cells with ZZZ3 KD showed a delay in cell cycle progression, particularly at the G2/M phase (Figure 3F). In the attempt to rescue the proliferation defect, we conducted cell cycle analysis using ZZZ3 KD hESCs generated with a PB vector encoding for doxycycline-inducible ZZZ3 KD (TET-shZZZ3) (Figures S2E and S2F). Ninety-six hours (96 h) after doxycycline withdrawal and ZZZ3 re-upregulation, cells exhibit enhanced progression through the cell cycle, transitioning more readily from the G2/M phase at T0 (synchronized) to the S and G1 phases (Figure 3G), suggesting that the “dormant status” observed in ZZZ3 KD hESCs can be rescued through the re-upregulation of ZZZ3.

ZZZ3 is associated with ribosome biogenesis and translation

To gain insight into the functional and regulatory roles of ZZZ3 in hESCs, we employed RNA-seq analysis on ZZZ3 KD ESCs. As a first step, we performed a dimensionality reduction procedure that evidenced how a good fraction of the transcriptomic variability is connected with the ESCs identity (Figure 4A). Nevertheless, the second principal component separated the samples according to the ZZZ3 state, suggesting a common effect of the KD on the ESCs. We took into account this ESCs intrinsic variability by performing a differential expression analysis with a paired design (for details see experimental procedures section), thus identifying the genes whose expression was affected by the ZZZ3 KD in both lines of hESCs. Analysis of RNA-seq data comparing SCR hESCs to ZZZ3 KD hESCs revealed significant alterations in gene expression. Specifically, ZZZ3 deficiency was associated with an increase in the expression of 259 genes and a decrease in the expression of 275 genes (FC ≥ 2 a false discovery rate < 0.05) (Figures 4B and 4C; Table S2). Notably, many of the genes downregulated in ZZZ3 KD hESCs encode for small and large ribosomal proteins (Figure 4D). Gene set enrichment analysis (GSEA) revealed that many of the downregulated genes were linked to biological processes including post-transcriptional regulation of gene expression, translational initiation, RNA processing, ribosome biogenesis, ribonucleoprotein complex biogenesis, and cytoplasmic translation (Figure 4E). Taken together, the results of both interactome and transcriptome analyses suggest that

(G) qRT-PCR analysis of the core pluripotency factors OCT4, NANOG, and SOX2.
(H) Western blot image showing the expression of NANOG and OCT4 in SCR control ZZZ3 and KD hESCs generated via CRISPRi system and relative quantification (I). Data are presented as mean ± SEM from 3 independent experiments. Statistical significance was determined by t test, ns = $p > 0.05$. GAPDH was used as loading control. Full-length blots are shown in File S1.

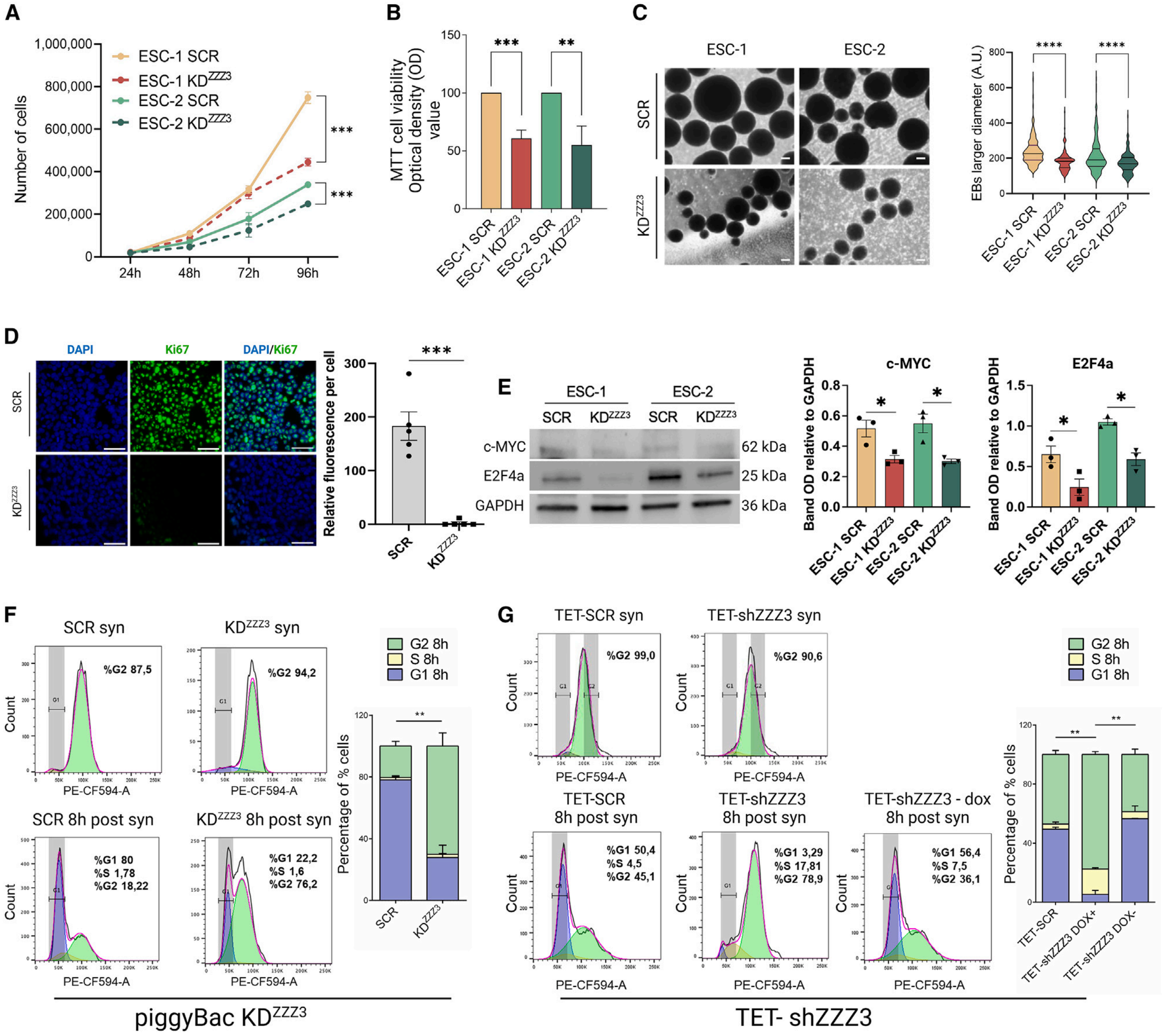

Figure 3. ZZZ3 knockdown in ESCs results in reduced proliferation

(A) Cell proliferation was monitored over a 96-h time course from 3 independent experiments. The initial cell density was 3 × 10ˆ4 cells/well.

(B) OD at 570 nm was measured to determine MTT reduction in ZZZ3 KD ESCs.

(C) Phase-contrast images depict EBs generated from SCR and ZZZ3 KD ESC-1 and ESC-2. Measurement of EB size reveals a reduced diameter of EBs from ZZZ3 KD ESCs. Scale bar 200 μm. 150 EBs per line were measured as shown in violin plots.

(D) Representative images depict immunostaining of the proliferation marker Ki67, showcasing a significant reduction in ZZZ3 KD cells. Nuclear DNA was counterstained with DAPI (blue). Scale bar, 50 μm. Relative fluorescence per cell measured for Ki67 immunostaining in ZZZ3 KD and control cells is shown to the right. At least 200 nuclei were analyzed per cell line.

(E) Western blot images illustrate the expression of c-MYC and E2F4a in SCR control and ZZZ3 KD hESCs and relative OD quantification. GAPDH was used as a loading control. Full-length blots are shown in File S1.

(F) Cell cycle profiles were measured by flow cytometry using propidium iodide. The analysis was conducted on SCR and ZZZ3 KD hESCs synchronized in the G2/M phase using nocodazole (upper panels) and at 8 h post-nocodazole withdrawal (lower panels). Histograms show the distribution of cells across the G0/G1, S, and G2/M phases. Quantitative analysis highlights a significant increase in G2/M phase of ZZZ3 KD cells.

(G) Cell cycle profile of Tet-SCR and TET-shZZZ3 hESCs (Doxycycline [dox]-induced depletion of ZZZ3). Cells were synchronized in G2/M phase with nocodazole for 12 h. Subsequently, the cell cycle analysis was performed immediately after synchronization (syn) and after 8h

(legend continued on next page)

ZZZ3 not only regulates the transcription of ribosomal genes but also interacts with ribosomal proteins, indicating a crucial role for ZZZ3 in the ribosome biogenesis process.

ZZZ3 KD impairs ribosome biogenesis and translation and triggers p53 activation

Ribosome biogenesis is a well-orchestrated process involving the coordinated actions of three types of RNA polymerase (RNAP I, RNAP II, and RNAP III) that make up the active ribosomes, machinery of translation (Jiao et al., 2023). GSEA analysis of transcriptomic data revealed that rRNA processing (normalized enriched score [NES] = −5.09), translation (NES = −6.7), and ribosome (NES = −7.08) are some of the most downregulated biological processes in ZZZ3 KD cells (Figure 5A). Polysome profiling analysis was employed to measure the translation dynamics in ZZZ3 KD ESCs. This assay revealed a clear reduction in the 80S and polysomes fractions in cells with depletion of ZZZ3 compared to SCR ESCs (Figure 5B). Immunoblot analysis for RPL19a and RPS6 was used to validate protein distribution in polysome profiling subfractions (1–12) (Figure 5C). rRNA synthesis and ribosome biogenesis primarily occur within the nucleolus and an impairment of these processes can lead to changes in the size of this subcellular structure. Notably, our research revealed that ZZZ3 KD hESCs exhibit a modified nucleolar structure, characterized by reduced size (Figure 5D). This alteration potentially reflects disturbances in ribosome biogenesis. Moreover, an impaired ribosome biogenesis can trigger nucleolar stress via a p53-dependent mechanism (Gilkes et al., 2006). We measured the expression level of p53 and its direct target p21 using immunoblot and immunoassay, respectively. Both were dramatically increased in ZZZ3 KD ESCs (Figures 5E and 5F) indicative of a stress condition taking place. Interestingly, the increase in p53 and p21 levels in ZZZ3 KD hESCs, along with the delay in cell cycle progression, prompts us to investigate whether apoptosis was active. Analysis via western blot of critical apoptotic markers, namely CASPASE-3 and cleaved CASPASE-9, along with qPCR evaluation of *BIM*, *BAX*, and *FAS* genes, revealed no notable variances between ZZZ3 KD hESCs and the control cells (Figures S4A and S4B, respectively). Moreover, TUNEL assay analysis did not reveal any noticeable differences between scramble and ZZZ3 KD cells (Figure S4C). Collectively, these outcomes, in conjunction with data from Ki67 immunostaining, cell proliferation, and cell cycle analysis, indicate that the decreased cell proliferation observed in ZZZ3 KD cells is unlikely to be attributed to cell death.

ZZZ3 regulates cell proliferation and translation through the PI3K-AKT-mTOR axis

In the attempt to molecularly connect the reduced cell proliferation and impaired ribosome biogenesis observed in ZZZ3 KD ESCs, we focused on the ERK (extracellular signal-regulated kinase) and mTOR, the latter crucially involved in the regulation of translation initiation in mammalian cells. The PI3K-AKT-mTORC1 signaling pathways were significantly enriched by genes downregulated upon ZZZ3 depletion (Figure 6A). The convergence of signals from the mTOR and PI3K pathways provides an integrated mechanism for cells controlling the rate of protein synthesis and cell growth (Roux et al., 2007). ERK is a member of the mitogen-activated protein kinase family, which plays a central role in cell proliferation. The phosphorylated ERK (p-ERK) serves as an indicator of the activation status of the ERK pathway and positively correlates with the rate of cell proliferation (Morrison, 2012). Immunoblot analysis for total ERK1/2 and p-ERK (Thr202/Tyr204) revealed that their expression is significantly reduced in ESCs with ZZZ3 KD (Figure 6B). The mTOR pathway plays an important role in regulating various cellular processes, including ribosome biogenesis, by modulating processing of rRNA and the translation of ribosomal proteins, as well as activating the RNA polymerase I transcription machinery (Mayer and Grummt, 2006). mTOR activation stimulates protein synthesis primarily through the phosphorylation and regulation of downstream targets, including components of the translation machinery, such as ribosomal protein S6 kinase (S6K1 or p70S6 kinase) (Holz et al., 2005), leading to the phosphorylation of its downstream targets, such as the ribosomal protein S6 (RPS6), a critical step in the initiation of protein translation (Bohlen et al., 2021; Jastrzebski et al., 2007). Immunoblot analysis of the active phosphorylated AKT(Ser473), which activates mTOR by phosphorylation on its Ser2448, confirmed a reduced expression of both in ZZZ3 KD ESCs (Figures 6C and 6D). The main downstream targets of mTOR signaling, phosphorylated P70S6K (T421/424) and phosphorylated RPS6 (Ser235), were also significantly reduced in ESCs with ZZZ3 depletion (Figures 6E and 6F). The inactivation by phosphorylation of 4E-BP1 (eukaryotic initiation factor 4E-binding protein 1) on T37/42 is another key mechanism by which mTOR promotes protein synthesis (Böhm et al., 2021). We found that the inactive form of 4E-BP1 is less expressed in ZZZ3 KD cells (Figure 6G) as well as its downstream

of nocodazole withdrawal (8 h post-syn). Quantitative analysis reveals a significant increase in G2/M phase cells in TET-shZZZ3 cells in the presence of DOX (+DOX). Conversely, TET-shZZZ3 hESCs culture in the absence of doxycycline (-DOX) reverts to a state resembling SCR cell. Data are presented as means ± SEM or SD of 2 or 3 independent experiments and significance was calculated relative to the SCR control using t test, $*p \leq 0.05$, $**p \leq 0.01$, $***p \leq 0.001$, $****p \leq 0.0001$.

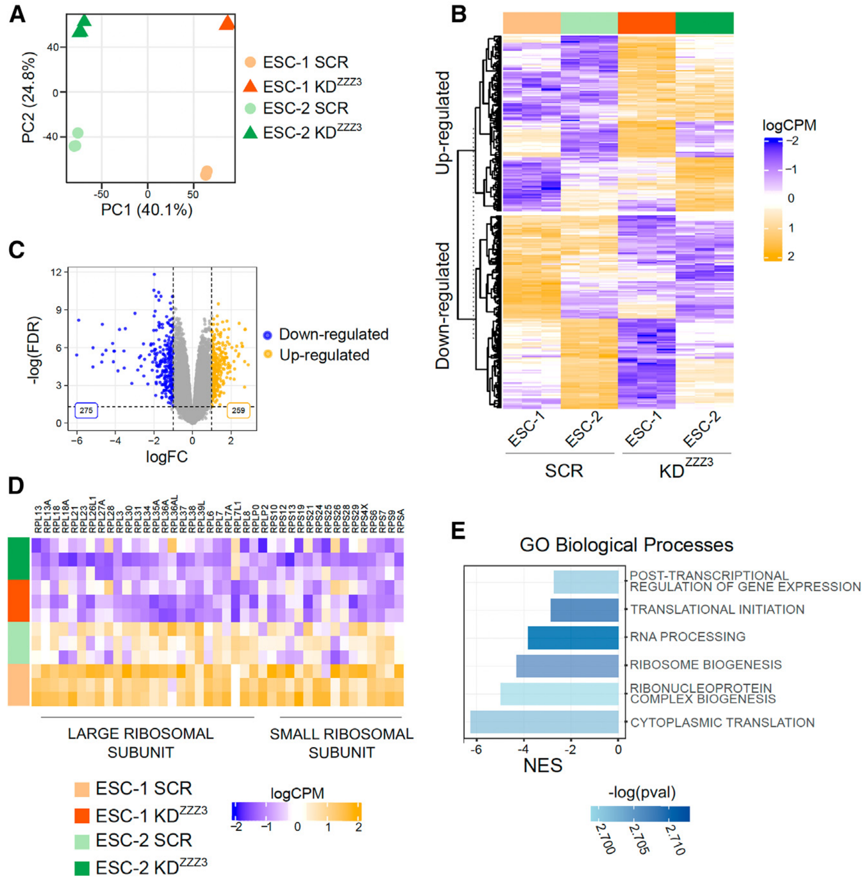

Figure 4. ZZZ3 KD in hESCs alters transcription of genes associated with ribosome biogenesis and translation

(A) PCA of the logCPM gene expression data.

(B) Heatmap of the DEGs between ZZZ3 KD ESCs and SCR condition (*Z* score scaled logCPM) identified with thresholds by applying the following thresholds: |logFC| > 1 and adjusted *p* value <0.05. DEGs are clustered according to a hierarchical clustering procedure based on Euclidean distance.

(C) Volcano plot of DEGs in ZZZ3 KD ESCs vs. SCR control. Blue dots represent down-regulated genes (275) while yellow dots represent upregulated genes (259). The axes represent the logFC and the adjusted *p* value related to each gene.

(D) Heatmap of the Z-scaled logCPM expression values of genes encoding small and large ribosomal proteins.

(E) GO BPs gene sets significantly associated with the ZZZ3 KD condition as resulted from the GSEA performed on the logFC*-log(*p*-value) ranked genes. The normalized enriched score (NES) is reported for each gene set having different color shades according to the -log(*p* value).

target eukaryotic initiation factor 4E (Figure 6H). S6K phosphorylates several targets involved in translation initiation, leading to increased ribosome biogenesis and translation initiation complex assembly. In an effort to restore these dysregulated signaling pathways to their baseline levels observed in SCR control cells, we treated ZZZ3-silenced cells with insulin-like growth factor-1 (IGF-1), known to activate the PI3K/Akt pathway (Liu et al., 2017), upstream of the mTOR signaling pathway. Western blot analysis of ZZZ3 KD hESCs treated with IGF-1 demonstrated a rescue of the PI3K-AKT-mTOR axis (Figures S5A–S5F). These results collectively suggest that the PI3K, ERK, AKT, and mTOR signaling pathways exhibit reduced activity in ESCs with ZZZ3 KD. Importantly, this diminished activation is reversible, as evidenced by its restoration upon stimulation with IGF-1.

DISCUSSION

Human pluripotent stem cells (PSCs), comprising ESCs and induced PSCs, possess the unique capability to self-renew and differentiate into any of the three germ layers (Evans and Kaufman, 1981; Parrotta et al., 2019a; Thomson et al., 1998). These features make them valuable for diverse biomedical applications including enhancing our understanding of early human development (Zhu and Huangfu, 2013), disease modeling (Lucchino et al., 2022; Orban et al., 2015; Scalise et al., 2022), drug discovery (Chen et al., 2018), regenerative medicine (Parrotta et al., 2019b), and personalized medicine (Shi et al., 2017). To harness their potential, understanding the molecular mechanisms governing pluripotency is crucial. While much research has focused on transcriptional regulation (Chambers et al., 2007; Masui et al., 2007; Niwa et al., 2000) and mechanisms governing differentiation and cellular fate (Chen and Hu, 2017), the relationship between proliferation and pluripotency remains a key question. Studies on diapaused mouse blastocysts and mESCs suggest that pluripotency and proliferation can be independent, challenging the notion of their inseparable connection (Bulut-Karslioglu et al., 2016; Carbognin et al., 2023; Fan et al., 2020; Scognamiglio et al., 2016). In this context, the mTOR pathway, associated with high translation output, emerges as a significant player. Notably, reduced protein synthesis is observed in

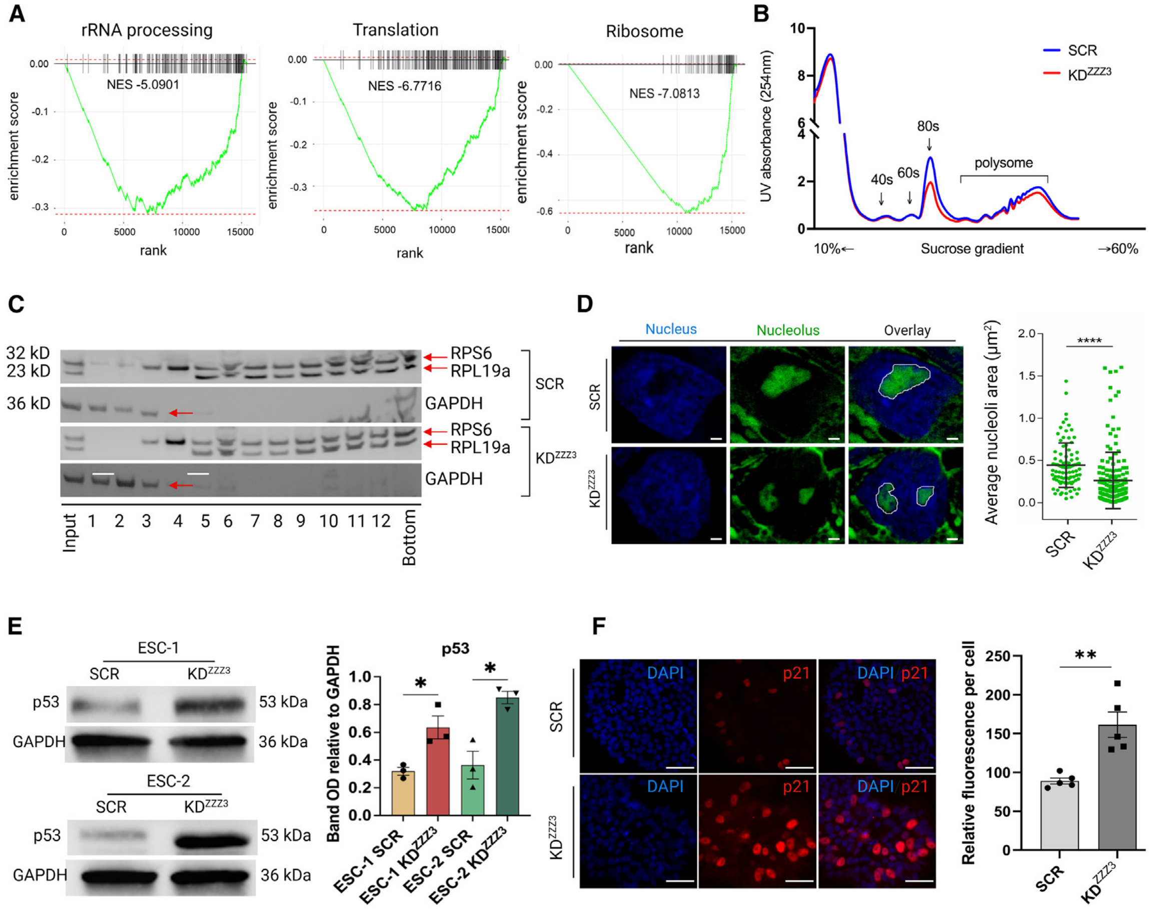

Figure 5. ZZZ3 KD impairs ribosome biogenesis and translation, triggering p53 activation

(A) GSEA enrichment plots show the relative enrichment of gene sets related to rRNA processing (NES = −5.0901), translation (NES = −6.7716), and ribosome (NES = −7.0813) in ZZZ3 KD. The adjusted p value (padj) for both gene sets is equal to 0.019.

(B) Polysome profiling absorbance, measured at 254 nm, was conducted on ZZZ3 KD cells and SCR control cell extracts fractionated on a 10%–60% sucrose gradient. Peaks corresponding to free 40S and 60S subunits, 80S monosomes, and polysomes are indicated on the gradient.

(C) Immunoblot analysis on equal volumes of whole-cell polysome fractions. RPS6 and RPL19a were used as references for small and large ribosomal subunits, respectively. GAPDH served as a marker for proteins not associated with ribosomes. The numbers below the image refer to different fractions as follows: free ribosome (1–3), 40S subunit (4), 60S subunit (5), 80S monosomes (6), and polysomes (7–12). Full-length blots are provided in File S1.

(D) Representative image for nucleolar staining (green) showing a reduced size of nucleoli in ZZZ3 KD cells compared to control. Nuclear DNA was counterstained with DAPI (blue). Scale bar, 1 μm. The measurement of the area of all nucleoli counted within a total of 80 nuclei from 3 independent experiments using ImageJ software. Scatterplots on the right indicate the value of individual cellular measurements, with the horizontal line indicating the mean. Statistical significance was computed as unpaired two-tailed t test of means, ****$p < 0.0001$.

(E) Quantitative immunoblot analysis of p53 demonstrates increased expression in ZZZ3 KD hESCs. GAPDH was utilized as a loading control. Full-length blots are available in File S1.

(F) Representative immunofluorescence staining for p21 reveals increased expression in ZZZ3 KD ESCs. DNA was counterstained with DAPI (blue). Scale bar, 50 μm. Quantification of p21 (200 nuclei analyzed) immunofluorescence signals was performed using ImageJ software. Data are presented as mean ± SEM and significance was calculated using t test, *$p \leq 0.05$, **$p \leq 0.01$.

diapause blastocysts, indicating a link between translation regulation and stem cell dormancy (Fu et al., 2014; Scognamiglio et al., 2016; Zoncu et al., 2011). Recent investigations reported that inhibition of mTOR can delay the progression of human blastoids and PSCs (Iyer et al., 2023). Here, we focused on ZZZ3, a protein shown to be implicated in regulating genes encoding ribosomal proteins in mESCs and human adenocarcinoma cells (Fischer et al., 2021; Mi et al., 2018). To elucidate the specific role of ZZZ3 in hESCs, we employed a targeted approach by downregulating its

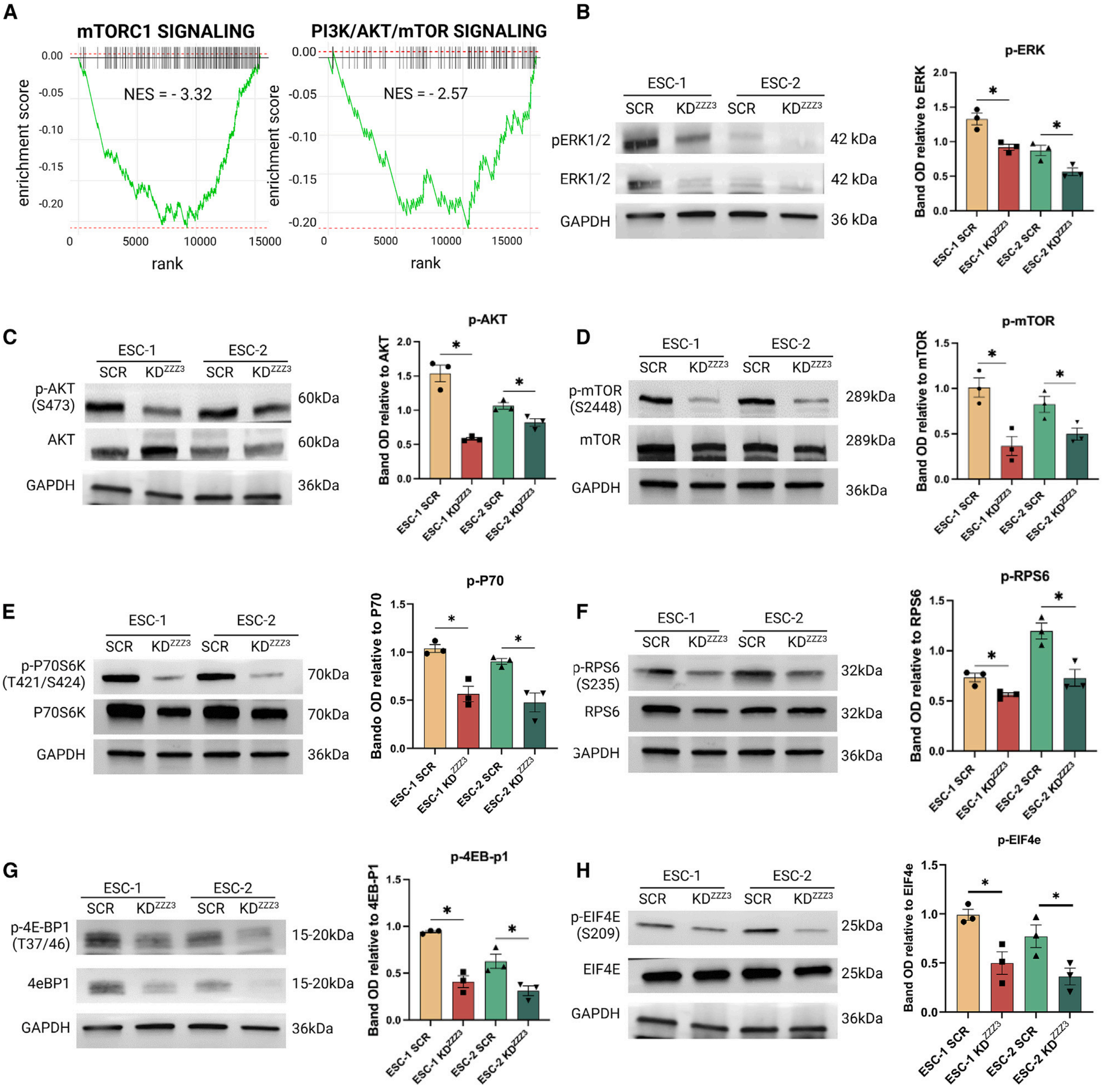

Figure 6. KD of ZZZ3 reduces the PI3K-AKT-mTOR axis

(A) GSEA plots show the relative enrichment of gene sets associated with mTORC1 (NES = −3.32) and PI3K/AKT/mTOR (NES = −2.57) pathways in ZZZ3 KD hESCs. The adjusted *p* value (padj) for both gene sets is equal to 0.0057.

(B–H) Quantitative immunoblot analysis was performed for p-ERK 1/2 (Thr202/Tyr204) and total ERK 1/2 (B); pAKT (Ser473) and total AKT (C); pmTOR (Ser2448) and total mTOR (D); pP70S6 Kinase (Thr421/Ser424) and total P70S6 kinase (E); pRPS6 (Ser235) and total RPS6 (F); p4E-BP1 (Thr37/46) and total 4E-BP1 (G); peIF4E (Ser209) and total eIF4E (H). GAPDH was used as loading control. Full-length blots are available in File S1. Quantification of protein expression was performed using OD measurement. Data are presented as mean ± SEM from $n = 3$ independent experiments. Significance was calculated vs. relative SCR ESCs using t test, $*p \leq 0.05$.

expression. Subsequently, we conducted a comparative analysis, contrasting the features of cells exhibiting diminished ZZZ3 expression with those maintaining normal levels of ZZZ3. ZZZ3 KD in hESCs did not affect pluripotency markers or differentiation capabilities. However, a substantial decrease in proliferation, as evidenced by reduced Ki67 and cell cycle analysis, was observed. The reduction in proliferative capacity was associated with downregulation in the ERK/PI3K/AKT/mTOR signaling pathway, crucial for cell cycle progression and growth. Further analysis revealed that ZZZ3 depletion led to impaired ribosome biogenesis, nucleolar shape remodeling, and p53 activation. The observed elevation in p53 levels and its downstream target, p21, in ZZZ3-depleted cells hints at a potential protective mechanism against impaired ribosome biogenesis. This phenomenon could serve as a promising avenue for future investigation. In summary, this research uncovers three main findings: first, proliferation can be uncoupled from pluripotency in hESCs *in vitro*; second, ZZZ3 is crucial for maintaining a high proliferative state by ensuring proper ribosome biogenesis and translation through modulation of the PI3K/AKT/mTOR pathway; and third, ZZZ3 depletion leads to impaired ribosome biogenesis and p53 activation, preventing the propagation of cells with compromised protein synthesis.

EXPERIMENTAL PROCEDURES

Resource availability

Lead contact

Further information and requests for resources and reagents should be directed to and will be fulfilled by the lead contact, Giovanni Cuda (cuda@unicz.it).

Materials availability

Reagents generated in this study are available form the lead contact without restriction.

Data and code availability

Raw data generated by next-generation sequencing platforms, with per-base quality scores (e.g., BAM, SFF, and HDF5 files or paired FASTQ/QUAL files), were deposited in the Gene Expression Omnibus (GEO) dataset under the accession number GEO: GSE242489. LC-MS/MS proteomic data were completely submitted to the PRIDE ProteomeXchange database under the accession number PRIDE: PXD047165.

Generation of ZZZ3 KD ESCs

hESCs with stable KD of ZZZ3 gene were generated by transfecting cells with PB transposon plasmids system with PB transposase expression vector pBase. Three PB U6-promoter-driven shRNAs against ZZZ3 mRNA or scramble control were purchased from Vector Builder. For DNA transfection, hESCs were dissociated as single cells using TrypLE Select Enzyme (1X) (Thermo Fisher Scientific), and 250,000 cells were co-transfected with PB constructs (550 ng) and pBase plasmid (550 ng) using FuGENE HD transfection reagent (3.9 μL) (Promega), following the manufacturer's instruction. Cells were cultured onto Matrigel-coated 12-well plate in mTeSR1 Plus medium (STEMCELL Technologies, Vancouver, Canada) with 10 μM Y27632 (ROCKi, Rho-associated kinase inhibitor, Selleckchem). After 48 h, transfected cells were selected with hygromycin B (200 μg/mL, Thermo Fisher Scientific) diluted in mTeSR1 Plus for 2 weeks before performing experiments. The sequences of PB ZZZ3 shRNA are provided in the Table S3.

Plasmids for CRISPRi and cloning strategy

The CRISPRi system, comprising the pPB-Ins-TRE3Gp-KRAB-dCas9-ecDHFR-IRES-GFP-EF1Ap-Puro (Addgene #183410; https://www.addgene.org/183410/) and pPB-Ins-U6p-sgRNAentry-EF1Ap-TetOn3G-IRES-Neo plasmids (Addgene #183411; https://www.addgene.org/183411/), employs a DOX-inducible approach with KRAB domain fused to a catalytically inactive Cas9 (KRAB-dCas9), alongside a DHFR protein degron, stabilized by trimethoprim (TMP) treatment. An IRES-EGFP fragment downstream of the KRAB-dCas9 allows for tracking of CRISPRi induction. The PB gRNA delivery and transactivation plasmid contains a sgRNA driven by a U6 promoter and a TET-on 3G-IRES-Neomycin resistance cassette under the control of a constitutive EF1α promoter (Tang et al., 2022). Three sgRNAs targeting the ZZZ3 coding sequence promoter, along with a non-targeting sgRNA control, were designed using the CRISPR Design Tool (http://crispr.mit.edu/) and synthetized by the Eurofins genomics (https://eurofinsgenomics.eu/). The sgRNA sequences are available in Table S3. The cloning process started with the digestion of 10μg of the sgRNA delivery and transactivation plasmid using the Esp3I restriction enzyme. After digestion, the plasmid underwent dephosphorylation to remove phosphate groups, followed by purification. Each pair of sgRNA oligos was phosphorylated, annealed, and ligated into the digested plasmid using DNA ligase. The ligated DNA was transformed into DH5α bacteria cells, and individual colonies were selected. Plasmid DNA was isolated from each colony and sequenced to confirm the presence of desired sequences and the integrity of the construct before using it for ESCs Neon transfection.

Neon transfection

hESCs were dissociated into single cells using Accutase and resuspension in Neon Resuspension Buffer R. Each electroporation involved combining cells with 2 μg of plasmid DNA and pulsing twice with specific parameters (voltage of 1,100 and a width of 30 ms). Post-electroporation, cells were plated onto Matrigel-coated plates with mTeSR1 medium. Selection with puromycin (0.5 μg/mL), hygromycin (200 μg/mL), or neomycin (200 μg/mL) was applied 48 h after electroporation.

Generation of CRISPRi ESCs

ESCs were initially co-transfected with the hyperactive PB transposase (helper PBase) plasmid and the PB-based CRISPRi to generate stable ESC lines carrying integrated CRISPRi via puromycin selection. Subsequently, CRISPRi ESCs were co-transfected with the hyperactive PB transposase plasmid and sgRNA delivery and transactivation. Stable ESC lines carrying integrated CRISPRi and sgRNA transgenes were established through puromycin

(0.5 μg/mL) and neomycin (200 μg/mL) selection. CRISPRi induction was achieved with Dox addition and monitored by EGFP fluorescence. ZZZ3 interference efficiency was evaluated using immunoblot analysis (Figure S2D, with TET-inducible ZZZ3 silencing sequences provided in Table S3. CRISPRi induction and interference efficiency at 72 and 96 h were comparable in presence or absence of TMP, thus TMP was withdrawn.

Transfection of ESCs with PB inducible ZZZ3 expression vector (Tet-On)

The ESCs were co-transfected with the hyperactive PB transposase (helper PBase) plasmid and the following PiggyBac Inducible ZZZ3 Expression Vectors (Tet-On) purchased from Vector Builder (https://en.vectorbuilder.com/): VB231123-1578zqe (Scramble); VB231126-1293tdh (ZZZ3 shRNA#1); VB231123-1572zpv (ZZZ3 shRNA#2). These vectors contain the miR30 sequence facilitating the formation of mature shRNA for knockdown. Transfection was performed using the Neon electroporation system using the procedure and plasmid DNA concentration described earlier. Stable ESC lines carrying the shRNA transgenes were established through hygromycin selection (200 μg/mL). Silencing of ZZZ3 was achieved in the presence of doxycycline for 96 h.

EBs formation assay

EBs differentiation was performed as previously described in the study by Scaramuzzino et al. (2021). Bright-field images of EBs were captured using an imaging system (DMi8, Leica Microsystems). SCR and ZZZ3 KD ESCs were further differentiated into ectoderm, mesoderm, and endoderm using media supplements provided by a commercial kit (R&D Systems) according to the manufacturer's instructions. Differentiated cells were stained with anti-human SOX17 antibody for endoderm, anti-human BRACHYURY antibody for mesoderm, and anti-human OTX2 antibody for ectoderm. Subsequently, cells were stained with 557-conjugated Donkey anti-IgG secondary antibody (red), and the nuclei were counterstained with DAPI.

RNA-seq library preparation and analysis

Total RNA was extracted using TRIzol reagents (Invitrogen, 15596026) according to the manufacturer's protocol. For library preparation, the quantity and quality of the starting RNA were checked by Qubit and Bioanalyzer (Agilent). 1 μg of total RNA was subjected to poly(A) enrichment and library preparation using the TruSeq Stranded mRNA Library Prep Kit (Illumina) following the manufacturer's instructions. Libraries were sequenced on Illumina NextSeq 1000 System (paired-end 60 + 60 bp reads). After quality controls with FastQC (https://www.bioinformatics.babraham.ac.uk/projects/fastqc/), raw reads were aligned to the human reference genome (hg38/GRCh38) using STAR 2.7.1a (Dobin et al., 2013) (with parameters–outFilterMismatchNmax 999 –outFilterMismatchNoverLmax 0.04). Gene expression levels were quantified with featureCounts v1.6.3 (Liao et al., 2014) (options: -t exon -g gene_name) using GENCODE 32 annotation. ZZZ3 is annotated in GENCODE as AC118549.1. Multi-mapped reads were excluded from quantification. Gene expression counts were next analyzed using the edge*R* package (Robinson et al., 2010). After low expressed genes filtration (1 count per million (CPM) in less than 3 samples), normalization factors were calculated using the trimmed-mean of M-values method implemented in the calcNormFactors function, and CPM was obtained using normalized library sizes. Differential expression analysis was carried out by considering a paired design thus fitting a GLM with the formula "$\sim 0 + \text{condition} + \text{cell\_type}$". A QLF test was performed to compare the "ZZZ3 KD - SCR" groups. Genes were marked as significantly differentially expressed when having | logFC | > 1 and adjusted p value <0.05 (Benjamini-Hochberg p value correction). Principal component analysis of the expression dataset was performed using the Prcomp function implemented in the R stats package. Gene expression heatmaps with hierarchical clustering based on euclidean distance were generated using the ComplexHeatmap R package scaling logCPM values as Z scores across samples (Gu et al., 2016). GSEA was conducted by using GSEA software (Subramanian et al., 2005) on logFC*-log(p value) ranked genes.

IP

Subconfluent cells were harvested and cross-linked using 0.05 mM DSP (Thermo Fisher Scientific) for 2 h on ice. The reaction was quenched using 20 mM Tris-HCl, pH 7.4, for 15 min on ice, followed by two washes with PBS. Cells were centrifuged at 5,000 rpm for 5 min at 4°C and resuspended in IP buffer (50 mM sodium phosphate pH 7.2, 250 mM NaCl, 0.1% Triton X-100, 0.1 nM $ZnCl_2$) supplemented with protease and phosphatase inhibitor cocktails (Thermo Fisher Scientific). The cell suspension was then sonicated with a Diagenode Bioruptor (Settings: 10 s ON, 10 s OFF, high power) and centrifuged at 13,000 rpm for 20 min at 4°C. Protein concentration was determined using the Bradford assay. 1 mg of cell lysate was immunoprecipitated overnight at 4°C under rotation with Dynabeads Protein A (1.5 mg) (Thermo Fisher Scientific) and 10 μg anti-ZZZ3 antibody (Abcam) or 10 μg anti-IgG antibody (Diagenode). The next day, the immunoprecipitated complexes were washed three times with an IP buffer. Precipitated proteins were either trypsin-digested for mass spectrometry analysis or denatured in a Laemmli sample buffer at 95°C for 5 min and analyzed by western Blot. For ZZZ3 interactome by nanoLC-MS/MS, IP using anti-ZZZ3 antibody was performed in biological quadruplicate.

Mass spectrometry

ZZZ3 and its interactors were released from the magnetic beads by pre-digestion in 100 μL of digestion buffer (100 mM Tris-HCl pH 8.5, 0.1% Triton X-100, 400 ng trypsin Proteomics Grade [Sigma-Aldrich]) for 15 min at 37°C with shaking (500 rpm). After on-bead pre-digestion by trypsin, the supernatant was collected and subjected to tryptic digestion. Proteins were reduced by 10 mM dithiothreitol (DTT) for 1 h at 37°C under shaking (500 rpm). Cysteines were alkylated by treatment with 24 mM iodoacetamide for 1 h at 37°C with agitation (500 rpm) in the dark. The alkylation reaction was quenched by adding 2 mM DTT (final concentration) for 30 min at 37°C. Finally, proteins were incubated with 200 ng trypsin overnight at 37°C with agitation to complete digestion (Bernaudo et al., 2015). The resulting tryptic peptides (20 μL corresponding to approximately 20 μg of peptides) were purified by strong cation exchange StageTips method (Rappsilber et al., 2007) to remove eventual detergent residues. Before loading on the StageTips, the digested samples were acidified by adding 80%

acetonitrile-0.5% formic acid (wash solution 2). The StageTip was conditioned by adding 20% acetonitrile-0.5% formic acid (wash solution 1, 50 μL) and 50 μL of W2. Peptides were loaded by slowly letting the fluid pass through one plug using a benchtop centrifuge. After 2 washes with 50 μL of Solution W2 and 50 μL of Solution W1, respectively, peptides were eluted by adding 7 μL of 500 mM ammonium acetate-20% acetonitrile, diluted with 33 μL of formic acid 0.1% and analyzed by nanoLC-MS/MS.

LC-MS/MS analysis

Peptides were separated by an Easy nLC-1000 chromatographic instrument coupled to a Q-Exactive "classic" mass spectrometer (both from Thermo Fisher Scientific). All the liquid chromatography-tandem mass spectrometry (LC-MS/MS) analyses were carried using a linear gradient of 75 min at a flow rate of 230 nL/min on a 15 cm, 75 μm i.d., in-house-made column packed with 3 μm C18 silica particles (Dr. Maisch). The binary gradient was performed using mobile phase A (0.1% FA, 2% ACN) and mobile phase B (0.1% FA and 80% ACN). Peptide separation was obtained at a flow rate of 230 nL/min and ramped using from 3% B to 40% B in 60 min, from 40% to 100% in 13 min; the column was cleaned for 5 min with 100% of B. LC-MS/MS analysis were performed in data-dependent acquisition (DDA) using a top-12 method. Full-scan m/z range was 350–1800, followed by MS/MS scans on the 12 most intense precursor ions. DDA analysis was performed with resolution for full MS scan of 70,000 and of 35,000 for MS/MS scan; the isolation window was 1.6 m/z. AGC target for full MS was 1e6 and 1e5 for MS/MS scan. The maximum injection time was set to 50 ms for full MS scans and 120 ms for MS/MS scans. Fragmentation was performed by higher-energy collisional dissociation (HCD) using 25% normalized collision energy and a dynamic exclusion of 20s. The mass spectrometry proteomics data have been deposited to the ProteomeXchange Consortium via the PRIDE (Perez-Riverol et al., 2022) partner repository.

Statistical analysis

Experimental data are presented as means ± standard deviation of the mean unless stated otherwise. Statistical significance was calculated unless stated otherwise by two-tailed unpaired t test on two experimental conditions with $p \leq 0.05$ considered statistically significant. Statistical significance levels are denoted as follows: *$p \leq 0.05$; **$p \leq 0.01$; ***$p \leq 0.001$; ****$p \leq 0.0001$. No statistical methods were used to predetermine sample size. Super exact test was performed to test the significance of the Venn diagram by the *R* package Exact.

SUPPLEMENTAL INFORMATION

Supplemental information can be found online at https://doi.org/10.1016/j.stemcr.2024.04.002.

ACKNOWLEDGMENTS

Images were created using the BioRender software (https://app.biorender.com/).

This work was supported by #NextGenerationEU (NGEU) and funded by the Ministry of University and Research (MUR), National Recovery and Resilience Plan (NRRP), project MNESYS (PE0000006) – A multiscale integrated approach to the study of the nervous system in health and disease (DN. 1553 11.10.2022).

AUTHOR CONTRIBUTIONS

M.L.C., V.L., E.I.P., and G.C. conceived and designed the experiments; M.L.C., V.L., and E.I.P. performed most of the experiments; S.S., C.Z., D.V., L.S., D.S.M., and M.S.M. performed the experiments; C.C. performed RNA-seq bioinformatics analyses; M.L.C., V.L., E.I.P., G.C., and A.P. performed the data analysis and interpretation; E.I.P., V.L., and G.C. drafted the manuscript; G.C. provided financial support; G.M. provided conceptual advice and reagents and helped in proofreading the paper; G.R. provided reagents, protocols, and advices for gRNAs cloning and CRISPRi strategy; and E.I.P. and G.C. supervised the study and wrote the final version of the paper. All authors read and approved the final manuscript.

DECLARATION OF INTERESTS

The authors declare no competing interests.

Received: September 18, 2023
Revised: April 3, 2024
Accepted: April 4, 2024
Published: May 2, 2024

REFERENCES

Bernaudo, F., Monteleone, F., Mesuraca, M., Krishnan, S., Chiarella, E., Scicchitano, S., Cuda, G., Morrone, G., Bond, H.M., and Gaspari, M. (2015). Validation of a Novel Shotgun Proteomic Workflow for the Discovery of Protein–Protein Interactions: Focus on ZNF521. J. Proteome Res. *14*, 1888–1899.

Bohlen, J., Roiuk, M., and Teleman, A.A. (2021). Phosphorylation of ribosomal protein S6 differentially affects mRNA translation based on ORF length. Nucleic Acids Res. *49*, 13062–13074.

Böhm, R., Imseng, S., Jakob, R.P., Hall, M.N., Maier, T., and Hiller, S. (2021). The dynamic mechanism of 4E-BP1 recognition and phosphorylation by mTORC1. Mol. Cell *81*, 2403–2416.e5.

Bullwinkel, J., Baron-Lühr, B., Lüdemann, A., Wohlenberg, C., Gerdes, J., and Scholzen, T. (2006). Ki-67 protein is associated with ribosomal RNA transcription in quiescent and proliferating cells. J. Cell. Physiol. *206*, 624–635.

Bulut-Karslioglu, A., Biechele, S., Jin, H., Macrae, T.A., Hejna, M., Gertsenstein, M., Song, J.S., and Ramalho-Santos, M. (2016). Inhibition of mTOR induces a paused pluripotent state. Nature *540*, 119–123.

Carbognin, E., Carlini, V., Panariello, F., Chieregato, M., Guerzoni, E., Benvegnù, D., Perrera, V., Malucelli, C., Cesana, M., Grimaldi, A., et al. (2023). Esrrb guides naive pluripotent cells through the formative transcriptional programme. Nat. Cell Biol. *25*, 643–657.

Chambers, I., Silva, J., Colby, D., Nichols, J., Nijmeijer, B., Robertson, M., Vrana, J., Jones, K., Grotewold, L., and Smith, A. (2007). Nanog safeguards pluripotency and mediates germline development. Nature *450*, 1230–1234.

Chen, K.G., Mallon, B.S., Park, K., Robey, P.G., McKay, R.D.G., Gottesman, M.M., and Zheng, W. (2018). Pluripotent Stem Cell Platforms for Drug Discovery. Trends Mol. Med. *24*, 805–820.

Chen, Q., and Hu, G. (2017). Post-transcriptional regulation of the pluripotent state. Curr. Opin. Genet. Dev. *46*, 15–23.

Dobin, A., Davis, C.A., Schlesinger, F., Drenkow, J., Zaleski, C., Jha, S., Batut, P., Chaisson, M., and Gingeras, T.R. (2013). STAR: ultrafast universal RNA-seq aligner. Bioinformatics *29*, 15–21.

Evans, M.J., and Kaufman, M.H. (1981). Establishment in culture of pluripotential cells from mouse embryos. Nature *292*, 154–156.

Fan, R., Kim, Y.S., Wu, J., Chen, R., Zeuschner, D., Mildner, K., Adachi, K., Wu, G., Galatidou, S., Li, J., et al. (2020). Wnt/Beta-catenin/Esrrb signalling controls the tissue-scale reorganization and maintenance of the pluripotent lineage during murine embryonic diapause. Nat. Commun. *11*, 5499.

Fischer, V., Plassard, D., Ye, T., Reina-San-Martin, B., Stierle, M., Tora, L., and Devys, D. (2021). The related coactivator complexes SAGA and ATAC control embryonic stem cell self-renewal through acetyltransferase-independent mechanisms. Cell Rep. *36*, 109598.

Fu, Z., Wang, B., Wang, S., Wu, W., Wang, Q., Chen, Y., Kong, S., Lu, J., Tang, Z., Ran, H., et al. (2014). Integral Proteomic Analysis of Blastocysts Reveals Key Molecular Machinery Governing Embryonic Diapause and Reactivation for Implantation in Mice1. Biol. Reprod. *90*, 52-1. https://doi.org/10.1095/biolreprod.113.115337.

Gabut, M., Bourdelais, F., and Durand, S. (2020). Ribosome and Translational Control in Stem Cells. Cells *9*, 497.

Gilkes, D.M., Chen, L., and Chen, J. (2006). MDMX regulation of p53 response to ribosomal stress. EMBO J. *25*, 5614–5625.

Gu, Z., Eils, R., and Schlesner, M. (2016). Complex heatmaps reveal patterns and correlations in multidimensional genomic data. Bioinformatics *32*, 2847–2849.

Holz, M.K., Ballif, B.A., Gygi, S.P., and Blenis, J. (2005). mTOR and S6K1 Mediate Assembly of the Translation Preinitiation Complex through Dynamic Protein Interchange and Ordered Phosphorylation Events. Cell *123*, 569–580.

Hwang, Y.S., Chung, B.G., Ortmann, D., Hattori, N., Moeller, H.C., and Khademhosseini, A. (2009). Microwell-mediated control of embryoid body size regulates embryonic stem cell fate via differential expression of WNT5a and WNT11. Proc. Natl. Acad. Sci. USA *106*, 16978–16983.

Iyer, D.P., Weijden, V.A.V.D., Khoei, H.H., McCarthy, A., Rayon, T., Simon, C.S., Dunkel, I., Wamaitha, S.E., Elder, K., Snell, P., et al. (2023). Delay of human early development via in vitro diapause. Preprint at bioRxiv. https://doi.org/10.1101/2023.05.29.541316.

Jastrzebski, K., Hannan, K.M., Tchoubrieva, E.B., Hannan, R.D., and Pearson, R.B. (2007). Coordinate regulation of ribosome biogenesis and function by the ribosomal protein S6 kinase, a key mediator of mTOR function. Growth Factors *25*, 209–226.

Jiao, L., Liu, Y., Yu, X.Y., Pan, X., Zhang, Y., Tu, J., Song, Y.H., and Li, Y. (2023). Ribosome biogenesis in disease: new players and therapeutic targets. Signal Transduct. Targeted Ther. *8*, 15.

Li, M., and Izpisua Belmonte, J.C. (2018). Deconstructing the pluripotency gene regulatory network. Nat. Cell Biol. *20*, 382–392.

Liao, Y., Smyth, G.K., and Shi, W. (2014). featureCounts: an efficient general purpose program for assigning sequence reads to genomic features. Bioinformatics *30*, 923–930.

Liu, Q., Guan, J.Z., Sun, Y., Le, Z., Zhang, P., Yu, D., and Liu, Y. (2017). Insulin-like growth factor 1 receptor-mediated cell survival in hypoxia depends on the promotion of autophagy via suppression of the PI3K/Akt/mTOR signaling pathway. Mol. Med. Rep. *15*, 2136–2142.

Lucchino, V., Scaramuzzino, L., Scalise, S., Lo Conte, M., Zannino, C., Benedetto, G.L., Aguglia, U., Ferlazzo, E., Cuda, G., and Parrotta, E.I. (2022). Insights into the Genetic Profile of Two Siblings Affected by Unverricht-Lundborg Disease Using Patient-Derived hiPSCs. Cells *11*, 3491.

Martin, G.R. (1981). Isolation of a pluripotent cell line from early mouse embryos cultured in medium conditioned by teratocarcinoma stem cells. Proc. Natl. Acad. Sci. USA *78*, 7634–7638.

Masui, S., Nakatake, Y., Toyooka, Y., Shimosato, D., Yagi, R., Takahashi, K., Okochi, H., Okuda, A., Matoba, R., Sharov, A.A., et al. (2007). Pluripotency governed by Sox2 via regulation of Oct3/4 expression in mouse embryonic stem cells. Nat. Cell Biol. *9*, 625–635.

Mayer, C., and Grummt, I. (2006). Ribosome biogenesis and cell growth: mTOR coordinates transcription by all three classes of nuclear RNA polymerases. Oncogene *25*, 6384–6391.

Mi, W., Zhang, Y., Lyu, J., Wang, X., Tong, Q., Peng, D., Xue, Y., Tencer, A.H., Wen, H., Li, W., et al. (2018). The ZZ-type zinc finger of ZZZ3 modulates the ATAC complex-mediated histone acetylation and gene activation. Nat. Commun. *9*, 3759.

Morrison, D.K. (2012). MAP Kinase Pathways. Cold Spring Harbor Perspect. Biol. *4*, a011254.

Ng, H.H., and Surani, M.A. (2011). The transcriptional and signalling networks of pluripotency. Nat. Cell Biol. *13*, 490–496.

Niwa, H., Miyazaki, J., and Smith, A.G. (2000). Quantitative expression of Oct-3/4 defines differentiation, dedifferentiation or self-renewal of ES cells. Nat. Genet. *24*, 372–376.

Orban, M., Goedel, A., Haas, J., Sandrock-Lang, K., Gärtner, F., Jung, C.B., Zieger, B., Parrotta, E., Kurnik, K., Sinnecker, D., et al. (2015). Functional Comparison of Induced Pluripotent Stem Cell- and Blood-Derived GPIIbIIIa Deficient Platelets. L. Cheng, ed. *10*, e0115978.

Parrotta, E.I., Scalise, S., Taverna, D., De Angelis, M.T., Sarro, G., Gaspari, M., Santamaria, G., and Cuda, G. (2019a). Comprehensive proteogenomic analysis of human embryonic and induced pluripotent stem cells. J. Cell Mol. Med. *23*, 5440–5453.

Parrotta, E.I., Scalise, S., Scaramuzzino, L., and Cuda, G. (2019b). Stem Cells: The Game Changers of Human Cardiac Disease Modelling and Regenerative Medicine. Int. J. Mol. Sci. *20*, 5760.

Perez-Riverol, Y., Bai, J., Bandla, C., García-Seisdedos, D., Hewapathirana, S., Kamatchinathan, S., Kundu, D.J., Prakash, A., Frericks-Zipper, A., Eisenacher, M., et al. (2022). The PRIDE database resources in 2022: a hub for mass spectrometry-based proteomics evidences. Nucleic Acids Res. *50*, D543–D552.

Rappsilber, J., Mann, M., and Ishihama, Y. (2007). Protocol for micro-purification, enrichment, pre-fractionation and storage of peptides for proteomics using StageTips. Nat. Protoc. *2*, 1896–1906.

Robinson, M.D., McCarthy, D.J., and Smyth, G.K. (2010). edgeR : a Bioconductor package for differential expression analysis of digital gene expression data. Bioinformatics *26*, 139–140.

Rodriguez-Corona, U., Sobol, M., Rodriguez-Zapata, L.C., Hozak, P., and Castano, E. (2015). Fibrillarin from Archaea to human: Review on fibrillarin. Biol. Cell. *107*, 159–174.

Roux, P.P., Shahbazian, D., Vu, H., Holz, M.K., Cohen, M.S., Taunton, J., Sonenberg, N., and Blenis, J. (2007). RAS/ERK Signaling Promotes Site-specific Ribosomal Protein S6 Phosphorylation via RSK and Stimulates Cap-dependent Translation. J. Biol. Chem. *282*, 14056–14064.

Scalise, S., Zannino, C., Lucchino, V., Lo Conte, M., Scaramuzzino, L., Cifelli, P., D'Andrea, T., Martinello, K., Fucile, S., Palma, E., et al. (2022). Human iPSC Modeling of Genetic Febrile Seizure Reveals Aberrant Molecular and Physiological Features Underlying an Impaired Neuronal Activity. Biomedicines *10*, 1075.

Scaramuzzino, L., Lucchino, V., Scalise, S., Lo Conte, M., Zannino, C., Sacco, A., Biamonte, F., Parrotta, E.I., Costanzo, F.S., and Cuda, G. (2021). Uncovering the Metabolic and Stress Responses of Human Embryonic Stem Cells to FTH1 Gene Silencing. Cells *10*, 2431.

Scognamiglio, R., Cabezas-Wallscheid, N., Thier, M.C., Altamura, S., Reyes, A., Prendergast, Á.M., Baumgärtner, D., Carnevalli, L.S., Atzberger, A., Haas, S., et al. (2016). Myc Depletion Induces a Pluripotent Dormant State Mimicking Diapause. Cell *164*, 668–680.

Sheridan, S.D., Surampudi, V., and Rao, R.R. (2012). Analysis of Embryoid Bodies Derived from Human Induced Pluripotent Stem Cells as a Means to Assess Pluripotency. Stem Cell. Int. *2012*, 738910.

Shi, Y., Inoue, H., Wu, J.C., and Yamanaka, S. (2017). Induced pluripotent stem cell technology: a decade of progress. Nat. Rev. Drug Discov. *16*, 115–130.

Subramanian, A., Tamayo, P., Mootha, V.K., Mukherjee, S., Ebert, B.L., Gillette, M.A., Paulovich, A., Pomeroy, S.L., Golub, T.R., Lander, E.S., and Mesirov, J.P. (2005). Gene set enrichment analysis: A knowledge-based approach for interpreting genome-wide expression profiles. Proc. Natl. Acad. Sci. USA *102*, 15545–15550.

Sun, X., Bizhanova, A., Matheson, T.D., Yu, J., Zhu, L.J., and Kaufman, P.D. (2017). Ki-67 Contributes to Normal Cell Cycle Progression and Inactive X Heterochromatin in p21 Checkpoint-Proficient Human Cells. Mol. Cell Biol. *37*. e00569005699-16.

Tang, W.W.C., Castillo-Venzor, A., Gruhn, W.H., Kobayashi, T., Penfold, C.A., Morgan, M.D., Sun, D., Irie, N., and Surani, M.A. (2022). Sequential enhancer state remodelling defines human germline competence and specification. Nat. Cell Biol. *24*, 448–460.

Tee, W.W., and Reinberg, D. (2014). Chromatin features and the epigenetic regulation of pluripotency states in ESCs. Development *141*, 2376–2390.

Thomson, J.A., Itskovitz-Eldor, J., Shapiro, S.S., Waknitz, M.A., Swiergiel, J.J., Marshall, V.S., and Jones, J.M. (1998). Embryonic Stem Cell Lines Derived from Human Blastocysts. Science *282*, 1145–1147.

Tollervey, D., Lehtonen, H., Jansen, R., Kern, H., and Hurt, E.C. (1993). Temperature-sensitive mutations demonstrate roles for yeast fibrillarin in pre-rRNA processing, pre-rRNA methylation, and ribosome assembly. Cell *72*, 443–457.

Xu, X., Ahmed, T., Wang, L., Cao, X., Zhang, Z., Wang, M., Lv, Y., Kanwal, S., Tariq, M., Lin, R., et al. (2022). The mTORC1-eIF4F axis controls paused pluripotency. EMBO Rep. *23*, e53081.

Zhu, Z., and Huangfu, D. (2013). Human pluripotent stem cells: an emerging model in developmental biology. Development *140*, 705–717.

Zoncu, R., Efeyan, A., and Sabatini, D.M. (2011). mTOR: from growth signal integration to cancer, diabetes and ageing. Nat. Rev. Mol. Cell Biol. *12*, 21–35.

Stem Cell Reports, Volume *19*

Supplemental Information

Unraveling the impact of ZZZ3 on the mTOR/ribosome pathway in human embryonic stem cells homeostasis

Michela Lo Conte, Valeria Lucchino, Stefania Scalise, Clara Zannino, Desirèe Valente, Giada Rossignoli, Maria Stella Murfuni, Chiara Cicconetti, Luana Scaramuzzino, Danilo Swann Matassa, Anna Procopio, Graziano Martello, Giovanni Cuda, and Elvira Immacolata Parrotta

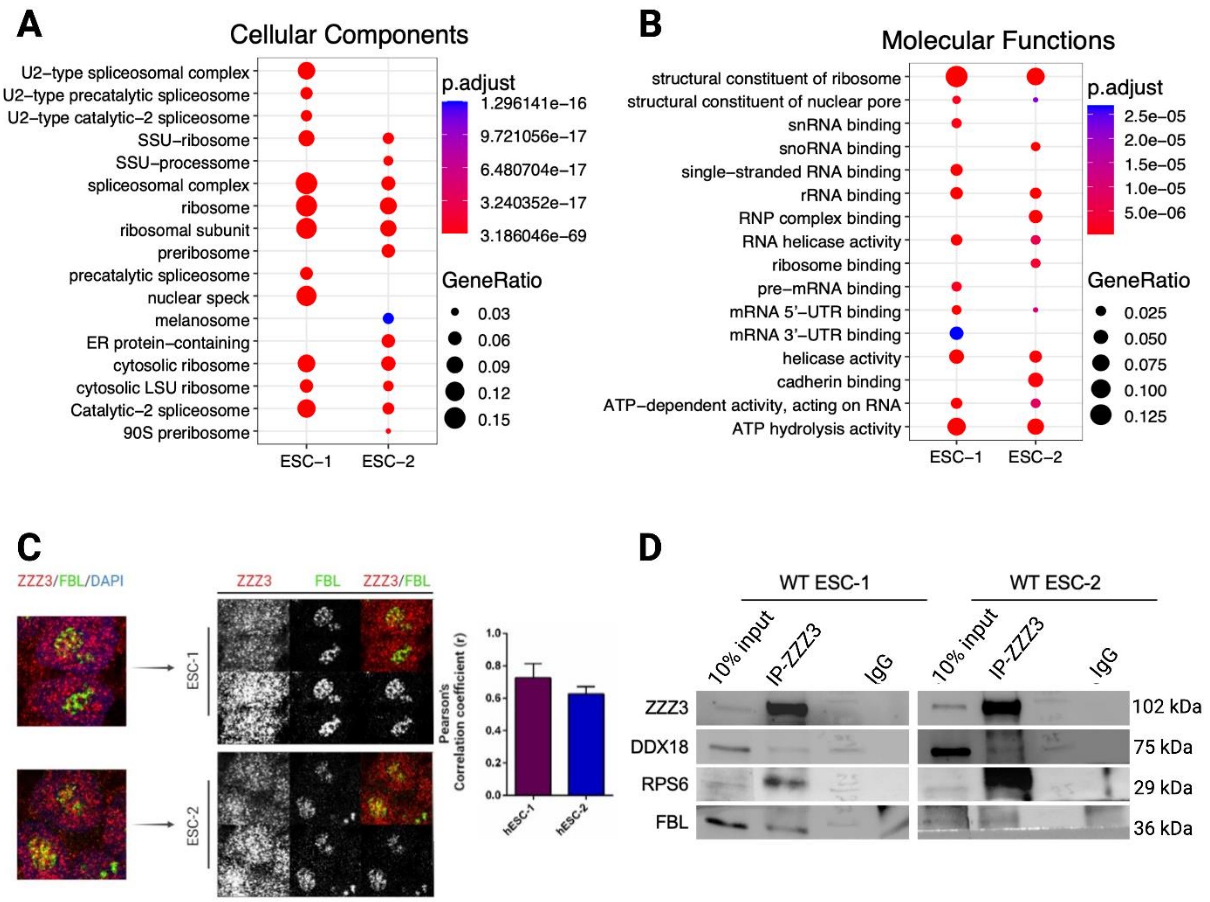
A
Cellular Components
U2–type spliceosomal complex
U2–type precatalytic spliceosome
U2–type catalytic–2 spliceosome
SSU–ribosome
SSU–processome
spliceosomal complex
ribosome
ribosomal subunit
preribosome
precatalytic spliceosome
nuclear speck
melanosome
ER protein–containing
cytosolic ribosome
cytosolic LSU ribosome
Catalytic–2 spliceosome
90S preribosome
ESC–1
ESC–2
p.adjust
1.296141e–16
9.721056e–17
6.480704e–17
3.240352e–17
3.186046e–69
GeneRatio
0.03
0.06
0.09
0.12
0.15
B
Molecular Functions
structural constituent of ribosome
structural constituent of nuclear pore
snRNA binding
snoRNA binding
single–stranded RNA binding
rRNA binding
RNP complex binding
RNA helicase activity
ribosome binding
pre–mRNA binding
mRNA 5'–UTR binding
mRNA 3'–UTR binding
helicase activity
cadherin binding
ATP–dependent activity, acting on RNA
ATP hydrolysis activity
ESC–1
ESC–2
p.adjust
2.5e–05
2.0e–05
1.5e–05
1.0e–05
5.0e–06
GeneRatio
0.025
0.050
0.075
0.100
0.125
C
ZZZ3/FBL/DAPI
ZZZ3
FBL
ZZZ3/FBL
ESC-1
ESC-2
Pearson's Correlation coefficient (r)
1.0
0.8
0.6
0.4
0.2
0.0
hESC-1
hESC-2
D
WT ESC-1
WT ESC-2
10% input
IP-ZZZ3
IgG
10% input
IP-ZZZ3
IgG
ZZZ3
DDX18
RPS6
FBL
102 kDa
75 kDa
29 kDa
36 kDa

Figure S1. ZZZ3 is co-expressed with fibrillarin in the nucleolus and interacts with proteins involved in post-transcriptional processes and ribosome biogenesis. (Related to Fig. 1). A. GO for Cellular Components (**A**) and Molecular Functions (**B**) of differentially expressed genes selected on the basis of the *p*-values (p-value < 0.05 corrected by using Benjamini-Hochberg procedure), and fold-change (FC ≥ 2.5). GO analysis was performed in *R* using the Bioconductor package. **C**. Representative immunofluorescence images showing the colocalization of ZZZ3 (red) and Fibrillarin (FBL, green) were captured in wild-type ESC-1 and -2. Utilizing the JACoP BIOP colocalization plugin within the ImageJ software, the degree of colocalization was assessed by calculating the Pearson's correlation coefficient (r) across approximately 40 regions of interest (ROIs), representing the cell areas positive for FBL signal. These ROIs were randomly selected from three distinct stainings of both ESC lines. The intensity-based correlation analysis revealed positive values of the correlation coefficient, indicating the colocalization of ZZZ3 and FBL within the nucleolus of wild-type ESCs. **D**. Immunoprecipitation (IP) with an anti-ZZZ3 antibody followed by Western blot analysis was employed to validate interactome data. Specifically, interactions of ZZZ3 with DDX18, RPS6, and FBL are shown. Full-length uncropped blots are available in Supplementary File S1.

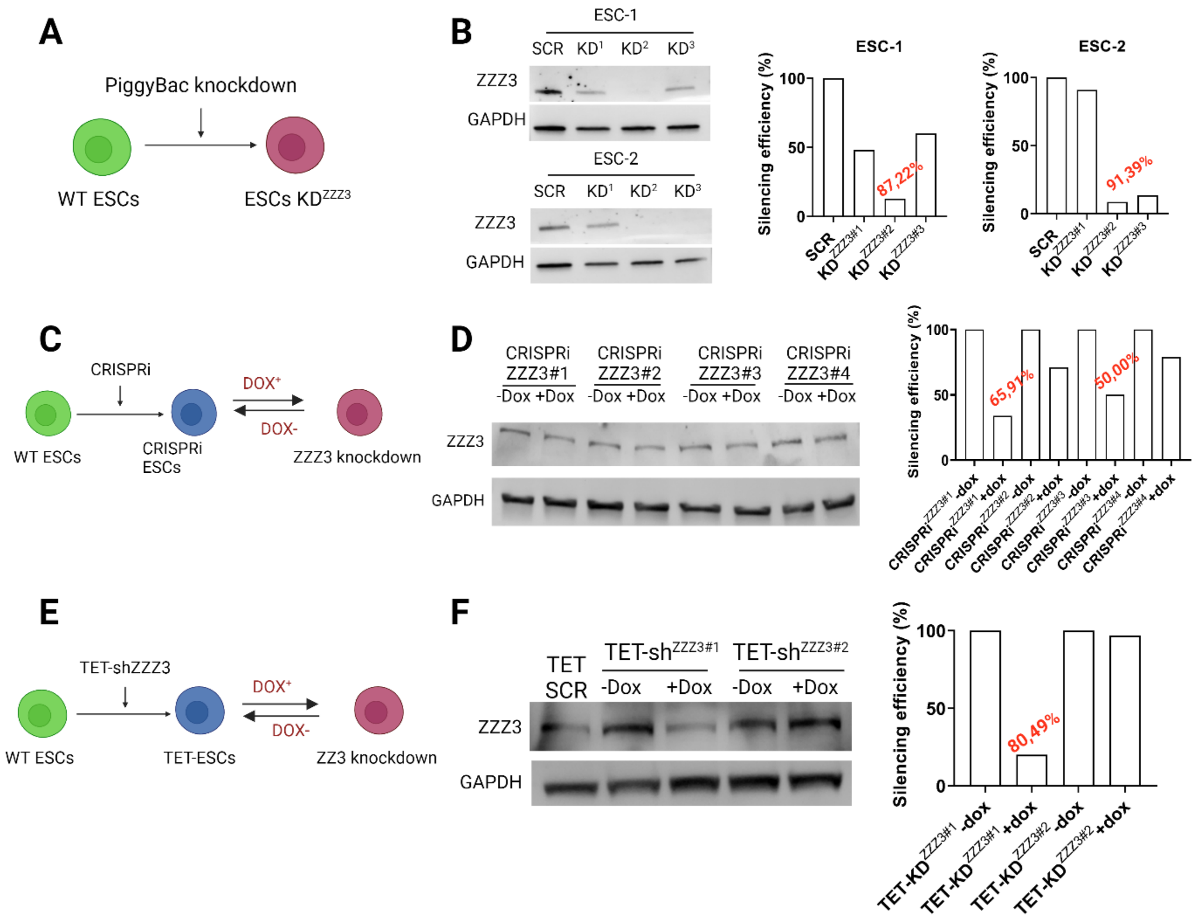
A
PiggyBac knockdown
WT ESCs
ESCs KD ZZZ3
B
ESC-1
SCR KD1 KD2 KD3
ZZZ3
GAPDH
ESC-2
SCR KD1 KD2 KD3
ZZZ3
GAPDH
ESC-1
ESC-2
Silencing efficiency (%)
87,22%
91,39%
C
CRISPRi
DOX+
DOX-
WT ESCs
CRISPRi ESCs
ZZZ3 knockdown
D
CRISPRi ZZZ3#1
CRISPRi ZZZ3#2
CRISPRi ZZZ3#3
CRISPRi ZZZ3#4
-Dox +Dox
65,91%
50,00%
E
TET-shZZZ3
DOX+
DOX-
WT ESCs
TET-ESCs
ZZ3 knockdown
F
TET SCR
TET-sh ZZZ3#1
TET-sh ZZZ3#2
-Dox +Dox
80,49%

Figure S2. Knockdown strategies utilized to silence the ZZZ3 gene in two lines of human ESCs. A. Schematic representation of the PiggyBac vector expressing shRNA against the human ZZZ3 gene under the control of the U6 promoter, utilized to establish stable ZZZ3 knockdown in hESCs. **B**. Immunoblot analysis was performed to evaluate the efficiency of ZZZ3 knockdown using three distinct shRNAs, labelled as KD^1, KD^2, and KD^3. Notable, high efficient ZZZ3 knockdown was observed in cells transfected with shZZZ#2 (KD^2), which were subsequently utilized in this study. **C** and **D**. CRISPR interference (CRISPRi) targeting ZZZ3 mRNA and its validation via relative western blot analysis. CRISPRi-ZZZ3#1 and #3 exhibited effective ZZZ3 knockdown. **E** and **F**. TET-inducible shZZZ3 strategy and its validation by immunoblot analysis. The sequences of shRNA and guide RNA (gRNA) are provided in Supplementary Table S3.

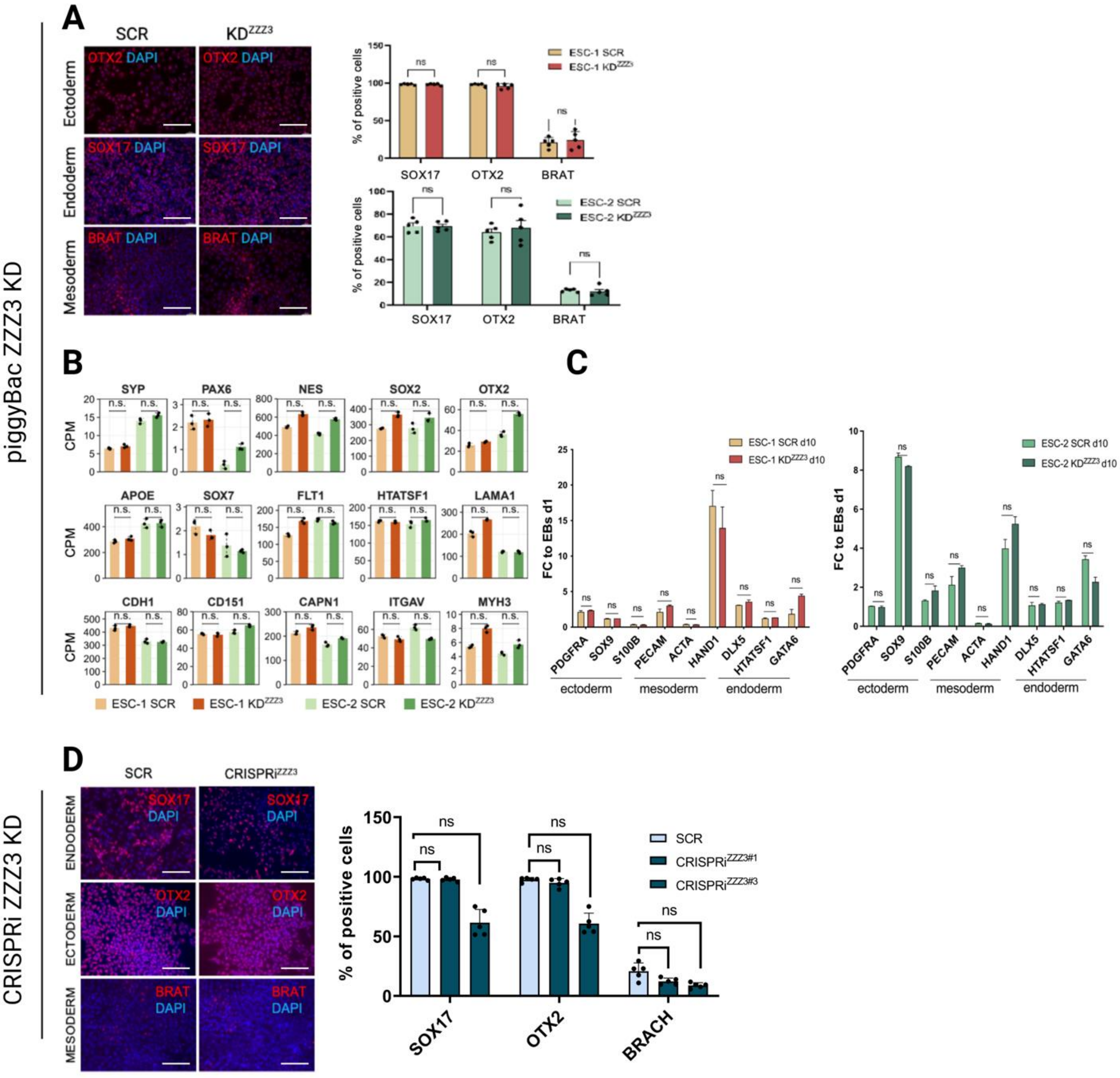
A
SCR
KDZZZ3
Ectoderm
Endoderm
Mesoderm
OTX2 DAPI
SOX17 DAPI
BRAT DAPI
% of positive cells
ESC-1 SCR
ESC-1 KDZZZ3
ESC-2 SCR
ESC-2 KDZZZ3
SOX17
OTX2
BRAT
ns
piggyBac ZZZ3 KD
B
SYP
PAX6
NES
SOX2
OTX2
APOE
SOX7
FLT1
HTATSF1
LAMA1
CDH1
CD151
CAPN1
ITGAV
MYH3
CPM
n.s.
ESC-1 SCR
ESC-1 KDZZZ3
ESC-2 SCR
ESC-2 KDZZZ3
C
ESC-1 SCR d10
ESC-1 KDZZZ3 d10
ESC-2 SCR d10
ESC-2 KDZZZ3 d10
FC to EBs d1
PDGFRA
SOX9
S100B
PECAM
ACTA
HAND1
DLX5
HTATSF1
GATA6
ectoderm
mesoderm
endoderm
D
SCR
CRISPRiZZZ3
CRISPRi ZZZ3 KD
ENDODERM
ECTODERM
MESODERM
SOX17 DAPI
OTX2 DAPI
BRAT DAPI
% of positive cells
SCR
CRISPRiZZZ3#1
CRISPRiZZZ3#3
SOX17
OTX2
BRACH
ns

Figure S3. The expression of the three germ layers is not impaired upon ZZZ3 knockdown. (Related to Fig. 2). A. Immunofluorescence staining was performed to visualize the expression of specific markers indicative of ectoderm, endoderm, and mesoderm differentiation in the SCR control and ZZZ3 KD hESCs. Antibodies targeting key markers - such as OTX2 (ectoderm), BraT (mesoderm), and SOX17 (endoderm) - were used. Nuclei were counterstained with DAPI. Scale bar = 50 µm. Quantification of immunofluorescence images based on the percentage of positive cells was performed using ImageJ software. Data are presented as mean ± standard error of the mean (SEM) from n = 3 independent experiments. The difference observed between the SCR control and ZZZ3 KD hESCs was not statistically significant (ns) (graph on the right). **B**. Barplots show the gene expression levels of a panel of three germ layer regulators in SCR and ZZZ3 KD hESCs as determined by RNA-seq analysis. Mean expression levels ± SEM from three independent experiments is represented by bars, with individual data points overlaid as dots. Statistical analysis was performed using ANOVA, indicating non-significant differences (ns) between the experimental groups. **C**. Quantitative polymerase chain reaction (qPCR) was employed to measure the mRNA expression levels of the three germ layer markers on SCR and ZZZ3 KD EBs at day 10 of differentiation. Non-significant differences (ns) were detected between the experimental groups. **D.** Immunofluorescence staining of specific markers indicative of ectoderm (OTX2), endoderm (SOX17), and mesoderm (BraT) differentiation in the SCR control and ZZZ3 KD hESCs generated via CRISPRi system. Nuclei were counterstained with DAPI. Scale bar = 50 µm. Quantification of immunofluorescence images based on the percentage of positive cells was performed using ImageJ software. Data are presented as mean ± standard error of the mean (SEM) from n = 3 independent experiments. The difference observed between the SCR control and CRISPRi-ZZZ3sh hESCs was not statistically significant (ns) (graph on the right).

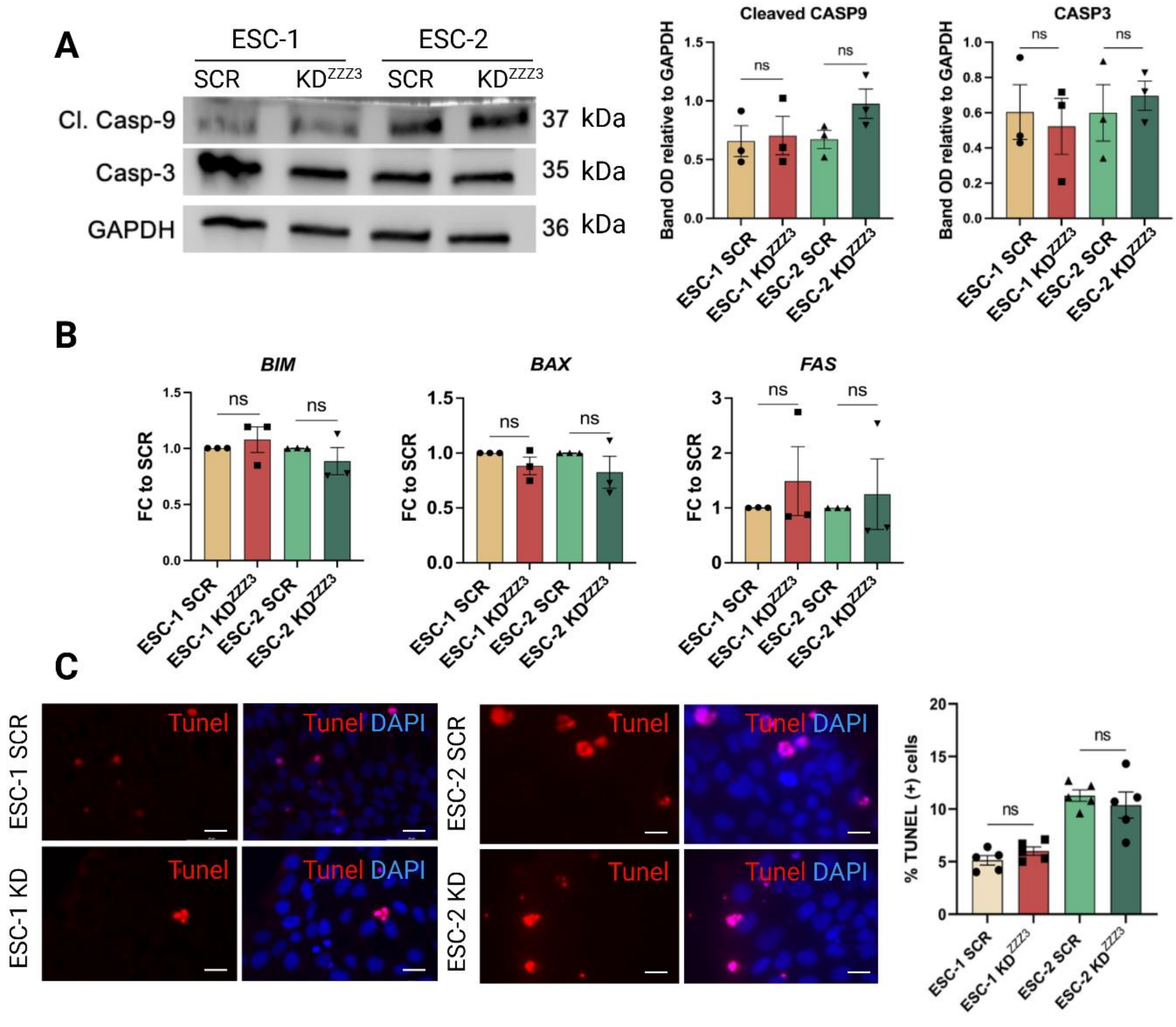
A
ESC-1
ESC-2
SCR
KD ZZZ3
Cl. Casp-9
Casp-3
GAPDH
37 kDa
35 kDa
36 kDa
Cleaved CASP9
CASP3
Band OD relative to GAPDH
ns
ESC-1 SCR
ESC-1 KD ZZZ3
ESC-2 SCR
ESC-2 KD ZZZ3
B
BIM
BAX
FAS
FC to SCR
C
Tunel
Tunel DAPI
ESC-1 SCR
ESC-1 KD
ESC-2 SCR
ESC-2 KD
% TUNEL (+) cells

Figure S4. The decrease in cell proliferation is not attributed to apoptosis. (Related to Figures 3 and 5). A. Western blot analysis was conducted to assess the protein levels of cleaved caspase 9 and caspase 3 in SCR control and ZZZ3 KD hESCs (left); quantification of protein expression levels was performed using optical density (OD) measurement for each immunoblot shown. Data are presented as mean ± standard error of the mean (SEM) from n = 3 independent experiments. Significance was calculated vs. relative SCR ESCs using *t*-test. The difference observed between the groups was not statistically significant (ns) (right). **B**. Quantitative polymerase chain reaction (qPCR) was employed to measure the mRNA expression levels of *BIM*, *BAX*, and *FAS* genes in ZZZ3 KD vs. SCR control. Data are shown as mean ± SEM of three independent experiments and *t*-test was calculated *vs.* SCR cells (ns = not significant). **C**. Immunofluorescence staining for TUNEL was performed to detect DNA fragmentation indicative of apoptosis. Relative quantification was carried out to assess the extent of TUNEL-positive cells or fluorescence intensity. Scale bar: 50 µm. Quantification of immunofluorescence signals was performed using ImageJ software. Data are presented as mean ± standard error of the mean (SEM) from n = 3 independent experiments (at least 200 nuclei were analysed). Significance was calculated vs. relative SCR ESCs using *t*-test, ns = not significant.

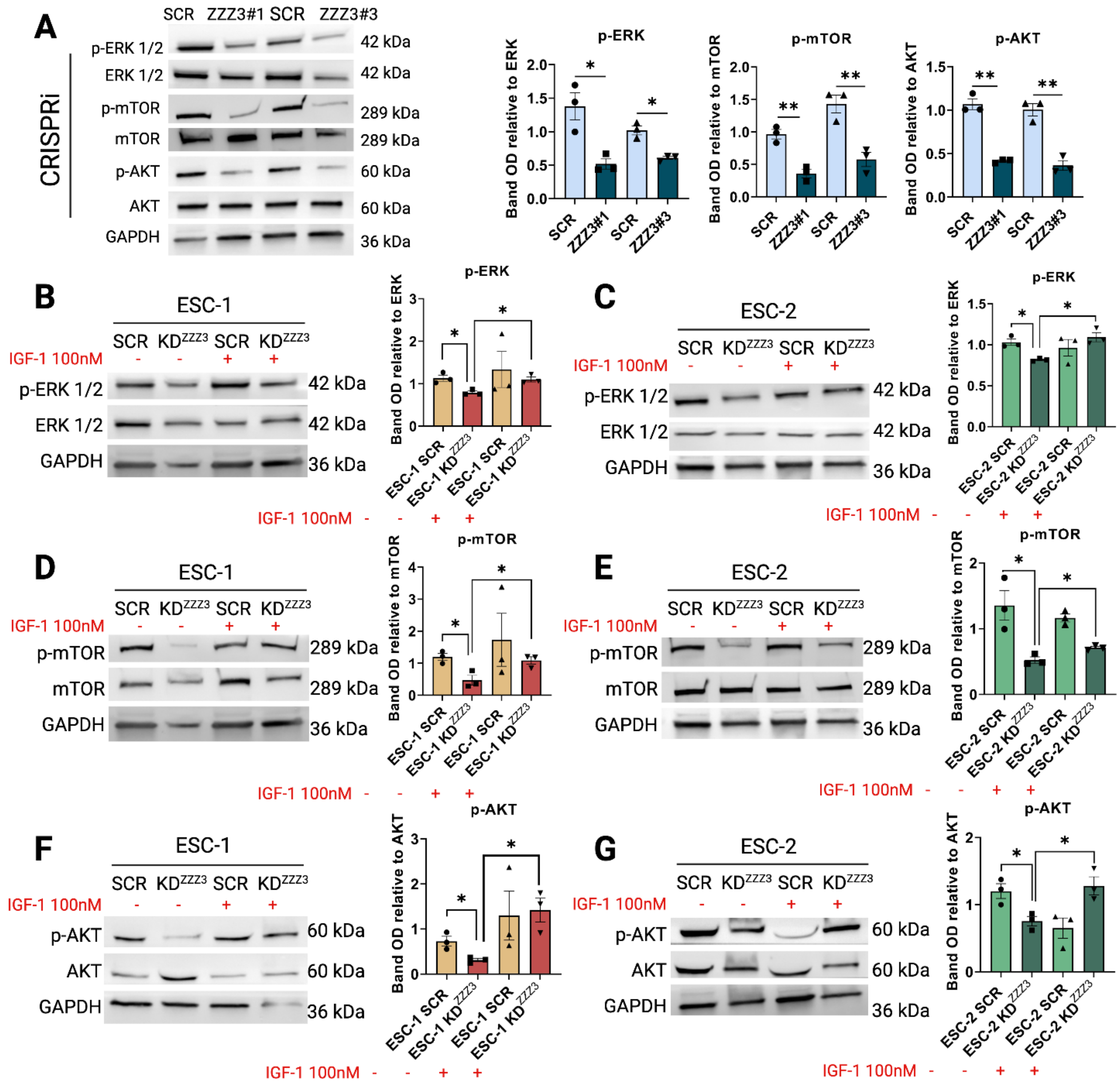
A
CRISPRi
SCR ZZZ3#1 SCR ZZZ3#3
p-ERK 1/2
ERK 1/2
p-mTOR
mTOR
p-AKT
AKT
GAPDH
42 kDa
42 kDa
289 kDa
289 kDa
60 kDa
60 kDa
36 kDa
p-ERK
p-mTOR
p-AKT
Band OD relative to ERK
Band OD relative to mTOR
Band OD relative to AKT
SCR
ZZZ3#1
SCR
ZZZ3#3
B
ESC-1
SCR KDZZZ3 SCR KDZZZ3
IGF-1 100nM - - + +
p-ERK 1/2
ERK 1/2
GAPDH
42 kDa
42 kDa
36 kDa
ESC-1 SCR
ESC-1 KDZZZ3
C
ESC-2
ESC-2 SCR
ESC-2 KDZZZ3
D
E
F
G
p-mTOR
mTOR
p-AKT
AKT
289 kDa
60 kDa

Figure S5. IGF-1 rescues dysregulated PI3K/Akt/mTOR signaling pathways in ZZZ3 knockdown hESCs. (Related to Fig. 6). A. Western blot analysis was used to confirm the reduction of p-ERK 1/2, p-mTOR and p-AKT expression upon knockdown of ZZZ3 using SCR control and ZZZ3 KD hESCs generated via CRISPRi system. Quantification of protein expression levels was performed using optical density (OD) measurement. Data are presented as mean ± SEM from n = 3 independent experiments. Significance was calculated vs. relative SCR ESCs using t-test, * $p \leq 0.05$, ** $p \leq 0.01$. **B-F.** Immunoblot analysis was performed as readout of the rescue of key components of the PI3K/Akt/mTOR Signaling Pathways in ZZZ3 KD hESCs (ESC-1 and ESC-2 cell lines). **B.** and **C.** Representative immunoblots showing the protein expression levels of total ERK and phospho-ERK (Thr202/Tyr204) in ZZZ3 KD ESC-1 and ESC-2 treated and untreated with IGF-1 (100 mM, 30 minutes); **D.** and **E.** Representative immunoblots showing the protein expression levels of total mTOR and phospho-mTOR (Ser2448) in ZZZ3 KD hESCs treated and untreated with IGF-1; **F.** and **G**. Representative immunoblots showing the protein expression levels of total Akt and phospho-Akt (Ser473) in ZZZ3 KD hESCs treated and untreated with IGF-1. Quantification of immunoblot signals reveals a significant restoration of PI3K, Akt, and mTOR phosphorylation levels upon IGF-1 treatment in ZZZ3 knockdown hESCs compared to untreated knockdown cells. Data are presented as mean ± standard error of the mean (SEM) from n = 3 independent experiments. Significance was calculated vs. relative SCR ESCs cells using *t*-test, * $p \leq 0.05$.

SUPPLEMENTARY TABLES

Table S3. Sequences used to induce ZZZ3 knockdown.

- PiggyBac: pPB[shRNA]-Hygro-U6>_shRNA

- TET-KD: pPB [TetOn]-TRE>EGFP: {hZZZ3[miR30-shRNA#1]} -rev (CAG>tTS:T2A:rtTA:P2A:Hygro)

- CRISPRi - sgRNA delivery and transactivation plasmid: pPB-Ins-U6p-sgRNAentry-EF1Ap-TetOn3G-IRES-Neo (the necessary tails in the ends for the cloning are highlighted).

**shRNA Knockdown PiggyBac**	Target Sequence
hZZZ3[shRNA#1]	TCACCAATTAGAACCTATTTA
hZZZ3[shRNA#2]	TCAAAGAACTTGGTCATAAAT
hZZZ3[shRNA#3]	GACGACAGCACCCTCTTAATA
**TET-inducible shRNA**	Sequence
shRNA#1	AGCAGAACACCAAACTTATATA
shRNA#2	CAAAGAACTTGGTCATAAAT
**CRISPRi - gRNA**	Sequence
ZZZ3_sgRNA #1	Forward (5’ → 3’) **CACC**ACAGACGATTGCTGTACTCG<br>Reverse (5’ → 3’) **AAAC**CGAGTACAGCAATCGTCTGT
ZZZ3_sgRNA #2	Forward (5’ → 3’) **CACC**TGGGTAAGCCCTAGGAAAAG<br>Reverse (5’ → 3’) **AAAC**CTTTTCCTAGGGCTTACCCA
ZZZ3_sgRNA #3	Forward (5’ → 3’) **CACC**GAGCCTGTGCCAATTCAGAA<br>Reverse (5’ → 3’) **AAAC**TTCTGAATTGGCACAGGCTC
ZZZ3_sgRNA #4	Forward (5’ → 3’) **CACC**AAGACCTTGAAAGTTTAGGC<br>Reverse (5’ → 3’) **AAAC**GCCTAAACTTTCAAGGTCTT

Table S4. List of antibodies used for Western Blot (WB) and Immunofluorescence (IF) analyses.

Antibody	Host species	Dilution	Cat.No	Company	Application
ZZZ3	Rabbit	1:2000	ab118800	Abcam	WB
Nanog	Goat	1:500	AF1997	R&D System	WB
Oct4	Rabbit	1:1000	bs-1111R	Bioss Antibodies	WB
pERK1/2 (Thr202/Tyr204)	Rabbit	1:1000	9101	Cell Signaling Technology	WB
ERK1/2	Mouse	1:1000	9107	Cell Signaling Technology	WB
p53	Mouse	1;500	sc-393031	Santa Cruz Biotechnology	WB
RPL19a	Mouse	1:1000	sc-100830	Santa Cruz Biotechnology	WB
Phospho-RPS6 (S235)	Rabbit	1:1000	ab227005	Abcam	WB
Ribosomal Protein S6	Mouse	1:2000	sc-74459	Santa Cruz Biotechnology	WB
Phospho-Akt (Ser473) (D9E)	Rabbit	1:2000	4060S	Cell Signaling Technology	WB
Akt	Rabbit	1:1000	9272S	Cell Signaling Technology	WB
Phospho-mTOR (Ser2448)	Rabbit	1:1000	5536S	Cell Signaling Technology	WB
mTOR	Rabbit	1:1000	2983S	Cell Signaling Technology	WB
Phospho-4E-BP1 (Thr37/46)	Rabbit	1:1000	2855	Cell Signaling Technology	WB
4E-BP1	Rabbit	1:1000	9452	Cell Signaling Technology	WB
Phospho-eIF4E(Ser209)	Rabbit	1:1000	9741	Cell Signaling Technology	WB
eIF4E	Rabbit	1:1000	9742	Cell Signaling Technology	WB
Phospho-p70 S6 Kinase (Thr421/Ser424)	Rabbit	1:1000	9204	Cell Signaling Technology	WB
p70 S6 Kinase	Rabbit	1:1000	2708	Cell Signaling Technology	WB
Cleaved Caspase-9	Rabbit	1:1000	9505	Cell Signaling Technology	WB
Caspase-3 (3G2)	Mouse	1:1000	9668	Cell Signaling Technology	WB

Antibody	Host species	Dilution	Cat.No	Company	Application
c-myc	Rabbit	1:1000	5605s	Cell Signaling Technology	WB
E2F4	Rabbit	1:1000	40291	Cell Signaling Technology	WB
Fibrillarin	Mouse	1:2000	ab4566	Abcam	WB
DDX18	Rabbit	1:2000	A300-636-A	Bethyl	WB
GAPDH	Rabbit	1:1000	bs10900R	Bioss Antibodies	WB
Actin	Goat	1:500	sc1616	Santa Cruz Biotechnology	WB
Peroxidase AffiniPure Donkey Anti-Rabbit IgG (H+L)		1:10000	711-035-152	Jackson Immuno Reaserch	WB
Peroxidase AffiniPure Sheep Anti-Mouse IgG (H+L)		1:10000	515-035-062	Jackson Immuno Reaserch	WB
Peroxidase AffiniPure Rabbit AntiGoat IgG (H+L)		1:5000	305-035-045	Jackson Immuno Reaserch	WB
ZZZ3	Rabbit	1:100	Pa5-84224	Invitrogen	IF
Fibrillarin	Mouse	1:500	ab4566	Abcam	IF
Nanog	Goat	1:200	AF1997	R&D System	IF
Oct4	Mouse	1:200	75463	Cell Signaling Technology	IF
Ki67	Rabbit	1:400	9129S	Cell Signaling Technology	IF
p21	Rabbit	1:400	2947S	Cell Signaling Technology	IF
Anti-Rabbit IgG Alexa Fluor 594		1:500	A-11012	Invitrogen	IF
Anti-Rabbit IgG Alexa Fluor 488		1:500	A-11008	Invitrogen	IF
Anti-Goat IgG Alexa Fluor 594		1:500	A-11058	Invitrogen	IF
Anti-Mouse IgG Alexa Fluor 488		1:2000	A-11001	Invitrogen	IF

Table S5. Primers used for quantitative PCR analysis.

Gene	Primer sequence
GAPDH	For_ TCCTCTGACTTCAACAGCGA
	Rev_ GGGTCTTACTCCTTGGAGGC
OCT4	For_ GGAGGAAGCTGACAACAATGAA
	Rev_ GGCCTGCACGAGGGTTT
NANOG	For_ TGCAAGAACTCTCCAACATCCT
	Rev_ ATTGCTATTCTTCGGCCAGTT
SOX2	For_ ATGCACCGCTACGACGTGA
	Rev_ CTTTTGCACCCCTCCCATTT
PDGFRA	For_ TAATGACTCACCTGGGGCCA
	Rev_ CTTTGGCTTCTCTGGGTGGT
SOX9	For_ AGCTCTGGAGACTTCTGAACGAGA
	Rev_ CGTTCTTCACCGACTTCCTCCGC
S100B	For_ CATCGACGTTTTCCACCAAT
	Rev_ GAAGTCACATTCGCCGTCTC
PECAM	For_ ATGCCGTGGAAAGCAGATAC
	Rev_ CTGTTCTTCTCGGAACATGGA
ACTA	For_ TCTGGCACCACACCTTCTACAATG
	Rev_ AGCACAGCCTGGATAGCAACG
HAND1	For_ CCAGCTACATCGCCTACCTG
	Rev_ CCGGTGCGTCCTTTAATCCT
DLX5	For_ TTCAGAAGACTCAGTACCTCGC
	Rev_ GAGTTACACGCCATTGGGTC
HTATSF1	For_ ACGATGGCGCATCTAGTTCT
	Rev_ ATCTGGAGGCAAACCAGACA
GATA6	For_ AGCGACTCCAGAGCCTTTC
	Rev_ATGCGAAGCGTAGGAACTGA
BIM	For_ GCTGTCTCGATCCTCCAGTG
	Rev_ TCCAATACGCCGCAACTCTT
BAX	For_ GGACGAACTGGACAGTAACATGG
	Rev_ GCAAAGTAGAAAAGGGCGACAAC

FAS	For_ GGAGTACACAGACAAAGCCCA
	Rev_ TTTGGTGCAAGGGTCACAGT

EXPERIMENTAL PROCEDURES

Cell culture. The human ES cell-lines (WA17 and RUES2; WiCell Research Institute, Madison, WI) were maintained in mTeSR1 Plus medium (STEMCELL Technologies, Vancouver, Canada) on plates coated with Matrigel (BD Biosciences, San Diego, CA) in a humidified incubator at 37°C and 5% CO_2. Medium was replaced every two days and cells were split every 5 to 6 days using Gentle Dissociation reagent (STEMCELL Technologies, Vancouver, Canada). hES cells were routinely tested for Mycoplasma using the Mycoplasma PCR Detection kit (Applied Biological Materials, Richmond, Canada).

Cell proliferation assay. Human ESCs were plated onto Matrigel-coated 12-well plates at a density of $3x10^4$ cells per well and cultured in mTeSR1 Plus medium for specified durations (24h, 48h, 72h, and 96h). Subsequently, cells were detached using Accutase (Thermo Fisher Scientific) and counted using a cell counting chamber. Each experiment was repeated independently three times.

MTT assay. 5,000 hES cells per well were seeded in triplicate onto a 96-well plate matrigel-coated. At 80% confluence, cells were treated with 0.5 mg/ml of 3-[4,5-dimethylthiazol-2-yl] -2,5-diphenyl-tetrazolium bromide (MTT) for 2 hours. Subsequently, the MTT solution was replaced with 2-propanol (Sigma-Aldrich) and agitated for 10 minutes. Absorbance was then measured at 570 nm using a Varioskan LUX plate reader (Thermo Fisher Scientific).

Synchronization and cell cycle profile analysis. SCR control and ZZZ3 knockdown ESCs were synchronized by treating them with nocodazole (100 ng/ml, Thermo Fisher Scientific) for 12 hours, causing their accumulation in the G2/M phase. Cell cycle analysis was conducted by harvesting cells immediately after nocodazole treatment (T0) and 8 hours after nocodazole withdrawal (T1). For cell profile analysis, cells were collected by centrifugation, washed with ice-cold PBS, and fixed with 70% ethanol overnight at 4°C. Following fixation, the cells were washed with PBS, resuspended in PBS containing propidium iodide (50 µg/ml, Thermo Fisher Scientific), RNAsi (100 µg/ml, Thermo Fisher Scientific), and NP40 (0.01%, Santa

Cruz Biotechnology), and incubated for 1 hour in darkness. ZZZ3 knockdown hESCs generated with piggyBac encoding for doxycycline-inducible ZZZ3 shRNA (DoxKD) were utilized to rescue the proliferation defect (Dox^{+}/Dox^{-}). Flow cytometry analysis was performed on the BD LSRFortessa x-20 Flow Cytometer, and data was processed using FlowJo software.

TUNEL assay. For *in vitro* apoptosis detection, ESCs were initially seeded into matrigel-coated 8-well chamber slides. The cells were then fixed using 3.7% (vol/vol) formaldehyde (Sigma-Aldrich) and processed with the Click-iT™ Plus TUNEL Assay Kit AlexaFluor 594 (Thermo Fisher Scientific) following the manufacturer's instructions. After completing the assay, the cells were mounted with Dako Fluorescent Mounting Medium (Agilent), and images were captured using Leica microscopy systems (DMi8) equipped with Leica LAS X software (version 3.7.4.23463). TUNEL-positive cells were manually quantified using ImageJ software.

Immunofluorescence staining. Immunofluorescence analysis was performed on Matrigel-coated glass coverslip in wells. Cells were fixed in 3.7% (vol/vol) formaldehyde for 15 minutes at room temperature, washed in PBS, permeabilized for 1h at RT in PBS + 0.3% Triton X-100 (Sigma-Aldrich) (PBST), and blocked for 1h at RT in PBST containing 10% of fetal bovine serum (FBS) (Thermo Fisher Scientific). Cells were subjected to immunostaining overnight at 4°C with primary antibodies (Table S4) diluted in blocking solution. After washing with PBS, cells were incubated with Alexa Fluor 488- or 594- conjugated secondary antibodies (Alexa, Life Technologies) for 1h at RT. Nuclei were stained with DAPI (4′,6-diamidino-2-phenylindole) (Thermo Fisher Scientific). Finally, cells were mounted with DAKO Fluorescent Mounting Medium (Agilent), and images were acquired using Leica microscopy systems (DMi8, Thunder DMi8, and Stellaris 5 confocal) and Leica LAS X software (v.3.7.4.23463). All immunostainings' analyses were performed using ImageJ software. A list of antibodies used for immunostaining is provided in (Supplementary Table S4).

Western blot analysis. Cells were washed with cold PBS and then scraped from the plate using RIPA buffer (150mM Sodium Chloride, 1% Triton x-100, 0.5% sodium deoxycholate, 0.1% SDS (sodium dodecyl sulfate), 50mM Tris hydrochloride, pH 8.0), supplemented with Halt™ Protease Inhibitor and Halt™ Phosphatase Inhibitor Cocktails (Thermo Fisher Scientific). The protein content was determined using Bradford (Bio-Rad) protein assay. Equal amounts of proteins (30-50 μg) were denatured in Laemmli sample buffer at 95°C for 5 minutes, separated on 4-20% Mini-PROTEAN TGX precast gels (Bio-Rad), and transferred onto nitrocellulose membranes (Bio-Rad) using a Trans-Blot® Turbo™ Transfer System (Bio-Rad). Blots were blocked in 5% non-fat milk for 1 h at room temperature and subsequently subjected to overnight primary antibody incubation at 4°C, followed by two quick rinses and three washes for 5 min in PBST (PBS + 0.1% Tween-20). Secondary antibody incubation was performed for 1h at RT. Clarity™ Western ECL Blotting Substrates (Bio-Rad) was used to detect the HRP signal and the western blot images were collected using the Alliance™ Q9-Atom (Uvitec). For rescuing the PI3K-AKT-mTOR defect, both SCR and ZZZ3 knockdown ESCs were treated with 100 nM of insulin-like growth factor (IGF-1) (Sigma-Adrich) for 30 minutes. Uncropped western blots images are shown in File S1. The details regarding specific antibodies used can be found in Table S4.

RNA extraction, reverse transcription, and quantitative real-time PCR. Total RNA was extracted using TRIzol Reagent (Thermo Fisher Scientific) following manufacturer's instructions. Reverse transcription was performed using High-Capacity cDNA Reverse Transcription Kit (Thermo Fisher Scientific). Quantitative PCR analyses were performed in real time using a QuantStudio™ 7 Pro Real Time PCR system (Applied Biosystem) and SensiFAST SYBR Hi-ROX kit (Meridian Bioscience). Gene expressions were calculated following normalization to *GAPDH* (Glyceraldehyde 3-phosphate dehydrogenase) levels using the comparative Ct (cycle threshold) method. Statistical differences were calculated using two-tailed *t*-test or multiple unpaired *t*-test with Welch correction, with a significance of * $p \leq 0.05$, ** $p \leq 0.01$, and *** $p \leq 0.001$. Data are presented as mean ± SEM from three independent experiments. The primer sequences utilized in the qRT-PCR analysis are provided in the Supplementary Table S5.

Nucleolar staining. Nucleoli were stained using the NUCLEOLAR-ID® Green Detection Kit (ENZO) following manufacturer's instructions. The hESCs were grown on coverslips placed inside a Petri dish filled with the mTeSR1 culture medium. At 80% confluence the media was removed and a sufficient volume of NUCLEOLAR-ID® Green Detection Reagent was dispensed onto the monolayer of cells in darkness. Cells were incubated for 15-30 minutes at 37°C. After the incubation period, cells were washed with 100 µL of 1X Assay Buffer. Excess buffer was removed and the stained cells were analyzed using a Stellaris5 confocal microscopy (Leica Microsystems). For imaging the nucleolus, a standard FITC filter set was utilized. Hoechst 34580 dye (Invitrogen) was used to label nuclear DNA.

Polysome profile. Before harvesting, cells were washed with ice-cold PBS supplemented with 100 µg/mL cycloheximide and resuspended in 1 mL lysis buffer (10 mM Tris-HCl pH7.4, 100 mM KCl, 10 mM MgCl2, 1% Triton-X 100, 1 mM DTT, 10 U/mL RNAseOUT (Invitrogen), 100 µg/mL of cycloheximide), and scraped. After 5 min of incubation on ice, cell lysate was centrifuged for 10 min at 14,000 rpm at 4°C. The supernatant was collected and protein content was determined by Bradford analysis (Bio-Rad). Equal protein amounts (4.2 mg) were loaded onto a 10-60% sucrose gradient obtained by adding 6 mL of 10% sucrose over a layer of 6 mL of 60% sucrose prepared in lysis buffer without Triton and containing 0.5 mM DTT, in a 12-mL tube (Polyallomer; Beckman Coulter). Gradients were prepared using a gradient maker (Gradient Master, Biocomp). Polysomes were separated by centrifugation at 37,000 rpm for 2.5 hours using a Beckman SW41 rotor. Twelve fractions of 920 µL were collected while polysomes were monitored by following the absorbance at 254 nm. Total protein was retrieved by 100% ethanol (EtOH) precipitation performed overnight, washed twice with 70% EtOH and analyzed by SDS-PAGE followed by Western blot.

DDA data analysis. Raw files were processed in MaxQuant software (version 2.0.1.0) using the Andromeda search engine. The MS/MS spectra were searched against a human proteome database (downloaded in March 2016 and containing 42.013 sequences). For label free quantification (LFQ) analysis the following settings were used: Carbamidomethylation of cysteines as static modification, and oxidation of methionine and protein N-terminal acetylation as variable modifications. High confidence and unique peptides (minimum 1 peptide

per protein group) were used for protein identification. Further parameters were set as follows: first and main search peptide tolerance, respectively 20 ppm and 4.5 ppm; isotope and centroid match tolerance, respectively 2 and 8 ppm; maximum number of missed cleavages, 2. Match between runs (MBR) option was activated, with match-time window set at 0.7 min and initial alignment window at 20 min. Only unique peptides were selected for quantification with a minimum ratio count of 1. For statistical analysis of MaxQuant output, the Perseus software (version 2.0.6.0) was used as follows: the LFQ intensity of proteins from the MaxQuant analysis were imported and contaminants, reverse identification, and proteins only identified by site were excluded from further data analysis. Data were transformed in logarithmic scale (log2). After filtering (at least three valid LFQ values in at least one group), remaining missing LFQ values were imputed from a normal distribution (width, 0.3; down shift, 1.8). Finally, for all the data sets, paired two-sample t-test was used to assess statistical significance of protein abundances using a 5% permutation-based FDR adjustment. Based on statistical measures described, we detected 525 interacting proteins associated with ZZZ3 in ESC1 and 922 interacting proteins in ESC2. To narrow down potential interactors of ZZZ3, we focused on those exhibiting fold changes of ≥ 2.5 (n = 474 in ESC-1 and n = 744 in ESC-2). The mass spectrometry proteomics data have been deposited to the ProteomeXchange Consortium via the PRIDE (Perez-Riverol et al., 2022) partner repository.

LIST OF OTHER SUPPLEMENTARY FILES:

File S1. Uncropped full-length western blots.

Table S1. Interactome data and Gene Ontology

Table S2. Differentially expressed genes (DEGs)_RNA-Seq ZZZ3 KD ESCs *vs*. SCR control ESCs.
